# Technological Maturity of Aircraft-Based Methane Sensing for Greenhouse Gas Mitigation

**DOI:** 10.1021/acs.est.4c02439

**Published:** 2024-05-17

**Authors:** Sahar H. El Abbadi, Zhenlin Chen, Philippine M. Burdeau, Jeffrey S. Rutherford, Yuanlei Chen, Zhan Zhang, Evan D. Sherwin, Adam R. Brandt

**Affiliations:** † Department of Energy Science & Engineering, Stanford University, Stanford, California 94305, United States

**Keywords:** remote sensing, controlled release, methane, oil and gas, climate change, energy

## Abstract

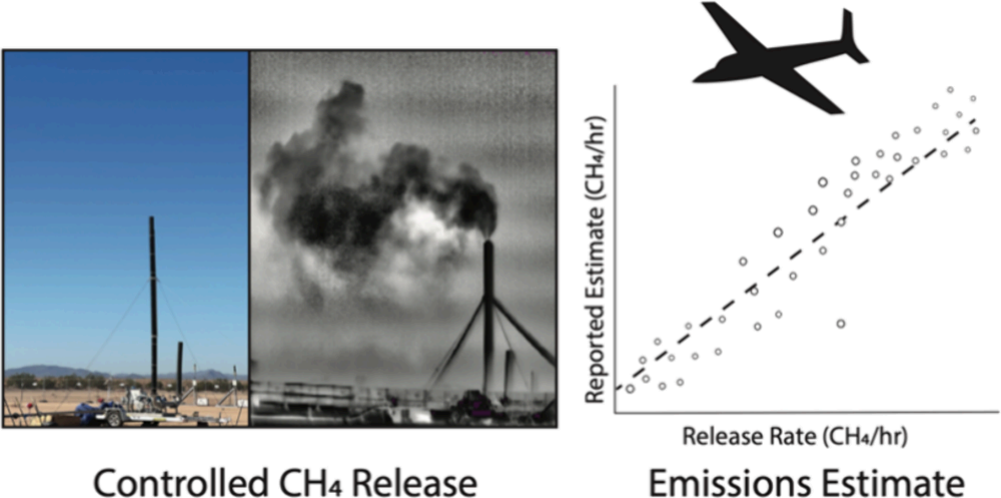

Methane is a major contributor to anthropogenic greenhouse gas emissions. Identifying large sources of methane, particularly from the oil and gas sectors, will be essential for mitigating climate change. Aircraft-based methane sensing platforms can rapidly detect and quantify methane point-source emissions across large geographic regions, and play an increasingly important role in industrial methane management and greenhouse gas inventory. We independently evaluate the performance of five major methane-sensing aircraft platforms: Carbon Mapper, GHGSat-AV, Insight M, MethaneAIR, and Scientific Aviation. Over a 6 week period, we released metered gas for over 700 single-blind measurements across all five platforms to evaluate their ability to detect and quantify emissions that range from 1 to over 1,500 kg(CH_4_)/h. Aircraft consistently quantified releases above 10 kg(CH_4_)/h, and GHGSat-AV and Insight M detected emissions below 5 kg(CH_4_)/h. Fully blinded quantification estimates for platforms using downward-facing imaging spectrometers have parity slopes ranging from 0.76 to 1.13, with *R*^2^ values of 0.61 to 0.93; the platform using continuous air sampling has a parity slope of 0.5 (*R*^2^ = 0.93). Results demonstrate that aircraft-based methane sensing has matured since previous studies and is ready for an increasingly important role in environmental policy and regulation.

## Abstract 2

An independent study shows the technological maturity of aircraft-based methane leak sensing, demonstrating readiness of this approach for use in greenhouse gas policy and regulation.

## 1 Introduction

Methane is a potent greenhouse gas with over 80 times the global warming potential of carbon dioxide over a 20 year timespan.^1^ With a short atmospheric lifetime, methane shapes near-term climate outcomes, making it a priority for climate change mitigation efforts. Top anthropogenic methane sources and targets for emissions reductions are the oil and gas sector, waste management, and agriculture.^2^ Conventional methods for detecting and quantifying methane emissions are time and labor intensive, with limited scalability due to reliance on individual site visits with methane detectors or optical gas imaging infrared cameras.^3^ Greenhouse gas inventories continue to use a so-called “bottom-up” method for estimating emissions, which underestimate emissions from the oil and gas sector compared to results from measurement surveys.^4^ In recognition of these shortcomings, new approaches for detecting methane emissions are in development and are currently being deployed, including aircraft- and satellite-based methods.

Aircraft-based methane sensing enables the rapid and widespread assessment of methane emissions. In the last several years, aerial surveys have identified methane leaks several-fold larger than those reported in greenhouse gas inventories or found using conventional ground-based surveys.^5−10^ Sherwin et al. find that in multiple oil and gas producing regions across the United States, aerially detected emissions from roughly 1% of sites constitute 50–80% of total methane emissions from oil and gas production, processing, and transportation infrastructure, highlighting the prospect of massive emissions reductions through aerial surveys.^10^ Following these technical advances, US Environmental Protection Agency has proposed new rules that, if adopted, would allow companies to use remote sensing technologies, including aircraft, to comply with emissions monitoring and reduction efforts at oil and gas production sites.^11^

Methane-sensing aircraft typically use one of two approaches for quantifying methane emissions: infrared spectroscopy and in situ methods. Spectroscopy uses the differential absorption of infrared (IR) light by methane compared with other atmospheric gases. Imaging is most commonly passive, relying on reflected sunlight as a radiation source and thus requiring favorable weather conditions. An alternative approach is the active spectroscopy LiDAR system, in which a laser mounted within the aircraft sends a radiation signal that is reflected and used in analysis.^3^ For the in situ approaches, an aircraft measures atmospheric concentrations of methane in real time during the flight, and emission magnitude is quantified using models that combine multiple concentration measurements with flight altitude and distance from the target.^12^ While time-intensive compared to imaging, in situ approaches allow for analysis of other air pollutants alongside methane, including carbon dioxide, nitric oxide, and nitrogen dioxide.^13^ While detection capabilities vary by platform and technological approach, under ideal measurement conditions, aircraft can detect emissions below 100 kg (CH_4_)/h, and in some cases, below 10 kg (CH_4_)/h.^3,14,15^ In contrast, most wide-area satellite have a detection limit around 1,000–1,500 kg(CH_4_)/h, although targeted systems such as the GHGSat satellite and Maxar’s WorldView-3 have detected emissions under, ideal conditions, as low as 200 and 30 kg(CH_4_)/h, respectively.^16,17^

As companies and governments increasingly rely on aircraft methane management, accurately assessing these technologies’ capabilities becomes increasingly important. Here, we report an independent, single-blind evaluation of five different aircraft operators. We examine their ability to identify high-volume methane emissions from a point source. Four operators use passive IR spectroscopy: Carbon Mapper, GHGSat-AV, Insight M (formerly Kairos Aerospace), and MethaneAIR. We also test Scientific Aviation, which uses an in situ measurement approach.

Prior studies have evaluated the performance of aircraft-based methane detection and quantification. Carbon Mapper, GHGSat-AV, Insight M, and MethaneAIR participated in previous Stanford led singe-blind controlled release experiments.^3,15,18^ These operators sought additional validation for new testing configurations or modifications informed by their previous results. While not included in the present study, Bridger Photonics’ Gas Mapping LiDAR has been independently tested elsewhere in single-blind and location-blind studies.^3,14,19^

This study fills important gaps in previous literature. In particular, this is the first independent single-blind test of Scientific Aviation and MethaneAIR (Chulakadabba et al., 2023 ^18^ used a collaborative technology validation experimental design in which the MethaneAIR team had editorial control over the publication of their results, with input from Stanford). In addition, the Insight M and GHGSat-AV systems presented here represent a significant advancement over those tested previously. Finally, this is the first single-blind evaluation of a field-realistic deployment of the Carbon Mapper system, as the previous Stanford test was conducted with shorter flightlines than used in field deployment, resulting in artificially low quantification estimates.^3,20^ Additionally, we test these five platforms under consistent conditions (at the same field site, over a 6-week time period) using all-new gas release hardware and improved data postprocessing. Thus, to date, this work is the assessment most representative of field deployment for the five tested airborne methane sensing systems, which constitute a majority of currently deployed technology systems in this space.

## 2 Methods

We conducted aircraft testing from October 10 to November 11, 2022 in Casa Grande (Arizona) as part of a 2 month experiment that also tested satellites and ground sensors. For intercomparison purposes, we use established experimental and data reporting protocols.^3,15^ Briefly, the Stanford field team releases a fixed stream of methane at a constant rate, while an aircraft operator conducts measurements. We maintain strict blinding protocols: operators are not informed whether a release is being conducted or not. Participants are provided the coordinates of gas release in advance and asked to mimic standard field operations as closely as possible in both data collection and analysis. Additional information describing data collection is provided in the Supporting Information, Section 1.1.

### 2.1 Methane Controlled Releases Equipment

**Figure 1:**
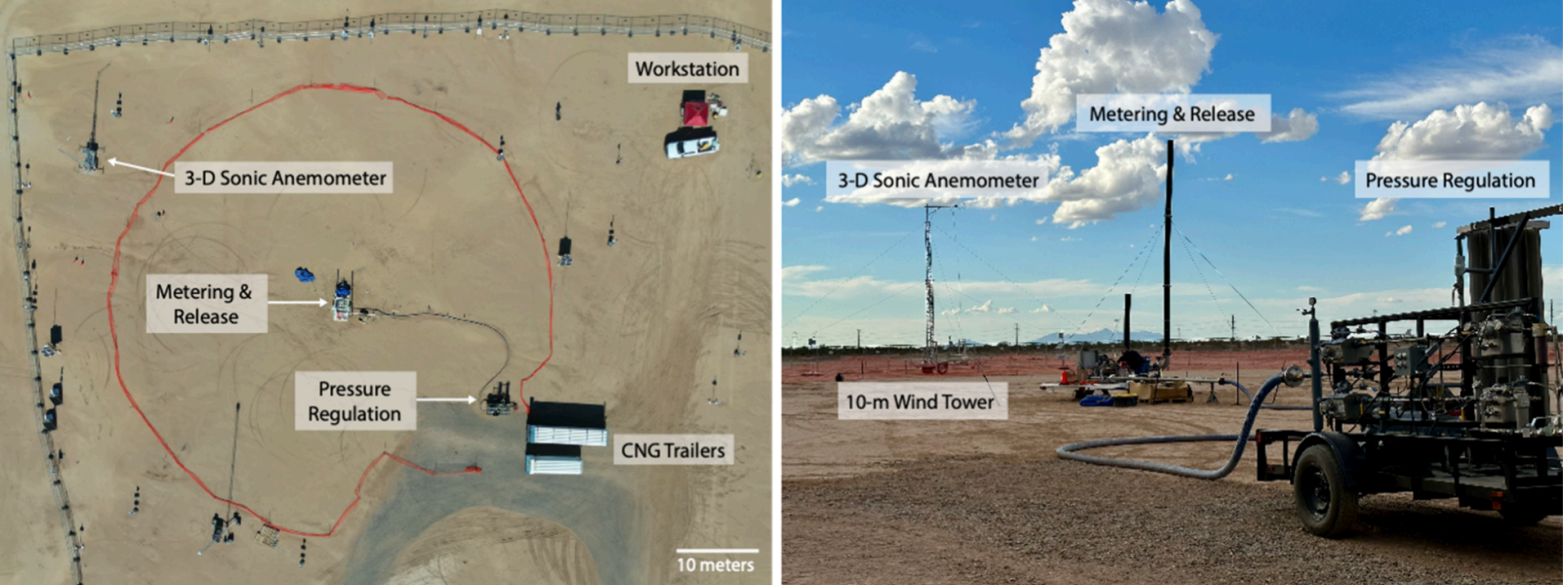
Experimental field setup top view (left) and on-the-ground (right). Methane supply is from compressed natural gas trailers (depicted in the left image only). Gas pressure is reduced in a pressure regulation trailer, then delivered to a metering and release trailer. Wind data is collected using a 3D sonic anemometer mounted on a 10-m wind tower. Stanford set desired flow rates from the workstation. Also visible in the image but not labeled are ground sensor that were deployed during testing.

Gas is released from a trailer parked at a fixed location [32.8218489°, −111.7857599°]. The trailer is equipped with high-precision meters and two stacks that release gas at 7.3 m (24 feet) and 3.0 m (10 feet) above ground level. We refer to these as the tall and short stacks, respectively. The methane source for all experiments was compressed natural gas (CNG), stored on-site in two trailers provided by Rawhide Leasing and refilled from Arizona-based CNG providers as needed. Gas was transferred from the CNG trailers to a pressure regulation trailer (Rawhide Leasing, RT-30) and then to the gas metering trailer, as depicted in Figure 1.

Upon entering the metering and release trailer, gas is diverted through one of three parallel flow paths based on the desired release rate. The three flow paths are designed to release flow rates of 1–30, 30–300, and 300–2000 kg gas/hour (kg/h) and are each fitted with an Emerson Micromotion Coriolis meter sized accordingly. The Stanford team used a laptop to remotely set the flow rate from the field workstation (additional details on flow control in SI Section S1.1.3.1).

#### 2.1.1 Safety

We established a 45 m (150 ft) safety perimeter around the gas release point, and no Stanford personnel were allowed within this perimeter while gas was flowing. Experienced and safety-certified gas contractors (Rawhide Leasing) operated the gas release equipment, and the Stanford team regularly monitored the plume with an infrared camera (FLIR GF320) to ensure methane remained far from all onsite personnel. The team also remained vigilant to olfactory signals of natural gas.

### 2.2 Description of Aircraft-Based Technologies Tested

We tested five different aircraft-based methane-measurement technologies: Carbon Mapper, GHGSat-AV, Insight M, MethaneAIR, and Scientific Aviation. Details of each platform are included in the Supporting Information. Briefly, Carbon Mapper, GHGSat-AV, Insight M, and MethaneAIR all use passive infrared spectroscopy. Carbon Mapper, GHGSat-AV, and Insight M conduct surveys that identify and quantify large-scale methane point source emissions, particularly from oil and gas (examples include but are not limited to those referenced here^5,6,21^). MethaneAIR, the aircraft precursor to MethaneSAT, is designed for wider spatial coverage and measuring diffuse sources in addition to point source.^18^ Scientific Aviation uses a in situ measurement technique, conducting multiple consecutive loops around the target methane source while collecting ambient air samples.^12^ Methane measurements are conducted onboard using a Picarro 2210-m instrument that measures methane, ethane, carbon dioxide, and water. All five aircraft operate at different altitudes and implemented different flight patterns during testing (see Table 1). Hence, the time necessary to conduct a single measurement varies across operators, as well as the total number of measurements feasible in 1 day.

**Table 1:** Summary of the Testing and Flight Conditions

	**Carbon Mapper**	**GHGSat-AV**	**Insight M**	**MethaneAIR**	**Scientific Aviation**
testing dates (month/day format)	10/10 – 10/12, 10/28–10/29, 10/31	10/31, 11/02, 11/04, 11/07	10/24 – 10/28	10/25, 10/29	11/08, 11/10, 11/11
range of flight height above target (meters or feet above ground level)^a^	3,050–3,230 m (10,000–10, 600 ft)	1,930–2,080 m (6,320–6,840 ft)	370–540 m (1,210–1,770 ft)	12, 690–13,610 m (41, 620–44, 670 ft)	60–700 m (200–2,300 ft)
average measurement frequency^b^	12 min	4 min	3 min	22 min	21 min
wind reanalysis data source for fully blinded submission^c^	HRRR	GEOS-FP	Dark Sky	DI method: HRRR; mIME method: HRRR/LES	in-flight measured horizontal windspeed

a For imaging technologies, flight altitude is the average for the 1 min leading up to measurement timestamp. Measurement timestamp refers to the moment when the aircraft distance from the release target was at a minimum, using GPS coordinates. For Scientific Aviation, altitude varies over the course of a 20 min period in which measurements are conducted; here we include the measurement altitudes reported by the operations team to Stanford.

b For imaging technologies, this is the average time between individual measurement timestamps across all flight days for a given aircraft. The measurement time itself is instantaneous, and differences in measurement frequency reflect operator specific flight patterns. For Scientific Aviation, measurement frequency represents the average time for conducting one complete measurement.

c Wind reanalysis data source abbreviations: HRRR = High-Resolution Rapid Refresh (provided by US National Oceanic & Atmospheric Administration); GEOS-FP = Goddard Earth Observing System Forward Processing (provided by US National Aeronautic and Space Administration); for MethaneAIR, LES refers to 1-way coupled Large Eddy Simulation. For Scientific Aviation, windspeed was calculated using methods described in Conley et al.,^12^ which combine GPS coordinates with standard aircraft pitot-static pressure airspeed measurement.

### 2.3 Field Data Collection Procedures

Field measurement protocols were based on those previously reported^3,15,18^ to maintain consistency and comparability with other testing results. Briefly, operators were asked to recreate typical flight operations and submit measurement frequency, planned flight lines, altitude, and predicted lower detection limit in advance. For spectroscopy-based platforms, we held a constant release rate, while the aircraft passed overhead. The Stanford ground-team tracked the GPS location of each aircraft, aiming to change the release rate at least two min before the aircraft next passed overhead. For Scientific Aviation, we set a measurement schedule in advance and held a constant release rate for 35–40 min. Details on field data collection are included Supporting Information Section 1.3.

### 2.4 Data Collection and Filtering

We collected raw 1 Hz flow measurement data from all three Coriolis meters, and data cleaning is described in detail in the Supporting Information, Section 1.2. To convert the whole gas flow rate to methane, we use gas compositional data provided by the upstream supplier of the CNG station from which we purchased natural gas (additional details in the Supporting Information, Section 1.2.3.). Mean mol % CH_4_ over the study period is 94.53%, and the standard deviation is 0.62%.

Wind conditions varied widely during the testing period. Aircraft operators reported observing stagnant methane from previous releases pooling around the site under some conditions. To ensure each new measurement occurred with a clean background, we developed a wind-based filtering criteria for spectroscopy-based operators, which excludes measurements where it is likely that a significant residual signal from the previous measurement might be present. A full description is included in SI section 1.3.5.1. For Scientific Aviation, we excluded any measurements where the standard deviation of the gas flow rate over the measurement period was greater than 10% of the mean flow rate for the same period. This quality control ensures low variability when a release rate must be held for an extended measurement period and removed one measurement from analysis, in which mechanical disruptions resulted in an abrupt increase in the flow rate (discussed further in the Supporting Information, Section 1.3.5.2).

### 2.5 Operator Data Collection and Reporting

We use the multistage unblinding and data reporting procedures described in Rutherford et al.^3^ In stage 1 of data reporting, all operators submit fully blinded quantification estimates. These stage 1 data are, therefore, most representative of real-world measurement conditions. In stage 2, we provided operators with 10 m wind data collected onsite. All operators could then reanalyze the results and submit modified quantification estimates using the measured wind data. The difference between stage 1 and stage 2 results therefore represents a potential improvement from having access to real-time ground wind data. Finally, in stage 3, we provided operators with metered methane release rates for approximately half of their measurements, which could be used to inform a final submission based on an updated algorithm. Stage 3 results thus represent potential improvements possible with algorithm tuning. Details on data selection criteria for stage 3 are included in the Supporting Information, Section 1.3.6. All operators were provided the opportunity to participate in all three stages of analysis, although only Carbon Mapper, GHGSat-AV, and Insight M chose to do so. MethaneAIR faced personnel and time limitations. Scientific Aviation collects wind measurements onboard aircraft instrumentation^12^ making stage 2 irrelevant, and the small sample size limited the value of stage 3. Also, note that Insight M data are the combined results from two measurement units, and MethaneAIR reports the average of two different analysis methods (both discussed in detail in the Supporting Information, Section 2.3.).

## 3 Results

**Figure 2:**
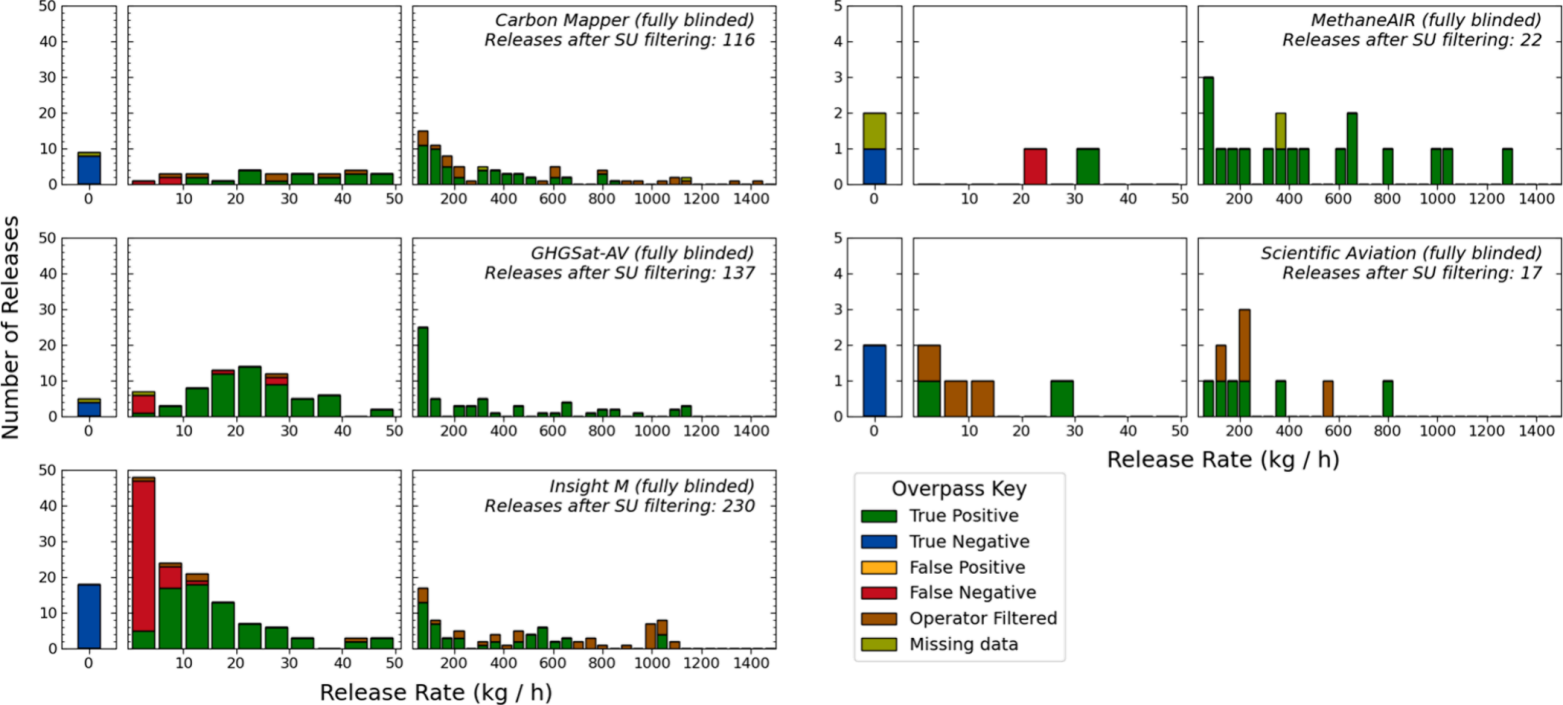
Distribution of releases for each aircraft tested; colors indicate results classification: true positive, true negative, false positive (no teams reported false positives), false negative, operator filtered (measurements for which the operator determined quantification was not possible), and missing data. Note that the three plots on the left have a different *y*-axis than the two on the right. For all operators, we conducted releases ranging from 0 to 1,500 kg CH_4_/h. Figures do not include measurements filtered by Stanford (SU), e.g., due to insufficient wind transport.

Over the aircraft testing period, October 10 through November 11, 2022, we conducted 711 measurements with the five different aircraft operators. Of these measurements, 189 were removed by Stanford for failing to meet quality control criteria designed to ensure clean conditions, given real-time winds. Stanford exclusion criteria were finalized and applied before Stanford personnel viewed any operator results. The remaining 522 releases are included in Figure 2. Of total measurements conducted, 63 (8.9%) were intentional zero releases (0 kg/h) to serve as negative controls. There were a small number of times (seven total) when the aircraft flew over the field site, but no associated measurement was submitted with the operator report (due to some measurement or processing error). These points are classified as “missing data” in Figure 2 (additional details in the Supporting Information, Section 1.3.3 and Table S13).

Table 2 summarizes the operator-specific parameters for the measurements conducted in this study. For reported metered flow rates, we use significant figures based on level of precision of the measurement and calculated uncertainty. All teams correctly categorized negative controls as 0 kg(CH_4_)/h, with no teams producing false positives. Additionally, we find no false negatives larger than 30 kg(CH_4_)/h, and Insight M, GHGSat, and Scientific Aviation quantified plumes smaller than 4 kg(CH_4_)/h. Carbon Mapper, GHGSat-AV, and Kairos consistently quantify releases above 10 kg(CH_4_)/h. For Insight M, 107 of 191 valid measurements were less than 15 kg(CH_4_)/h, providing the greatest characterization of minimum detection across all operators. MethaneAIR and Scientific Aviation had a smaller sample size overall and particularly for releases under 50 kg(CH_4_)/h. GHGSat-AV had three false negatives above 5 kg(CH_4_)/h (16.78 [16.67, 16.81], 29.01 [28.83, 29.18], and 29.17 [28.99, 29.35] kg(CH_4_)/h), which make up 8% of all measurements conducted in this range between 15 and 30 kg/h. Additionally, Carbon Mapper detected (but did not quantify) a release at 8.64 [8.45, 8.80] kg(CH_4_)/h.

**Table 2:** Summary of Reported Measurements, Data Filtering, and Key Results for Each Platform

	**Carbon Mapper**	**GHGSat-AV**	**Insight M**	**MethaneAIR**	**Scientific Aviation**
number of reported measurements	121	192	349	24	18
number of measurements filtered by Stanford	8	57	119	4	1
number of measurements filtered by operator^a^	31	1	39	0	7
no. of quantified measurements to pass all filtering	82	140	191	20	11
range of nonzero Stanford release volumes^b^	4.45 [4.30, 4.59] - 1,440 [1,370, 1,520] kg CH_4_/h	1.05 [1.02, 1.08]–1,140 [1,110, 1,180] kg CH_4_/h	0.64 [0.59, 0.69]–1,110 [1,050, 1,180] kg CH_4_/h	24.42 [24.31, 24.53]–1,290 [1,220, 1,360] kg CH_4_/h	3.77 [3.72, 3.83]–800 [780, 830] kg CH_4_/h
smallest quantified plume (kg CH_4_/h)	10.92 [10.78, 11.06] kg CH_4_/h	2.91 [2.86, 2.96] kg CH_4_/h	3.40 [3.35, 3.46] kg CH_4_/h	33.61 [33.27, 33.94] kg CH_4_/h	3.77 [3.71, 3.83] kg CH_4_/h
largest false negative (kg CH_4_/h)	6.61 [6.47, 6.76] kg CH_4_/h	29.17 [28.99, 29.35] kg CH_4_/h	10.47 [10.40, 10.53] kg CH_4_/h	24.42 [24.31, 24.53] kg CH_4_/h	no false negatives

a Operator filter applied only to measurements that pass Stanford filtering.

b Nonzero Stanford releases before operator filtering.

**Figure 3:**
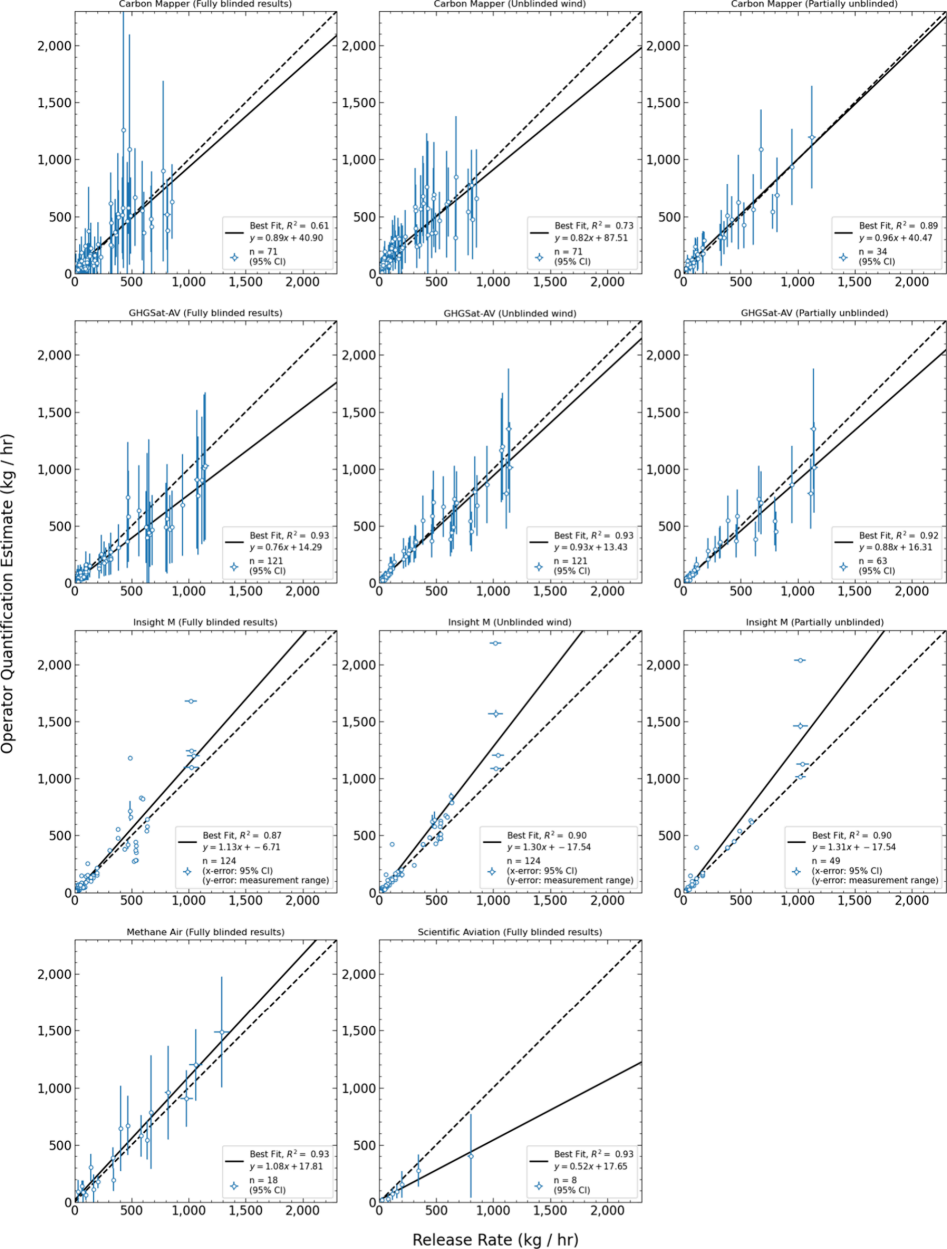
Quantification accuracy of the aircraft platforms. Metered release rate is on the *x*-axis with error bars representing 95% CI, often not visible due to low values. Operator reported quantification estimates are on the *y*-axis. The dashed line represents the *x* = *y* parity line. For all operators except Insight M, *y*-axis error bars represent operator reported uncertainty as 95% CI. Insight M does not report uncertainty, and *y*-error bars represent the variability in the two wing mounted measurement units flown during testing conditions.

In Figure 3, we assess quantification accuracy for all correctly identified nonzero releases (true positives). For each stage of unblinding, we compare the metered release rate in kg(CH_4_)/h (*x*-axis) with the reported estimate (*y*-axis). Carbon Mapper, GHGSat, and Insight M participated in the three stage unblinding process described above, and for these three operators, stage 1 results are in the left column, stage 2 in the middle column, and stage 3 in the right column. MethaneAIR and Scientific Aviation only participated in the first stage, submitting fully blinded results. Results for these two operators are in the bottom row.

For plots in Figure 3, we include all quantified nonzero measurements to determine the linear equation of best fit using ordinary least-squares (OLS) regression, as in Sherwin et al.^15^ OLS is appropriate here because of the much smaller *x*-axis errors than *y*-axis errors (e.g., metered emissions rate has high certainty). For all operators except Insight M, error bars on both the x- and *y*-axes represent the 95% confidence intervals (CI) of metered and reported results, respectively. Carbon Mapper, GHGSat-AV, and Scientific Aviation reported uncertainty using 1-sigma values, which we convert for consistency. MethaneAIR reported uncertainty in 95% CI. Insight M did not report uncertainty values for quantification estimates. For Insight M, each point represents the average of the two measurement units used for collecting data, which vertical error bars depicting reported values of individual units (analysis for each pod included in the Supporting Information, Section 2.3.2).

For fully blinded result submission (Stage 1), we requested operators submit using an analysis typical of standard operations. The four spectroscopy-based technologies submitted using the wind analysis products listed in Table 1. All three operators who submitted stage 2 estimates used Stanford-provided 10 m wind data. For stage 3 partially unblinded submissions, Figure 3 only includes the quantification estimates for releases that remained blinded, resulting in a smaller sample size. Carbon Mapper requested the ability to readd measurements they filtered in earlier stages as “poor quality” if unblinded information in later stages (wind data or unblinded measurements) increased confidence in quantification estimates (discussed more fully in the SI, Section S2.1.1.). Thus, quantification estimates for measurements not in stages 1 and 2 appear in the stage 3 parity figure.

Across all spectroscopy-based platforms, the linear regression slopes for fully blinded estimates (stage 1) range from 0.76 to 1.13, with *R*^2^ values ranging from 0.61 to 0.93. For Scientific Aviation, the sole operator to use the in situ measurement approach, we find a slope of 0.52 for all reported quantification estimates. However, the small sample size means the low estimate at 800 kg CH_4_/h has an outsized effect on the linear regression; removing this measurement from linear regression calculations increases slope of the best fit line from 0.52 to 0.82 (Figure S25). All operators except Insight M reported measurement estimates with associated uncertainty ranges. For Carbon Mapper, GHGSat-AV, and MethaneAIR, over 80% of all fully blinded estimates have 95% confidence intervals that encompass the metered release rate. For all platforms, the percentage of measurements for stage 1 that fall within 50% of the metered release rates ranges from 68% to 88% (Table 3).

**Table 3:** Key Metrics for Quantification Performance Across All Platforms

	unblinding stage	quantified nonzero measurements	slope (*R*^2^)^a^	operator estimates with 95% CI encompassing metered value (%)^b^	operator estimates within 50% of the metered value (%)
Carbon Mapper	stage 1	71	0.89 (0.61)	89%	68%
	stage 2	71	0.82 (0.73)	76%	44%
	stage 3	34	0.96 (0.89)	71%	62%
GHGSat-AV	stage 1	121	0.76 (0.93)	93%	80%
	stage 2	121	0.93 (0.93)	84%	88%
	stage 3	63	0.88 (0.92)	81%	89%
Insight M	stage 1	124	1.13 (0.87)	NA	73%
	stage 2	124	1.30 (0.90)	NA	93%
	stage 3	49	1.31 (0.90)	NA	94%
MethaneAIR	stage 1	18	1.08 (0.93)	83%	78%
Scientific Aviation	stage 1	8	0.52 (0.93)	63%	88%

a Slope and *R*^2^ are associated with the linear equation of best for using ordinal least squares.

b Insight M does not report uncertainty associated with measurements, and thus we cannot calculate the percentage of measurements with confidence intervals that contain the metered gas release rate.

Results from stage 2 and stage 3 demonstrate the potential benefits of improved wind data and iterative single-blind testing, respectively. When provided ground truth wind data, Carbon Mapper reported estimates with reduced scatter (slope = 0.82, *R*^2^ = 0.73) and greater certainties: average error bar length decreased from 200 to 170 kg CH_4_/h, which is reflected in the decrease in the percentage of measurements with uncertainty ranges that encompass the metered flow rate. However, both strength of fit and accuracy are highest in Carbon Mapper’s stage 3 results (slope = 0.96, *R*^2^ = 0.89), where a subset of unblinded measurements informed analysis and quantification estimates. Note that in this stage, Carbon Mapper chose to include two measurements previously removed by their own internal quality control.

For GHGSat-AV, ground truth wind data improved slope alignment with the parity line and decreased the reported uncertainty. The best-fit slope increased from 0.76 to 0.93, and the average reported uncertainty in stage 2 is 60% that of stage 1 (range is 10–110%). Fewer quantification estimates have error bars crossing the parity line, reflecting this narrowing of the confidence intervals. While GHGSat-AV participated in stage 3, they chose to make no adjustments to their stage 2 submission after viewing unblinded data–thereby our analysis of stage 3 results is simply a subset of stage 2 results.

Insight M demonstrated consistent performance in terms of best-fit slope and *R*^2^ values across all three stages. In stages 2 and 3, we note substantial improvement in the percentage of measurements that fall within 50% of the metered release rate, increasing from 73 to over 90%. This finding is associated with changes Insight M made to quantification estimates for lower release rates, which reflect a large portion of total measurements (as visible in Figure 2), an improvement not fully captured by comparing linear regression results alone.

For all spectroscopy-based technologies (Carbon Mapper, GHGSat-AV, Insight M, and MethaneAIR), percent error (depicted in Figures S20–S22) is greatest for measurements conducted at rates below 200 kg(CH_4_)/h. For Carbon Mapper, GHGSat-AV, and Insight M, absolute quantification error increases with increasing release rates, while percent error decreases. The magnitude of the quantification error does not appear to increase with increasing emission rates for MethaneAIR, although the sample size is limited. This result likely reflects the high sensitivity of the sensor to differences in CH_4_ enhancement, and the application of two quantification methods with complementary error characteristics. The small sample size for Scientific Aviation limits our ability to draw conclusions regarding trends in the error profile. Percent error for Scientific Aviation quantification estimates are within the range of those observed for fully blinded estimates by Carbon Mapper, GHGSat, and Insight M for similar release ranges. A small sample size means the low estimate at 800 kg/h has an outsized effect on the linear regression; removing this measurement from linear regression calculations increases slope of the best fit line from 0.52 to 0.82 (Figure S25). Additional testing is needed for a more complete picture of Scientific Aviation’s capabilities and error profile.

**Figure 4:**
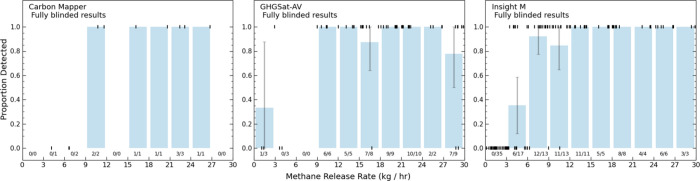
Detection capabilities below 30 kg(CH_4_)/h. Here, we show the probability of detection for releases that the operators quantified. Each release is represented by the vertical line at either *y* = 0 for releases not detected or *y* = 1 for detected releases, and releases are ordered along the *x*-axis based on volume. Blue bars represent the proportion of releases in each bin that were detected with error bars representing 95% confidence intervals, assuming a binomial distribution.

Figure 4 illustrates the fraction of releases detected below 30 kg(CH_4_)/h for Carbon Mapper, GHGSat-AV, and Insight M. MethaneAIR and Scientific Aviation are not included due to low sample size in this range. Characterizing lower detection limit was not a focus of Carbon Mapper measurements, hence, the smaller sample included in Figure 4. All operators consistently detected releases above 10 kg(CH_4_)/h. While we conducted far fewer releases below 10 kg(CH_4_)/h for GHGSat-AV, both Insight M and GHGSat-AV detected a small proportion of releases below 5 kg(CH_4_)/h. Additionally, GHGSat-AV missed 3 nonzero releases above 15 kg(CH_4_)/h. All operators detected all releases above 30 kg(CH_4_)/h.

## 4 Discussion

In this work, we evaluate the performance of five different aircraft-based methane sensing systems. Importantly, we use the same field site and standardized protocols, allowing for comparison across the platforms in this study and with testing in other studies. This is the first independent, single-blind test of Scientific Aviation. Of the four systems previously tested by Brandt-group researchers at Stanford, all demonstrated improved performance.^3,15,18^ Note that previous tests with Insight M were conducted at a higher flight altitude (900 m/3,000 feet above ground level). A key finding of this work is the substantial improvement achieved by platforms conducting repeated testing, demonstrating the overall technical maturity of this field over the last several years.

Carbon Mapper shows improved detection and quantification performance compared to results reported by Rutherford et al.^3^ Previously, Carbon Mapper flew flight lines shorter than typical, which their internal postfacto analysis suggests introduced low bias into quantification estimates.^20^ For results reported here, Carbon Mapper flew 20 km flight lines, but other technical configurations remained similar to those of the earlier test. The best-fit slope for fully blinded quantification estimates increased from 0.33 (*R*^2^ = 0.35) in the earlier study to 0.89 (*R*^2^ = 0.61). In the previous study, Carbon Mapper also showed a trend of overestimating lower emissions and underestimating larger emissions, a trend not observed in these results.

GHGSat-AV fully blinded results in this study show reduced scatter compared to previous testing.^3^*R*^2^ increased from 0.38 to 0.93, indicating a much closer agreement with a linear fit. While the best-fit slope deviates more from the parity line (current study slope = 0.76, previous study slope = 1.0), reduced scatter is indicative of overall improved performance: in Rutherford et al., 2023, GHGSat-AV at times underestimated releases greater than 1,000 kg/h by a factor of 2 more,^3^ while our results show no evidence of biased quantification for large releases.

GHGSat-AV also demonstrated improved detection capabilities. In Rutherford et al., they did not detect any releases below 10 kg(CH_4_)/h, and missed over half of releases between 10 and 15 kg(CH_4_)/h.^3^ Here, GHGSat-AV detected one release below 5 kg(CH_4_)/h, and all releases between 5 and 15 kg(CH_4_)/h. In both studies, GHGSat-AV missed a small number of releases above 25 kg(CH_4_)/h. In Rutherford et al., GHGSat-AV missed 2 of 42 releases between 25 and 35 kg(CH_4_)/h (release rates: 31.0 and 32.4 kg(CH_4_)/h).^3^ In this study, GHSat-AV missed 2 out of 16 releases between 25 and 35 kg(CH_4_)/h, both ∼29 kg(CH_4_)/h.

Insight M maintained quantification performance while improving lower detection limit.^15^ In Sherwin et al., Insight M had a best-fit slope of 1.19 (with Dark Sky wind reanalysis) compared to our result of 1.13. However, the flight configuration here shows a decrease in the detection threshold. Previously, Insight M was able to correctly identify all wind-normalized release rates 15 kgh/mps or larger.^15^ When normalizing our results by windspeed, we find Insight M identifies all releases above 5 kgh/mps (see Figure S28). Sherwin et al. find a standard deviation of percent error for all releases above the full detection limit (41.76 kg(CH_4_)/h) to be 30–40%.^15^ Using the same lower limit for comparison purposes, we find a similar standard deviation for percent error of 43%. However, we note that the tested configuration with two wing-mounted units may not be representative of field performance and different test configurations limit direct comparison.

MethaneAIR previously conducted controlled releases in collaboration with Stanford using a collaborative technology-validation design, as reported in Chulakadabba et al.^18^ Quantification accuracy is similar to the previous study, but with reduced scatter (current study slope = 1.08 with *R*^2^ = 0.93; previous study OLS slope = 0.85 and York slope = 0.96, *R*^2^ = 0.83).^18^ However, results are not directly comparable, as the previous study reports quantification estimates using the mIME method, while MethaneAIR reported the average of two methods in the current study (results for individual methods in the Supporting Information, Section 2.3.4).

Conley et al. report two natural gas controlled release measurements for Scientific Aviation, although these were not part of a single-blind study.^12^ Both these releases were at rates of 14 kg(CH_4_)/h, smaller than all but one of the nonzero releases quantified by Scientific Aviation in the current study.

As a whole, this work also underscores the important role single-blind testing can play in technology development. Using other studies as a point of comparison for this work demonstrates the rapid technical advances achieved in a relatively short period of time. The value of repeated real-world testing is further underscored by the improvements achieved by Carbon Mapper in stage 3 of analysis, which allowed their team to adapt analysis based on partial data unblinding and test this new approach on a smaller blinded data set.

The present study has several important limitations. Providing participants with a known source location likely artificially inflates the detection performance. However, it is unlikely to affect the quantification capabilities. We also selected our testing location to minimize confounding sources and provide a uniform, dry terrain as the background. Field measurements will often occur over complex terrains with multiple confounding sources within the measurement range. Thus, our findings represent best-case performance, and we anticipate a decline in capabilities with the increased scene complexity present in real-world oil and gas facilities. While future testing can evaluate aircraft measurement under increasingly heterogeneous and real-world conditions, this work represents a necessary first step in establishing baseline performance. Furthermore, weather conditions during testing were conducive to measurement with limited cloud cover. Cloudy conditions add challenges for spectroscopy-based detection and quantification.

This work provides a comprehensive overview of major methane-sensing aircraft technologies. While we did not test Bridger Photonics, this company has been extensively tested elsewhere.^3,14,19^ We evaluate the state-of-the-art for all systems tested, demonstrating the ability of aircraft-based technologies to produce estimates with limited bias and within reasonable error. Our results also underscore the importance of controlled-release testing to allow technology developers to fine-tune their systems. Both Carbon Mapper and GHGSat-AV demonstrated substantial performance advances compared to previous tests,^3^ and the multistage unblinding within this study allowed Carbon Mapper to rapidly iterate and hone their quantification algorithm.

This study demonstrates that aircraft-based methane sensing is posed for an increasingly important role in climate change mitigation efforts and improving accuracy of the global methane budget. The approach outlined here can be used as technologies continue to mature and new methods develop, ensuring that high quality, accurate measurements underpin environmental regulation and enforcement.

## Data Availability

All data and code required to reproduce the figures and analysis in this paper are publicly available on GitHub: https://github.com/sahar-elabbadi/SU-Controlled-Releases-2022

## Supporting Information Available

Detailed methodology (field setup, metering system, Stanford quality control, data processing, descriptions of technologies tested), daily average windspeed, error and best-fit residual plots for quantification estimates, individual pod analysis and wind normalized detection results for Insight M, individual method results for MethaneAIR, and daily plume release rates for all testing days (PDF)
