# Supplementary material for: Technological Maturity of Aircraft-Based Methane Sensing for Greenhouse Gas Mitigation
Source: Environ Sci Technol. 2024 May 17;58(22):9591–600. doi: 10.1021/acs.est.4c02439 (PMC11154951; doi:10.1021/acs.est.4c02439)
Supplement: Supplementary file 1 — es4c02439_si_001.pdf [file es4c02439_si_001.pdf]

Supporting Information

Technological maturity of aircraft-based methane sensing for greenhouse gas mitigation

Sahar H. El Abbadi[1, a, *], Zhenlin Chen[1], Philippine M. Burdeau[1], Jeffrey S. Rutherford[1, b], Yuanlei Chen[1], Zhan Zhang[1], Evan D. Sherwin[1, a], Adam R. Brandt[1]

[1] Department of Energy Science & Engineering, Stanford University, Stanford, California 94305, United States
[a] Present affiliation: Lawrence Berkeley National Laboratory, 1 Cyclotron Road, Berkeley, California 94720, United States
[b] Present affiliation: Highwood Emissions Management, Calgary, Alberta T2P 2V1, Canada

*Corresponding author: Sahar H. El Abbadi, elabbadi@lbl.gov

Summary:

- 62 pages
- 29 figures
- 18 tables

Table of Contents

1 *Supplemental Methods* 5

1.1 Experimental Field Setup 5
- 1.1.1 Compressed natural gas trailers 6
- 1.1.2 Pressure regulation trailer 6
- 1.1.3 Gas metering trailer 9
- 1.1.4 3-D ultrasonic anemometer 14
- 1.1.5 2-D ultrasonic anemometer 16

1.2 Data processing for raw meter data 16
- 1.2.1 Metering trailer data log 16
- 1.2.2 Coriolis meter historical files 16
- 1.2.3 Gas compositional analysis 19

1.3 Aircraft testing 22
- 1.3.1 Field testing conditions 22
- 1.3.2 Description of Technologies Tested 24
- 1.3.3 Data reporting and unblinding 27
- 1.3.4 Data processing for aircraft testing 28
- 1.3.5 Stanford data quality control 31
- 1.3.6 Stage 3 data selection 34

2 *Supplementary Results* 36

2.1 Daily average windspeed 36

2.2 Aircraft data reporting 37
- 2.2.1 Carbon Mapper 37
- 2.2.2 Insight M 38
- 2.2.3 MethaneAIR 38
- 2.2.4 Scientific Aviation 38

2.3 Additional analysis of operator-reported results 39
- 2.3.1 Quantification Accuracy: error profile and best-fit residuals 39
- 2.3.2 Insight M individual pod analysis 46
- 2.3.3 Insight M wind normalized probability of detection 47
- 2.3.4 MethaneAIR Results for DI and mIME Methods 48

3 *References for Supplemental Material* 49

4 *Appendix* 50

4.1 Daily plume release definition plots 50

4.2 Daily Release Rates 55
- 4.2.1 Carbon Mapper 56
- 4.2.2 GHGSat-AV 57
- 4.2.3 Insight M 59
- 4.2.4 MethaneAIR 60
- 4.2.5 Scientific Aviation 61

List of Figures

Figure S1: Overhead view of Stanford field site 5
Figure S2 RT-30 pressure regulation trailer. 7
Figure S3 Pressure regulation trailer. 8
Figure S4 Aerial photograph of gas metering trailer 9
Figure S5 Schematic of Quadratherm measurement apparatus and gas metering trailer....10
Figure S6 Sample flow rates with automated vs manual flow control. 12
Figure S7 Quadratherm attachment apparatus. 13
Figure S8 Orientation of CSAT 3B 15
Figure S9 Data processing flow chart 17
Figure S10 Sample Carbon Mapper plume images....25
Figure S11 Sample MethaneAIR plume images....26
Figure S12: Results of plume identification algorithm 29
Figure S13: Results of plume detection algorithm 29
Figure S14 Flow chart for release categorization algorithm....31
Figure S15 Sample releases that pass and fail Stanford's quality control criteria 33
Figure S16: Release for Scientific Aviation filtered by Stanford 34
Figure S17 Cumulative probability distribution each operator 36
Figure S18: Quantification estimates for Stage 1 and Stage 3 results for Stage 3 participants....39
Figure S19: Absolute quantification error ($kg(CH_4)/hr$) 40
Figure S20: Percent quantification error 41
Figure S21 Percent quantification error for releases under 50 kg / hr....42
Figure S22 Percent quantification error for measurements greater than 50 kg / hr 43
Figure S23: Residuals for best fit linear regression 44
Figure S24 Percent error of residuals for linear best fit 45
Figure S25 Scientific Aviation quantification plot excluding points release volumes greater than 800 kg CH_4 / hr....46
Figure S26 Quantification accuracy for Insight M individual pods LS23 and LS25 47
Figure S27 Insight M individual pods LS23 and LS25 probability of detection 47
Figure S28 Insight M probability of detection using wind normalized methane flow rate 48
Figure S29: MethaneAIR quantification results for mIME and DI methods 49

List of Tables

Table S1 Compressed natural gas trailer specifications. 6
Table S2: Full model number of each Coriolis meter....11
Table S3 Sizing and specifications for Emerson MicroMotion Coriolis meters....11
Table S4 Dates indicating usage of tall vs short release stacks 14
Table S5 Summary of timestamp adjustments to flowskid data log 17
Table S6 Meter accuracy threshold values. 18
Table S7 Summary of gaps in Coriolis meter data across all days of testing. 18
Table S8 Gas composition for each truck refill 20
Table S9 Gas Refill schedule 20
Table S10 Gas composition summary....21

Table S11: Coordinated release schedule for Scientific Aviation testing 23
Table S12: Summary of aircraft deviations from typical flight patterns......................................23
Table S13 Total number of missing measurements by operator...28
Table S14 Maximum allowable deviation in meter reading for defining individual releases.......30
Table S15 Distance threshold for each aircraft operator 32
Table S16: Summary of quality control comparison imaging technologies................................33
Table S17: Daily average windspeeds..37
Table S18: Operator data reporting timeline 37

1 Supplemental Methods

1.1 Experimental Field Setup

We conducted controlled releases from October 10th, 2022 through November 30th, 2022 near Casa Grande, Arizona at coordinates [32.8218489, -111.7857599]. We evaluated aircraft, satellite, drone, and ground-based technologies. Natural gas trailers and pressure regulation trailers were provided by Rawhide Leasing (https://www.rawhideleasing.com/), and Rawhide personnel operated this equipment (Mike Brandon, Walt Godsil, and S.M.). The gas metering trailer was designed by the Stanford team in collaboration with Volta Fabrication, who constructed the trailer. The Stanford team controlled gas flow rates using a WiFi-enabled laptop connected the flow control system on the metering trailer. Gas was released from two stacks, each with 6-inch diameter and release heights of 24 and 10 feet.

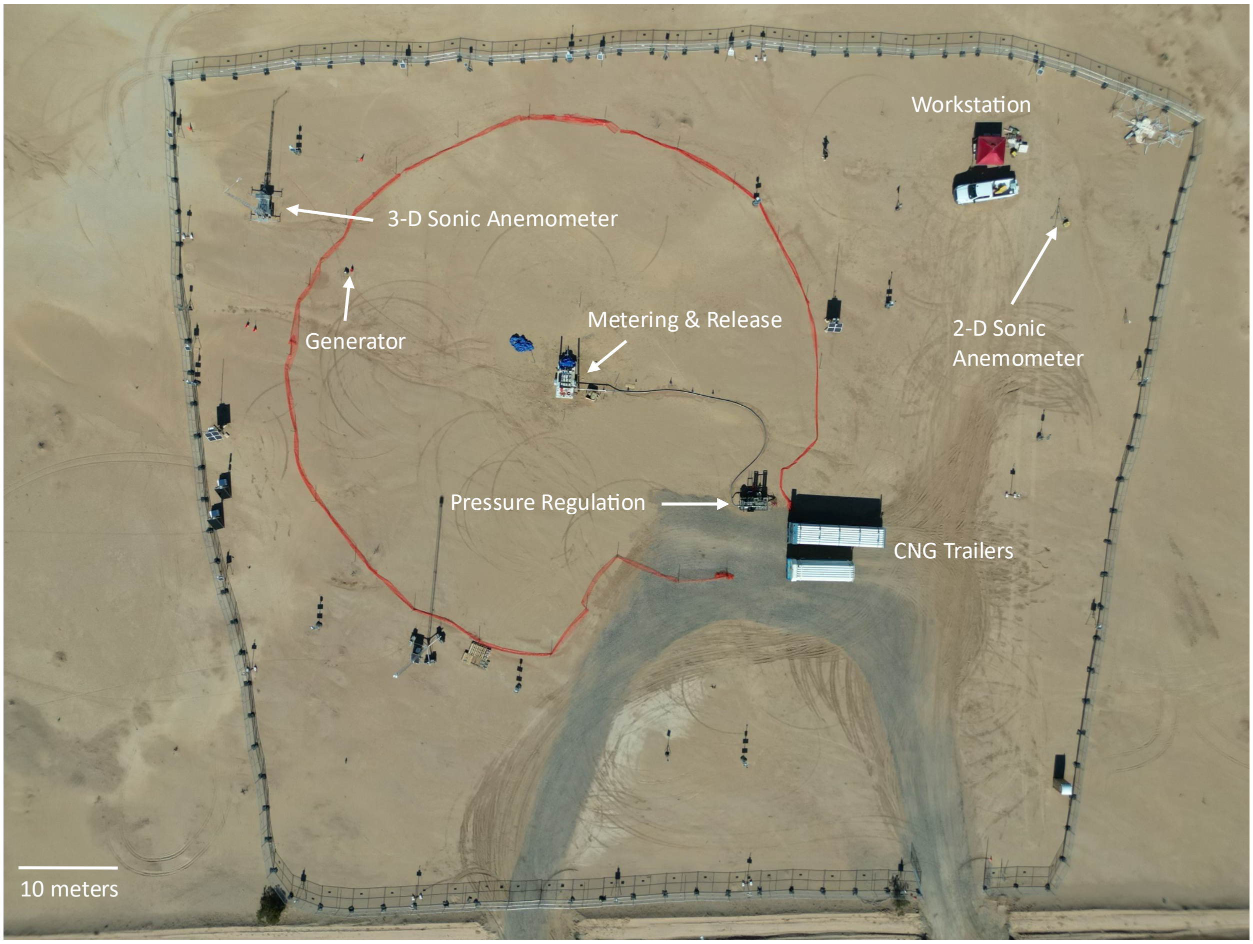

Figure S1: Overhead view of Stanford field site, with key components labelled. Also visible but not labelled here are individual ground sensors deployed for the duration of the experiment.

The testing configuration included the following key components, also depicted in Figure S1, described below in full:

1. Two compressed natural gas trailers
2. Pressure regulation trailer

3. Flow metering trailer fitted with three Emerson Micromotion Coriolis meters measuring gas flow rate in kg(CH_4)/hr. Two release stacks allow for vertical gas release at 7.3 meters (24-feet) and 3.0 meters (10-feet) above the ground
4. Three-dimensional sonic anemometer (Campbell Scientific, CSAT 3B) mounted at 10-meter height on an aluminum trailer tower (Aluma Towers)
5. Two-dimensional ultra-sonic anemometer, mounted at 2 meters
6. Workstation with computers for controlling flow rates and logging data, located over 45 meters (150 feet) from all gas flow equipment (49 meters (160 feet) from metering trailer, 45 meters (150 feet) from Rawhide equipment).
7. Infrared camera (FLIR GF320) focused on stack and used for real-time plume observations and recording

1.1.1 Compressed natural gas trailers

As previously described, we used compressed natural gas (CNG) as the source of methane for controlled releases (Ravikumar et al., 2019; Sherwin, Chen et al., 2021; Rutherford et al., 2022). CNG was purchased from local filling stations and stored onsite in two contracted CNG storage trailers. Capacity of CNG trailers is described in Table S1. Pressure in the CNG trailers ranged from 3.5 – 17.3 MPa (500 psig to 2500 psig), varying with ambient temperature and gas fill level.

Table S1 Compressed natural gas trailer specifications. Trailer IDs are assigned by Rawhide Leasing. Water volume in cubic feet refers to the total volume of water that can be held in the tank. Full capacity and working capacity refer to gas capacity at max pressure. Working volume accounts for the pressure differential needed to maintain gas delivery to the pressure regulation trailer.

Trailer	Water Volume (ft^3)	Max Pressure	Full Capacity (Mscf at max pressure)	Working Capacity (Mscf)
911-49	19.81 m^3 (699.5 ft^3)	16.6 MPa (2400 psig)	106	90
911-2	9.63 m^3 (342 ft^3)	17.3 MPa (2500 psig)	76	63

1.1.2 Pressure regulation trailer

When releasing gas, one or both of the CNG trailers is connected to a pressure regulation trailer (Rawhide Leasing, RT-30), which reduces pressure from that of the CNG trailer to the pressure rating of the gas metering trailer. Gas is transferred from trailers to the pressure regulation trailer using 13mm Parflex CNG hose, rated to withstand 34.5 MPa (5,000 psi). Depending on the amount of gas remaining in the CNG trailer, the inlet pressure to pressure regulation trailer changes. Detailed descriptions of the pressure regulators on the RT-30 are provided below. Gas leaves the pressure regulation trailer at 1.14 - 1.48 MPa (150 – 200 psig), and is delivered via a hose to the gas metering trailer.

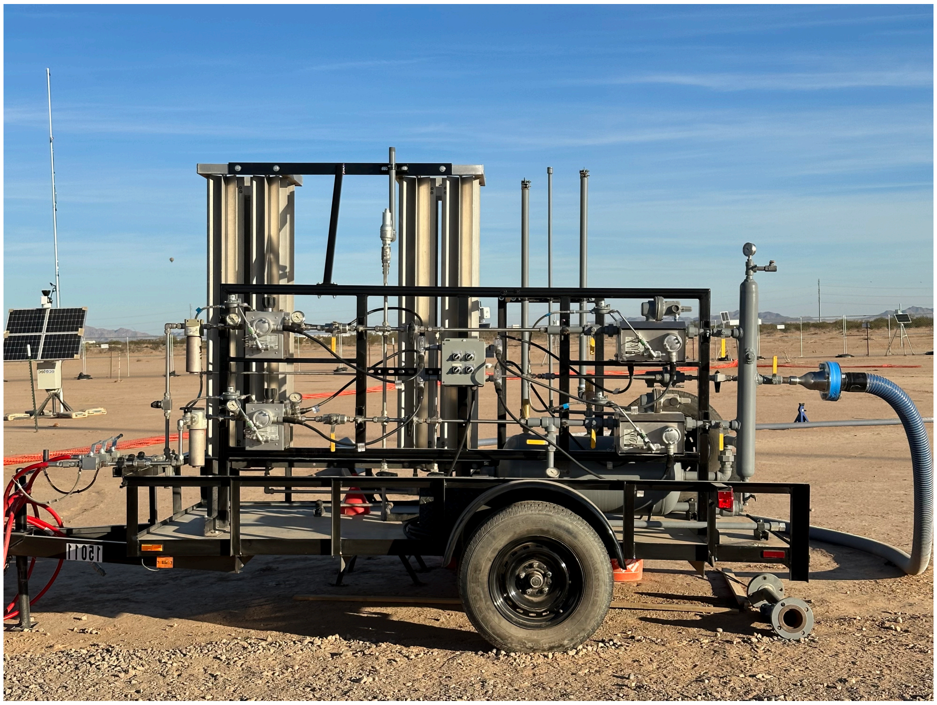

Figure S2 RT-30 pressure regulation trailer. Gas is delivered from CNG trailers via the red hoses on the left, and exits through the blue hose on the right.

Photograph and schematic of the regulation trailer are depicted in Figures S2 and S3. After entering the trailer inlet, gas is delivered to either one or both of two parallel pressure regulation lines. Each line is fitted with a microglass 6-micron fuel filter (3B Filters Inc., Model A8579-V6MD) followed by two pressure regulation units: a stainless steel Tescom pressure regulator (Model number 44-1325-2122-011) followed in-series by a Fisher pressure regulator (initially model number 627, then model number 627H; discussed further below).

Tescom regulators, rated for inlet pressures up to 31.13 MPa (4500 psig), decrease pressure from the level of the CNG trailer to 2.86 MPa (400 psig). The inlet pressure to the Tescom regulators changes with the amount of gas remaining in the CNG trailer, and thus the pressure drop across the regulator changes. This affects the cooling generated during gas expansion (discussed below). Next follows the Fisher pressure regulators, rated for inlet pressures of 700 psig, further stepping down the pressure to 1.14 – 1.48 MPa (150 - 200 psig). Initially, RT-30 was fitted with Fisher 627 pressure regulators, the maximum outlet pressure of which is 1.14 MPa (150 psig). On October 22, 2022, Rawhide personnel replaced the Fisher 627 with model 627H, enabling an outlet pressure of 1.48 MPa (200 psig). After leaving the Fisher pressure regulators, gas flows from the two pressure regulation lines into a multi-port manifold fitted with a pressure gauge to measure final outlet pressure from the regulation trailer.

Each pressure regulator is fitted with a catalytic heater (Tescom heaters: CATCO 90-66S1G-40; Fisher heaters: CATCO 90-612S1G-40), as depicted in Figure S3. Heaters are applied to partially compensate for the Joule-Thompson temperature drop resulting from the increase in gas pressure. Throughout the experiment, these catalytic heaters were used for gas flow rates exceeding ~250 kg/h for over 15 minutes. Gas from the multi-port manifold is used to power the

catalytic heaters. An additional Fisher pressure regulator reduces gas pressure from the manifold level (1.48 MPa or 200 psig) to 0.14 MPa (5 psig) before delivery to the heaters (not depicted in Figure S3).

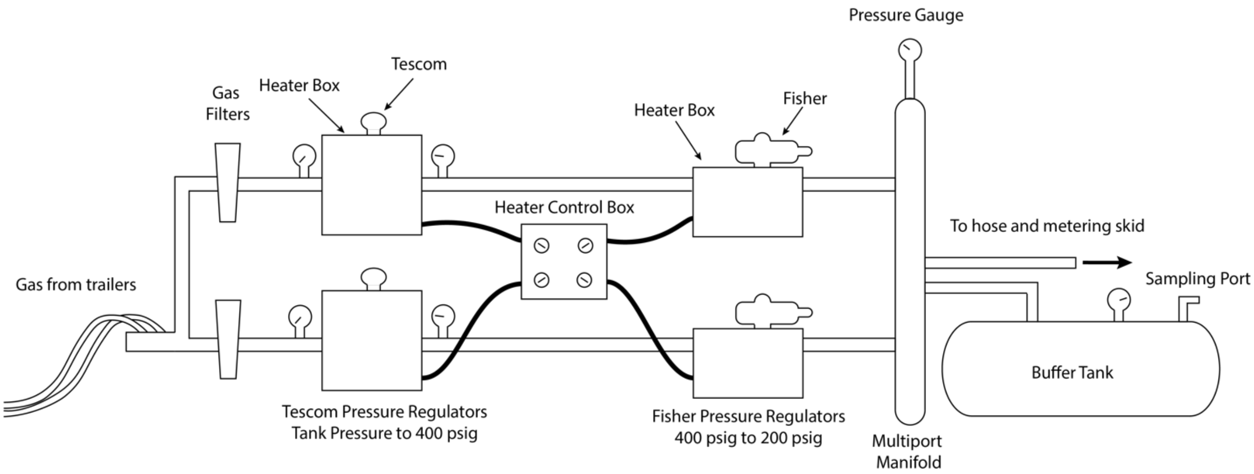

Figure S3 Pressure regulation trailer. Gas enters from the CNG trailers via red hoses on the left of the image, and passes through either one or both of two pressure regulation lines. Pressure is first dropped through the Tescoms pressure regulators to 2.86 MPa (400 psig) in the Fisher pressure regulators. A pressure gauge measures final outlet pressure before gas is delivered to a 3-inch hose which connects to the metering trailer.

The multi-port manifold on the pressure regulation trailer also connects to a buffer tank fitted with a pressure gauge and gas sampling port. The buffer tank is rated to withstand pressures of up to 1.48 MPa (200 psig) and has a safety valve set for 1.31 MPa (175 psig). To collect gas samples for analysis, a gas line connecting the manifold to the buffer tank is opened and pressure is allowed to reach 0.27 MPa (25 psig) within the buffer tank. Laboratory-supplied collection canisters are connected to the sampling port, the line to the port is opened, and gas flows from the tank into the collection canister. Details of gas sampling are described in further detail below (see Section S1.2.3 for further discussion of gas composition).

The pressure regulation trailer also includes the following equipment not depicted in Figure S3: Safety pressure release valves in case of failure in the pressure regulators; safety release valves on the buffer tank; and ambient air heaters (not used during the experiment). Safety pressure release valves on the pipe connecting the Tescoms to the Fishers are set for 6.65 MPa (950 psig).

After successive drops in pressure in the RT-30, gas is delivered to the gas metering trailer. The gas metering trailer was designed for inlet gas pressure of 1.14 MPa (150 psig), with all equipment rated for 1.48 MPa (200 psig). To achieve the desired inlet pressure, the metering trailer was originally intended to be coupled with the RT-60 pressure regulation trailer, not the RT-30. However, due to supply chain delays, RT-60 construction was not complete in time for testing. While pressure regulators in the RT-30 can drop gas pressure to 1.48 MPa (200 psig), this pressure is not maintained due to a constriction at the point where the RT-30 outlet connects to the hose that transports gas to the metering trailer. This meant that gas at the inlet of the metering trailer was typically lower than 1.14 MPa (150 psig) for large release volumes, despite the pressure gauge in the RT-30 multi-port manifold reading 1.48 MPa (200 psig). Pressure limitations impacted the maximum flow rate of the metering trailer. While designed to support gas releases up to 2,000 kg gas / hr, the maximum release volume achieved during throughout

the duration of this experiment was ~1,600 kg gas / hr. At the highest flow rates, the pressure drop in the system becomes large and flow becomes erratic.

1.1.3 Gas metering trailer

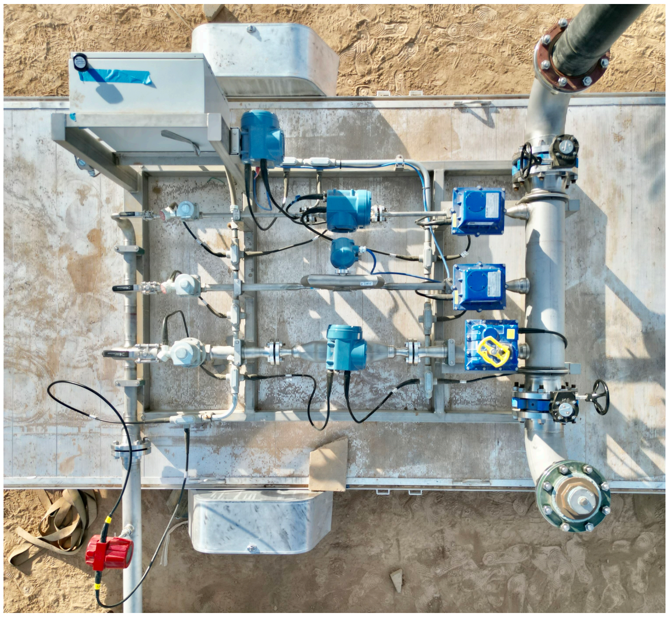

Figure S4 Aerial photograph of gas metering trailer

Gas from the pressure regulation trailer is transported to the gas metering trailer (photograph in Figure S4), consisting of three flow paths each fitted with a Coriolis gas flow mater. The Stanford team controlled the desired flow rate, diverting gas through one of the three flow paths, before it is released through one of two vertical stacks. In this section, we describe the metering and flow control mechanisms in detail.

1.1.3.1 Gas metering and flow control

A 7.62-cm (3-inch) wire-spring reinforced hose (pressure rating 200 psig) transports gas from the pressure regulation trailer to the gas metering trailer, as depicted in the schematic in Figure S5. Gas first passes a Quadratherm thermal mass flow insertion meter (see additional details below), before being diverted to one of three parallel lines, each line is fitted with an Emerson MicroMotion Coriolis meter (https://www.emerson.com/en-us/automation/micro-motion). All pipes in the Quadratherm measurement apparatus and the gas metering trailer are Schedule 10 stainless steel.

Model identification numbers, serial numbers, and calibration dates for each Coriolis meter are included in Table S2, and flow ranges for each meter are included in Table S3. The max flow range for each meter is based on the maximum recommended gas velocity of 140 m/s through the sensor (personal communication from Hector Rodriguez of Micro Motion to Jeff Rutherford on November 12, 2021). Due to supply chain delays, all testing prior to October 24th was conducted using the medium-diameter CMF050M meter only; subsequent tests used all three Coriolis meters. We attempted to conduct large satellite-coincident releases using the

Quadratherm 640i, but these measurements were discarded due to system malfunction (discussed below).

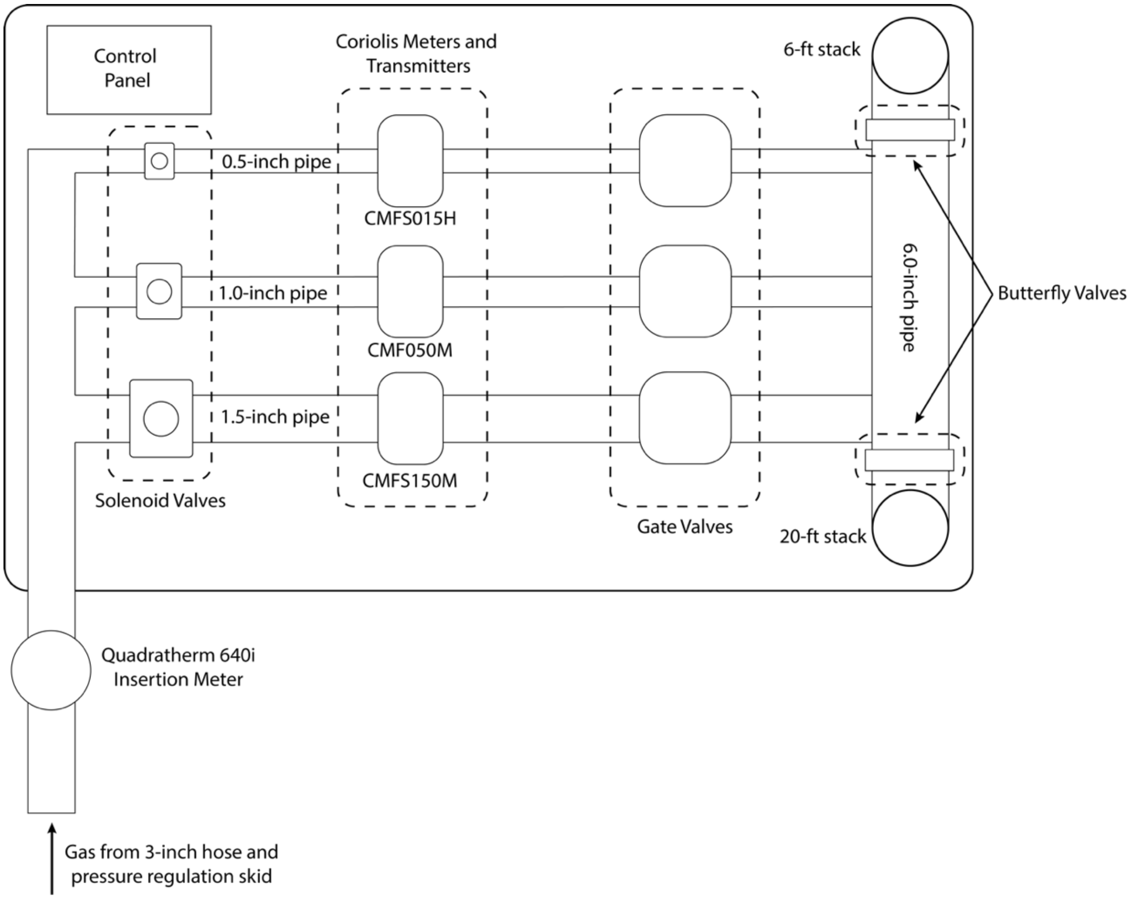

Figure S5 Schematic of Quadratherm measurement apparatus and gas metering trailer (not to scale). Gas flows past the Quadratherm insertion meter, before being diverted through one of three parallel lines, each fitted with a Micromotion Coriolis meter. Gas then enters a 15.24 cm (6-inch) pipe connected to two release stacks. Stacks are 6.1 meters (6 feet) and 1.8 meters (20 feet) long, releasing gas at 3.0 meters (10 feet) and 7.3 meters (24 feet) above ground level, respectively.

The gas flow into each meter is controlled by a solenoid valve (Magnatrol Valve Corp, Models F42K37-GSW, F31K34-GSW, and F14K32-GSW on the 1 ½-inch, 1-inch, and ½-inch lines, respectively) which can either be in the fully open or fully closed position. When a given solenoid valve is open, gas flows through the corresponding pipe and Coriolis meter. Only one solenoid valve was opened at a given time. A downstream flow control gate valve (SVC Flow Controls, Model E400X2EC) is used to set the gas flow rate based on the extent to which it is opened, measured in percent. After passing through flow control valves, gas then enters a 6-inch pipe fitted with two 6-inch butterfly valves (SVF Flow Controls, SLB series) that control flow to the release stacks.

Each Coriolis meter on the metering trailer is equipped with a field mounted Micro Motion 5700 transmitter that converts the raw sensor data to a 4-20 milliamp (mA) signal (see Table S3). A wired connection delivers the mA output from each transmitter to the metering trailer's programmable logic controller (PLC). The mA output signal was transmitted with zero dampening applied to the flow reading, dampening effects will be applied in the subsequent data

analysis pipeline. The PLC (Horner Automation, HE-X5GN) is located in the control panel depicted on Figure S5. A WiFi adaptor transmits data to a laptop computer operated at the Stanford Work Station. All external wiring from flow meter transmitters, flow control valves, and solenoid valves use Class 1 Division 1 hazardous location approved, ruggedized, pre-manufactured Mineral Insulated cables that are fire resistant and waterproof. Cables provide power to all meters and valves (M.I Cable Company, Part Number 2/16/3/SB6-12/H) and transmit the 4-20 mA signal from the meters to the PLC (M.I Cable Company, Part Number 2/16/3/SB6-12/H-TD).

Table S2: Full model number of each Coriolis meter. A meter consists of a sensor, through which the gas flows, and its associated transmitter. We include model ID and serial numbers for each sensor / transmitter pair.

Meter (Model Abbreviation)	Sensor Model ID	Transmitter Model ID	Sensor Serial Number	Transmitter Serial Number	Calibration Date
Small (CMFS015H)	CMFS015H520NFA2ECZZ	5700I12AB2AZZXAAAZA_40102	12219231	12222349	October 14, 2022
Medium (CMF050M)	CMF050M319N2BAEZZZ	5700R12ABAAZZXAAZZZ_40102	21175085	12205694	September 21, 2021
Large (CMFS150M)	CMFS150M341NFA2EKZZ	5700I12AB2AZZXAAAZA_40102	12220939	12222533	October 18, 2022

Table S3 Sizing and specifications for Emerson MicroMotion Coriolis meters. Flow range represents the desired flow range for each meter, although rates outside the specified range are possible and require adjustments to meter uncertainty.

Meter	Meter Size	Connecting Pipe Size	Flow Range (kgh)	4-20 mA Output Range
Small	0.166 inch	0.5 inch	2 – 30 kgh	0 – 50 kgh
Medium	0.5 inch	1.0 inch	30 – 300 kgh	0 – 400 kgh
Large	1.5 inch	1.5 inch	300 – 2,000 kgh	0 – 3,000 kgh

Flow rates can be controlled using either an automated or manual control system, using a WiFi connected laptop. The automated feedback system uses a proportional-integral-derivative (PID) controller to adjust the flow control valve to achieve a desired set point, while the manual control system allows the Stanford team to set the degree to which the gate valve opens by specifying a desired percentage.

From October 10th – 20th, we used an automated feedback system for flow control. However, we observed flow fluctuations associated with overcorrections in the feedback system. While accuracy of measurement was not affected, flow rate was more variable (see Figure S6A). On October 20th we switched to manually controlling valve settings via the laptop interface. With this control mechanism, we set the gate valve position in order to achieve a desired flow rate. Figure S6B shows the reduction in fluctuations achieved by switching to this manual control system.

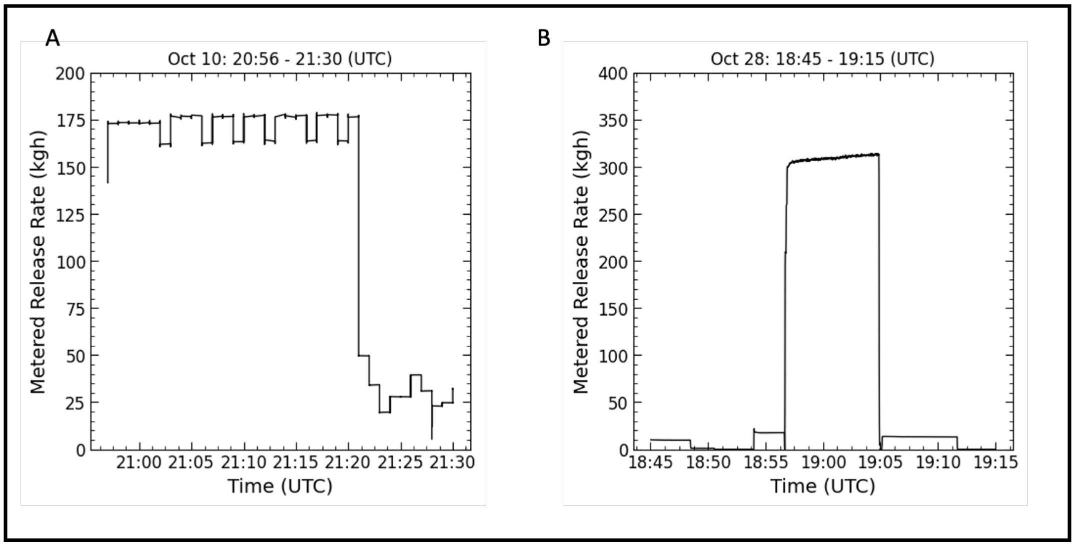

Figure S6 Sample flow rates with automated vs manual flow control.Note the y-axis differs in the two plots

Thermal mass flow metering

Our gas metering and release trailer included upstream thermal mass flow meters for comparison with the Emerson MicroMotion meters and for potential use prior to the arrival of the large meter (shipment delayed due to supply chain issues). The Sierra instruments are calibrated for a range of 250 to 1,180 kg/hr (when installed in a 3-inch pipe). Thus, we intended to use it alongside the Medium Coriolis meter to conduct higher volume releases. However, both meters are calibrated to conduct releases at 300 kg/hr. When we conducted releases at this range, we observed inconsistent discrepancies between the two meter readings, in which they were often offset from each other by 10 - 20%. Due to higher documented level of manufacturer-reported measurement certainty with the Coriolis meters, we opted to use only the Coriolis meters for all experimental data. However, here we provide documentation of the thermal mass flow meter configuration.

Prior to entering the metering trailer, gas passes through a 7.6 cm (3-inch) inlet pipe equipped for installing an insertion flow meter. For all testing, the spool included an installed Sierra Instruments Quadratherm 640i (Figure S7A), connected to the PLC and transmitting meter readings to the connected laptop computer via WiFi. During testing on October 10, 11th and 12th, gas was delivered through the Quadratherm 640i and subsequently through the CMF050M, at flow rates overlapping with the calibration range of the two meters. Due to observed inconsistencies in meter reading, we made several adjustments to the hose and Quadratherm metering configuration, and added installed an additional upstream Quadratherm 780i for cross comparison.

Throughout all testing, the Quadratherm 640i had an upstream straight run of pipe over 356 cm (140 inches) long, corresponding to >46 upstream diameters (see Figure S7A). This upstream pipe length exceeds the requirements reported in the Sierra Quadratherm manual, which recommends 40 upstream diameters of straight pipe after a flow control valve or two elbows in a different plane (Sierra Instruments, 2014). Downstream of the Quadratherm 640i were 32 cm (12.5 inches) of straight pipe before a 7.6 cm to 5.1 cm (3-inch to 2-inch) pipe flange that marks gas entering the gas metering trailer itself. This length corresponds to >4 downstream diameters,

exceeding the recommended 3 straight-pipe diameters recommended downstream of the Quadratherm 640i when downstream pipe size decreases by a factor of 4:1 (Sierra Instruments, 2014).

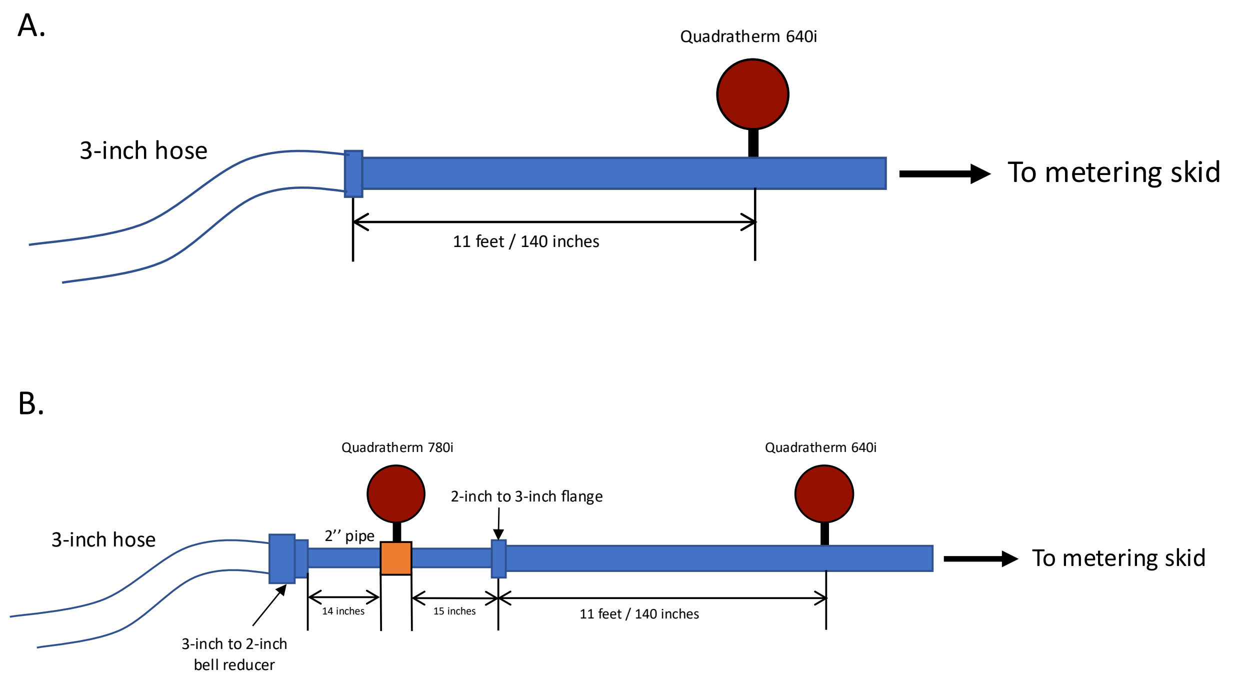

Figure S7 Quadratherm attachment apparatus. A. Upstream pipe connecting 3-inch hose to metering trailer with installed Quadratherm 640i. B. Pipe spool with Quadratherm 780i installed in series upstream of the Quadratherm 640i. The inline pipe attached to the Quadratherm contains the flow conditioning unit and is depicted in orange.

On October 13th, the 3-inch hose delivering gas to the Quadratherm 640i spool was straightened and elevated with car jacks to further reduce any potential upstream sources of turbulence to the gas flow. With this configuration, there were 14.5 feet between the Quadratherm 640i and the downward curve of the hose, and 35 feet before the hose curved laterally towards the pressure regulation trailer.

From October 17th through October 30th, an additional 2-inch pipe was installed with an inline Sierra Instruments Quadratherm 780i meter, provided by Insight M for measurement inter-comparison (Figure S7B). This meter was placed upstream of the 640i and allowed us to compare the reading from two Quadratherm meters in series. The 780i Quadratherm included a flow conditioning unit within the inline pipe manufactured and attached to the meter itself. The installed piping included 35.56 cm (14 inches) of upstream straight pipe and 38.1 cm (15 inches) of downstream straight pipe, corresponding to 7 and 7.5 upstream and downstream diameters, respectively. Upstream and downstream pipe lengths comply manufacturer's recommendations for this instrument and this piping configuration.

Ultimately, none of the data from Quadratherm thermal mass flow meters are used in generating final flow rate measurements for analysis. The calibrated uncertainty on the Coriolis meters is much smaller than the Quadratherm meters, and the empirical comparison between the two Quadratherm meters in series supports this fact. Therefore, the Quadratherm meters are treated as

"backup" meters only and were not required to be used in any testing. Intercomparison between the measurements of the Quadratherm meters and the CMF050M Coriolis meter may be the subject of future analysis.

1.1.3.3 Gas Release Stacks

The two gas release stacks are made of 6-inch diameter high-density polyethylene tubing, 6 meters (20 feet) and 1.8 meters (6 feet) long, attached to a rotating elbow joint. When stacks are in the vertical position, gas is released 7.3 meters (24 feet) and 3 meters (10 feet) above ground level, respectively. The rotating elbow assembly allows gas to also be released while the stacks are in a horizontal position, with the polyethylene tubing parallel to the ground. In this configuration, gas is released 0.9 meters (3 feet) above ground level. In this study, however, stacks were only used in the vertical position.

The metering trailer was designed for gas to flow through one open butterfly valve to the desired release stack, while the other butterfly valve shut gas flow to the stack not in use. We conducted initial testing using the 20-ft release stack. On Oct 26th, we observed gas slip from the short stack using the infrared camera (FLIR GF320). We reviewed our own internal infrared footage, as well as infrared footage continuously collected by a Kuva Systems unit installed on site for testing as part of the continuous monitoring testing program (Kuva Systems, 2023). We determined gas slip may have begun as early as October 20th. While we did not systematically evaluate when slip was occurring, we were able to visualize gas slip using high sensitivity mode on the FLIR camera at whole-system flow rates as low as 300 kg/hr. However, we were only able to consistently visualize slip during whole-system release rates exceeding 800 kg/hr. Because we only conducted releases at rates greater than 300 kg/hr after the large Coriolis meter arrived on October 20th, we have high certainty that meaningful gas slip did not occur before this date.

To prevent further gas leakage, on November 1st, we removed the short stack and sealed the pipe. On November 14th, we reinstalled the short stack, removing and sealing the tall stack. The timeline associated with stack leak is provided in Table S4. Methane slip occurred during testing of Insight M, and all continuous monitoring teams deployed for the relevant dates. Insight M reported observing leaks from both stacks in imaging, and we provided the information in Table S4 to all continuous monitoring teams.

Table S4 Dates indicating usage of tall vs short release stacks, and whether or not methane slip was observed.

**Date**	**Stack Usage and Slip**
October 10th – October 20th	Tall Stack, no slip
October 20th – October 30th	Tall stack, with slip
October 31st	Short stack, with tall stack slip
November 1st – November 14th	Tall stack, short stack removed (no slip)
November 14th – November 30th	Short stack, tall stack removed (no slip)

1.1.4 3-D ultrasonic anemometer

A three-dimensional (3-D) ultrasonic anemometer (Campbell Scientific, CSAT 3B) was mounted on a 10-m stainless steel trailer tower (Aluma Towers) at coordinates [32.8220109, -111.7861257], 33 meters (108 feet) from the release point. Coordinates are measured using an iPhone Google Maps pin drop. The anemometer was installed with the prongs oriented towards the direction of the dominant prevailing wind (NE), per manufacturer recommendations. The azimuth angle, or the angle of the anemometer orientation relative to Magnetic North, was 45°, as measured with a magnetic compass. This corresponds to a 35.3° angle relative to True North, assuming a declination value of 9.7° for Casa Grande, AZ (National Oceanic and Atmospheric Administration Geophysical Data Center, 2023). Orientation of the anemometer relative to True North and Magnetic North is depicted in Figure S8. Wind directionality is recorded in degrees relative to True North, and reported as a vector indicating the direction from which the wind is coming.

Wind speed and direction were recorded at a frequency of 1 Hz using a CR1000X data logger. We collected data daily and processed it using PC400 (version 4.7), software provided by Campbell Scientific. Our script uses all default settings provided by Campbell Scientific, but adjusted the scan interval to 1 second for 1 Hz data logging. We also programmed the azimuth angle (discussed above) of 35.3°.

After collecting data, we combined all files corresponding to a single date (in UTC). During data cleaning, we removed any gaps or repeats in the dataset. Gaps were 2-5 second in length, and none occurred during aircraft testing periods. We replaced data gaps with NA values. There were also occasional repeated timestamps in the data: a timestamp would appear once with wind data entered in each relevant field, with the same timestamp appearing again with blank entries in each field. In each such instance, we deleted the redundant (empty) timestamp.

On November 4th, 2022 we experienced equipment malfunction with data collection, and no data are available from the 3-D anemometer for that date.

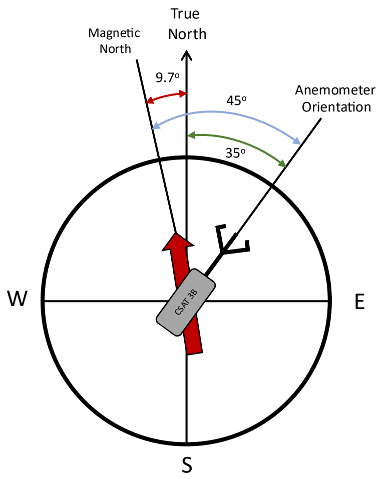

Figure S8 Orientation of CSAT 3B relative to True North and Magnetic North, with a 9.7° declination. Azimuth angle refers to the angle between the anemometer orientation and North. The azimuth angle relative to Magnetic North was measured to be 45°, and this value was adjusted to account for the magnetic declination (9.7°) to determine the azimuth angle of 35° relative to True North.

1.1.5 2-D ultrasonic anemometer

A two-dimensional (2-D) ultrasonic anemometer (Gill Instruments, Windsonic 60) was mounted on a tripod at 2-meter height at coordinates [32.8219591, -111.7851434], measured via iPhone Google Maps pin drop. The 2-D anemometer was 59 meters (194 feet) from the release stack. The indicator on the anemometer was oriented towards North, as per manufacturer's instructions. The size of the indicator was such that it was not feasible to reliably differentiate the between True North and Magnetic North given the declination of $< 10°$. While we logged 2-D anemometer data daily, it was only used in analysis for November 4th when 3-D anemometer data was not available (discussed above).

1.2 Data processing for raw meter data

1.2.1 Metering trailer data log

Data from the metering trailer was collected on a Stanford laptop using the program Configuration: Node-Red which outputs a CSV data log with secondly timestamps and corresponding columns for each solenoid valve, gate valve, and flow path. The data log indicates whether or not each solenoid valve was open, the percent to which the gate valve is open, as well as providing the metered flow through each flow path. The format of the data log was programmed by VINCEENGINEERING, PLLC (Salt Lake City, Utah), and modifications were made based on requests by the Stanford team throughout the testing period. Due to limitations in the programming of the software control system, we rely on the metered data collected directly from the Coriolis meters themselves, as opposed to from the data log generated from Node-Red. However, we use data from the metering trailer (or flowskid) data log to determine whether or not the solenoid valves are open, discussed in greater detail below.

1.2.2 Coriolis meter historical files

On November 3rd, 2023 we collected the historical data from all three Coriolis meters. Data recorded on each meter began October 3rd. Subsequently, for each day of testing, we collected historical data for all meter used. Figure S9 summarizes the data cleaning process for the historical Coriolis meter files. First, we applied necessary timestamp corrections to enable merging the Coriolis meter data file with the flowskid data log. Briefly, the CMF050M internal clock lagged 10 minutes and 10 seconds behind the other meters, likely because of its previous purchase date. The flowskid data log timestamps were adjusted to UTC time using side-by-side photographs of the laptop clock and an iPhone displaying the time from World Clock (timeanddate.com/worldclock/). All adjustments to flowskid data log timestamps are summarized in Table S5. Additionally, for the CMFS015H and CMFS150M meters, we removed all historical data from before the meters arrived onsite. Mass flow units of the meter files were also converted from whole gas kg/s to kg/hr.

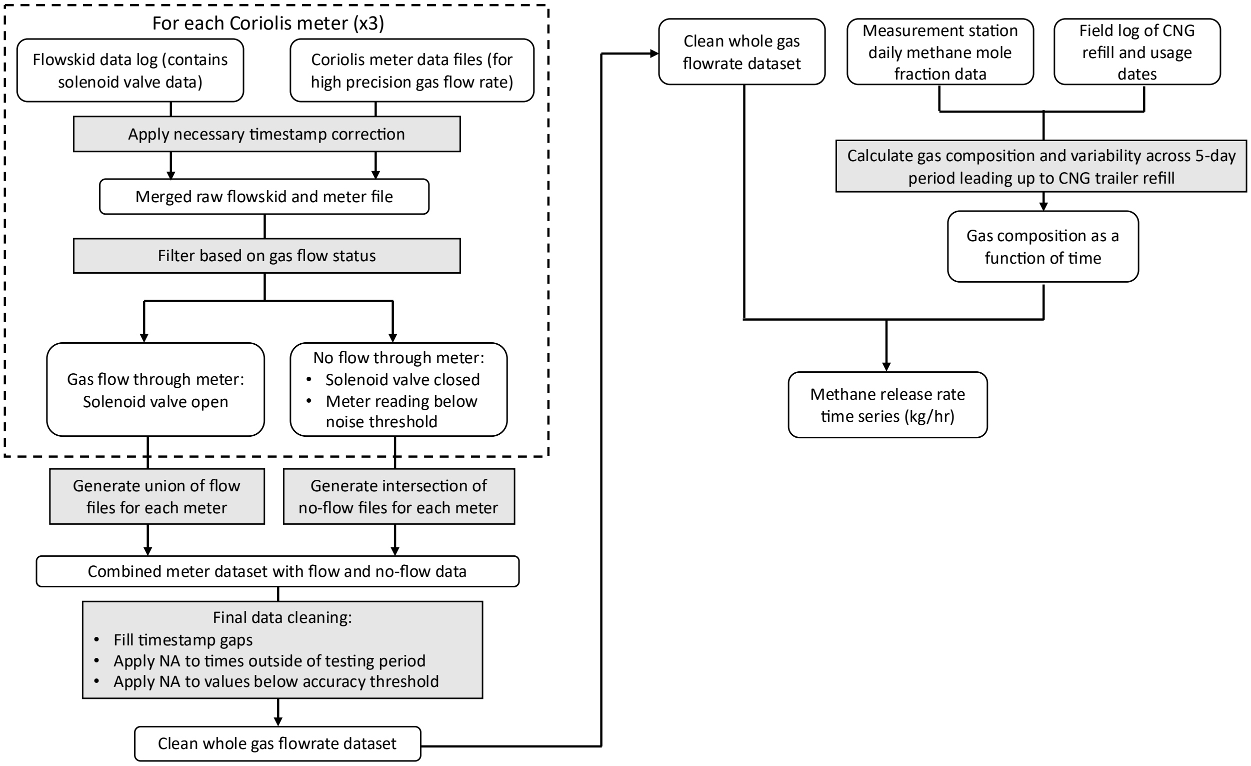

Figure S9 Data processing flow chart for determining methane release rate (kg(CH_4)/hr), using flowskid data log and Coriolis meter raw data files.

Table S5 Summary of timestamp adjustments to flowskid data log. All comparisons are between the data logging laptop and World Clock UTC on iPhone. On October 10th, the data logging computer system clock had not been calibrated to UTC time, and a 17 second adjustment was made after the first day of testing. Flowskid laptop was then set to British Summer Time (BST), and aligned with UTC World Clock time. On October 13th, British Summer Time shifted by 1 hour, resulting in a delay compared to UTC. On October 19th, we changed the laptop system clock to UTC time.

Date	Timestamp Correction to Flowskid Data Log
2022-10-10	Flowskid log 17 seconds behind World Clock UTC
2022-10-11	Flowskid log aligned with World Clock UTC (laptop system clock set to British Summer Time)
2022-10-13 through 2022-10-19	Flowskid log 1-hour behind World Clock UTC (British Summer Time time change, resulted in 1-hr offset)
2022-10-22 and after	Flowskid log aligned with World Clock UTC (laptop system clock set to UTC)

After merging the two file types for each meter, the data is filtered to generate two datasets for each meter: one in which gas was flowing through the meter, and one in which it was not. Gas was determined to be flowing through the meter if the solenoid valve status was set to open. For a meter reading to qualify for the “no-flow” dataset, it must meet two criteria: the solenoid valve must be closed and the meter reading must be below a noise threshold of 0.2 kg/hr. Both criteria are required because the field team would close the solenoid valve when flow rate was at 5 to 20 kg/hr, meaning the solenoid status was not sufficient for determining if no gas was flowing through the meter or not. We use this noise threshold instead of setting a required flow rate to 0

kg/hr because the CMF050 flow readings included noise of up to 0.2 kg/hr when the meter was definitively not in use (likely due to random vibration or other noise sources in the system).

Gas flow and no-flow data files for each file where then combined to generate flow and no-flow datasets across all meters. To generate the dataset of gas flow rates, we use the union of all three meter flow files. To generate the dataset of no-flow periods, we determine the intersection of the three no-flow meter files: in other words, all three solenoid valves must be closed and gas flow rate through each meter must be less than the noise threshold. Meter measurements where the solenoid valve is closed but flowrate is greater than the noise threshold are re-added in the final data-cleaning stage. During this final cleaning, we also set meter readings outside of testing periods to NA. We also set metered values to NA if the measured flow rate resulted in a percent error greater than 2% of the flow rate, per manufacturer recommendation. Flow rates for each meter corresponding to this error threshold are summarized in Table S6.

Table S6 Meter accuracy threshold values. Low-bound flow cutoff for accuracy less than 2%, calculated using Emerson MicroMotion online sizing tool. We aimed to maintain flow rates within target ranges listed in Table 3, whereas these values represent lower bounds of meter accuracy and are used for data cleaning only.

Meter	Meter Model	Flow Rate Accuracy Threshold
Small	CMSF015H	0.56 kg/hr
Medium	CMF050M	3.87 kg/hr
Large	CMFS150M	40 kg/hr

There are periodic gaps in the secondly metered data, which are filled in the final stage of meter data cleaning. Communication with Emerson indicated that data gaps are likely caused by the transmitter power cycling due to unstable power supply the onsite portable generator. Table S7 summarizes the length of gaps in seconds and total number of occurrences across all metered data, and total number of occurrences during non-zero flow rates. Gaps represent 2.26% of all non-zero secondly measurements conducted during testing days, and all but 14 gaps had a duration of 7 seconds or shorter. We fill gaps in data using a linear interpolation between the two measurements on either side of the gap.

Table S7 Summary of gaps in Coriolis meter data across all days of testing. There were a total of 6,520 gaps in the data, of which 4,054 occurred during non-zero releases. These missing data made up 4.13% of all data, and gaps that occurred during non-zero releases are 2.26 % of total data. Total number of secondly measurements in the dataset is 713,975 and total number of seconds of missing data (including those occurring during zero releases) is 29,543 seconds. The longest gap length is 244 seconds, or about 4 minutes, and occurred during a zero-release. The longest gap during a non-zero release was 16 seconds.

Gap Length (seconds)	No. of Occurrences During Non-Zero Releases	Total No. of Occurrences
1	1,444	1,747
2	2	2
3	928	1,538
4	0	2
5	0	1
6	2	2
7	1,664	3,202
8	0	0

9	0	0
10 ≤ t < 20	14	22
20 ≤ t < 200	0	2
100 ≤ t < 200	0	1
200 ≤ t < 300	0	1
**Total No. of Gaps**	**4,054**	**6,520**
**Fraction of Total Data (%)**	**2.26%**	**4.13%**

Whole gas flow rates are provided for each day of testing. For each secondly timestamp (in UTC), we include the release rate in kg/hr of whole gas, and list the meter that was used for the measurement. We also include a data QC column to indicate which data was below the meter accuracy threshold (2%) or an interpolated value as described above. In the raw data files for each date, a QC flag of 1 indicates a non-testing period, 2 indicates non-original interpolated data, and 3 indicates the flow range was below the accuracy level of the meter in use. Note that non-original interpolated data can exist outside of testing periods, in which case the QC flag would still be 1, whereas Table S7 summarizes gaps in data on testing dates during both testing and non-testing periods of testing days.

1.2.3 Gas compositional analysis

We converted from whole gas flow rate to methane flow rate using gas composition data. Two gas sample canisters were collected and analyzed by an independent laboratory from each tank of gas, and gas composition data were also collected from two measurement stations upstream of the CNG fill point (see Supplemental Information Section S1.2.3. for more details). In the field, we noted each time the CNG trailers were filled, and the point at which we switched to using gas from a new supply. Trailer fill dates were matched with the gas compositional data. To account for variability in gas composition and latency between the measurement station and the CNG station, we average the data from both measurement stations for the five days leading up to and including the date of a given truck refill. The standard deviation across this period is used for determining uncertainty associated with gas composition. Mean mol% CH_4 over the study period is 94.53% and the standard deviation is 0.62%.

We converted from whole gas flow rate to methane flow rate using gas composition for the CNG supply used in this experiment. In the field, we noted each time the CNG trailers were filled, and the point at which we switched to using gas from a new supply. Trailer fill dates and usage are then matched with the corresponding compositional analysis. We then used two methods for determining gas composition: measurement station compositional data and laboratory sample analysis.

All primary analysis uses gas compositional data from the two nearest upstream measurement stations on the pipeline that supplied the station at which our gas trailers refilled. We obtained datafiles for gas composition provided by the gas supplier that serves the CNG station. To account for variability in gas composition and latency between the measurement station and the CNG station, we average the data from both measurement stations for the five days leading up to and including the date of a given truck refill. We use gas composition on these days to determine both mean methane fraction and the standard deviation, as an indicator of variability of gas composition. In the field, we documented the start time of each new trailer batch, and thus

matched gas composition with specific releases. Table S8 shows the average gas composition and standard deviation on specific dates of the experiment. These are the values used in the primary analysis and ground truth meter data provided to participants.

Table S8 Gas composition for each truck refill, using data from upstream measurement stations. When we used a new truck at the start of the day, we set start time to 0:00 for simplicity in coding.

Batch No.	Batch Start Date (UTC)	Batch End Date (UTC)	Average Percent Methane	Standard Deviation of Percent Methane
1	10/5/22 0:00	10/12/22 18:12	93.6%	0.162%
2	10/12/22 18:12	10/19/22 0:00	93.9%	0.196%
3	10/19/22 0:00	10/25/22 17:42	94.6%	0.241%
4	10/25/22 17:42	10/28/22 0:00	95.1%	0.137%
5	10/28/22 0:00	10/29/22 16:00	95.0%	0.136%
6	10/29/22 16:00	10/31/22 0:00	95.3%	0.461%
7	10/31/22 0:00	11/1/22 16:00	95.4%	0.365%
8	11/1/22 16:00	11/8/22 0:00	95.0%	0.269%
9	11/8/22 0:00	11/9/22 0:00	95.3%	0.132%
10	11/9/22 0:00	11/11/22 0:00	95.4%	0.166%
11	11/11/22 0:00	11/15/22 16:56	95.4%	0.203%
12	11/15/22 16:56	11/17/22 18:47	95.1%	0.127%
13	11/17/22 18:47	11/21/22 16:00	94.6%	0.528%
14	11/21/22 16:00	11/28/22 0:00	94.1%	0.093%
15	11/28/22 0:00	12/1/22 0:00	94.2%	0.100%

In the field, we had also collected samples from each CNG refill for analysis at Eurofins Air Toxics Laboratory. We sampled gas from the pressure regulation trailer by connecting laboratory supplied canisters to the RT-30 sampling port (described above). Canisters were sent to Eurofins Toxics Laboratory in Folsom, CA for gas composition analysis. Initially, we collected gas using the vacuum gauge supplied by the Eurofins to indicate fill level. Canisters were connected to a vacuum gauge, and reading prior to fill was typically 25 – 30 Hg of vacuum. We opened sampling port to begin filling the canister and allowed the vacuum gauge to reach 0 Hg. After receiving laboratory results indicating gas pressure in the canister remained lower than 0 Hg, we increased fill time to 1 minute and then to 5 minutes, switching methods at the dates listed in Table S9. Table S9 also summarizes fill method and time for each sample. We discarded all canisters that arrived at Eurofins laboratory with a pressure below 15 Hg vacuum.

Table S9 Gas Refill schedule, including fill method or fill time for each gas sample collected from the pressure regulation skid

Sampling Dates	Sample ID	Fill Method
Oct 10 – Oct 29	01 through 12	Vacuum gauge reading
Nov 1 – Nov 14	12 through 17	1-minute fill time
Nov 14 – Nov 28	18 through 27	5-minute fill time

We requested quantification of the following compounds using Modified Method ASTM D-1945: methane, oxygen, nitrogen, carbon monoxide, ethane, ethene, acetylene, propane, isobutane, butane, neopentane, isopentane, pentane, and hydrogen. However, we ultimately chose not to use the gas compositional data reported by Eurofins for several reasons. First, for most trailer refills, we collected two samples from the same truck (see Table S10 below). However, Eurofins results for these replicate samples could be offset by up to 4%. Additionally, ten of the samples reported 100% methane, an unrealistic composition for compressed natural gas. For these samples, the sum of all analyzed constituents was greater than 100%. Finally, personal communications with Eurofins Technical Director indicated that the laboratory instruments have an uncertainty of +/- 4.5% [95% CI], far greater than the observed variability in the more precise gas measurement station data described above. For these reasons, we consider the Eurofins results to be unreliable and opt to use the analysis provided supplier measurement stations.

Table S10 compares the average percent methane for each truck refill using raw Eurofins data, normalized Eurofins data, and measurement station data. Data from the measurement station were reported with higher levels of precision than Eurofins data (five significant figures vs. two significant figures). Also, the sum of all components analyzed in the measurement station data is always within 0.002% of 100% while the sum of all components in the Eurofins data reached over 105% in some samples.

Table S10 Gas composition summary . Comparison of CNG gas percent methane using three methods: Eurofins raw reported values, Eurofins values normalized such that the sum of all constituents is 100%, and mean measurement station values. Where more than one Eurofins canister was collected per refill, we report the average of the two values. We did not collect a sample for Truck Refill 10, and the reported value for Eurofins data is the average of canisters 16, 17, and 18. Measurement station values are the average of the measurements of the two taps for the five days leading up to and including the date of refill (as summarized previously).

Refill No.	Eurofins Canister ID	Eurofins Raw (% CH_4)	Eurofins Normalized (% CH_4)	Measurement Station (% CH_4)
1	01	94%	94%	93.636%
2	03	92%	92%	93.909%
3	05	90%	90%	94.602%
4	06	88%	88%	95.064%
5	08, 09	87%	87%	95.029%
6	10, 11	92%	92%	95.332%
7	12, 13	96%	93%	95.429%
8	14, 15	96%	93%	95.034%
9	16, 17	100%	96%	95.337%
10	NA	100%	95%	95.367%
11	18	100%	95%	95.389%
12	20, 21	100%	95%	95.094%
13	22, 23	100%	96%	94.637%
14	24, 25	100%	95%	94.112%
15	26, 27	99%	94%	94.227%

1.3 Aircraft testing

1.3.1 Field testing conditions

We tested five different aircraft technologies from October 10th through November 11th, 2022. Field measurement protocols were based on those previously reported to maintain consistency and comparability with other testing results (Rutherford et al., 2023). Operators were asked to recreate typical or commercial flight operations as closely as possible. Each operator submitted key measurement parameters prior to the start of testing, including time necessary for measurement, planned flight lines, flight altitude, and predicted lower detection limit. Pre-scheduled testing dates avoided simultaneous tests of technologies. However, due to scheduling limitations on the operator side, supply-chain delays affecting equipment on the Stanford side, and flight-prohibitive weather conditions, this was not always possible and real-time adjustments to flight days were required. The final testing dates and flight altitude are depicted in Table 1 of the main text.

For the spectroscopy-based technologies, we set and then held a release rate while the aircraft passed overhead, following a pre-planned flight trajectory. We typically held a constant rate for multiple overpasses of a given aircraft, aiming to change release rates at least two minutes before the next expected overpass, although this was not always possible for aircraft with shorter measurement times or on days when we tested multiple aircrafts at the same time. The Stanford ground team tracked the GPS location of each aircraft being tested using the FlightRadar24 mobile app. For Insight M, we used a Spidertrack link provided by the company. While we documented timestamps when the aircraft appeared to directly overhead, all subsequent data processing used timestamps based on digital GPS tracking. For these spectroscopy-based technologies, a measurement occurs when the aircraft passes over the release site and reports the overpass to Stanford. We use a measurement timestamp based on the moment when the GPS coordinates of the aircraft are closest to directly above the release stack.

Scientific Aviation conducts continuous data collection over a 20 – 40 minute time period, and an individual measurement refers to one such flight period. Because we were conducting multiple 20-minute releases throughout the testing period to test satellite-based methane sensing, and we created a fixed release schedule to align satellite releases with Scientific Aviation measurements (included in Table S11). To avoid providing Scientific Aviation with any potential information about release rates, we did not indicate which release periods aligned with those of the satellites. The Scientific Aviation aircraft arrived onsite for the start of the release period, and conducted measurements for the length of time determined by the Scientific Aviation scientific team on board the aircraft. If they completed all necessary measurements before the end of the release period, they left the site to return for the start of the next release period. Based on input from Scientific Aviation, the initial release duration was set to 40 minutes. On November 11th, this was reduced to 35 minutes.

For Scientific Aviation, a measurement refers to the period over which the aircraft was in proximity of the release point and collecting data, with start and end times as submitted in the Scientific Aviation results report.

Table S11: Coordinated release schedule for Scientific Aviation testing. The Stanford team set a new release rate and the time indicated on the table, and Scientific Aviation would arrive onsite and begin measurement protocols shortly thereafter. If Scientific Aviation completed all necessary measurements before the rate change, the plane left the immediate area to return measurement window in the schedule. The schedule was designed such that the Stanford team could simultaneously conduct satellite releases (20 minutes long) without providing information on which measurements were coincident with satellites to the Scientific Aviation team. All times are in local Arizona time.

November 8th	November 10th	November 11th
14:35	11:00	12:00
15:15	11:40	12:35
15:55	12:20	13:10
16:35	13:00	13:45
	14:20	14:20
	14:50	14:55 – Refuel
		15:30
		16:10

For all operators, the Stanford ground-team was in communication with the flight operations team via radio or text message. At the start of each scheduled flight day, the Stanford team would send an image of the sky overhead to operators to allow them to determine if onsite cloud conditions were conducive for measurement. Subsequent communication between the ground team and flight operations team was kept to a minimum, and limited to the following topics: communication regarding clouds onsite or over the release point, or any local field disturbances and associated deviations from flight patterns. Table S12 documents all notable deviations from flight patterns or field procedures.

Table S12: Summary of aircraft deviations from typical flight patterns or measurements

Datetime (UTC)	Operator	Notes
2022-10-26 16:58	Insight M	Delay between measurements due to hot air balloon taking off within proximity of the field site
2022-10-28 19:04	Carbon Mapper	Aircraft conducted atypical circular flight pattern
2022-11-02 17:28	GHGSat-AV	Aircraft conducted atypical flight pattern; circling above field site due to cloudy conditions
2022-11-02 17:52	GHGSat-AV	Aircraft conducted atypical flight pattern; circling above field site due to cloudy conditions, testing for the rest of the day cancelled shortly after
2022-11-10 21:50	Scientific Aviation	Aircraft ended measurements early upon reaching fuel limit; Scientific Aviation team reported to Stanford team while in the field
2022-11-10 18:15	Scientific Aviation	Stanford team switched CNG trailer, causing an increase in flow rate associated with the sudden increase in trailer pressure;

		This measurement was removed through Stanford filtering
2022-11-11 23:03:12	Scientific Aviation	Power outage onsite, immediately shutting off all gas flow. Stanford field team informed Scientific Aviation field team that power had been cut, and the next measurement started with a delay to allow power restart

1.3.2 Description of Technologies Tested

We tested five different aircraft-based methane sensing platforms in this study: Carbon Mapper, GHGSat-AV, MethaneAIR, Insight M, and Scientific Aviation. As discussed in the main text, all platforms except Scientific Aviation use spectroscopic imaging-based measurement, while Scientific Aviation uses an in situ measurement approach. We requested sample plume images from all test participants. At the time of this manuscript submission, only Carbon Mapper and MethaneAIR provided a plume image.

1.3.2.1 Carbon Mapper

In this study, Carbon Mapper operated the Global Airborne Observatory (GOA). This aircraft is equipped with a visible / infrared imaging spectrometer integrated on a Dornier aircraft. The spectrometer measures reflected solar radiation in the visible-to-shortwave infrared (380 – 2,510 nm) with 5-nm spectral sampling (Duren et al., 2019). At a flight height of 3 km (10,000 feet) above ground level, as in this study, the instrument typically has a 1.8-km field of view and 3-m pixel resolution (Duren et al., 2019). Data processing pipeline for Carbon Mapper is described in the Performer Info tab in the Carbon Mapper data report in the Github repository for this manuscript. Figure [X] includes to sample plume images generated by Carbon Mapper, including the RGB image, raw methane concentrations, and the methane concentration overlaid on the true-color base.

Carbon Mapper, Inc. is a non-profit organization funded by philanthropy with the mission of identifying and tracking methane and carbon dioxide emissions (Carbon Mapper, 2023).

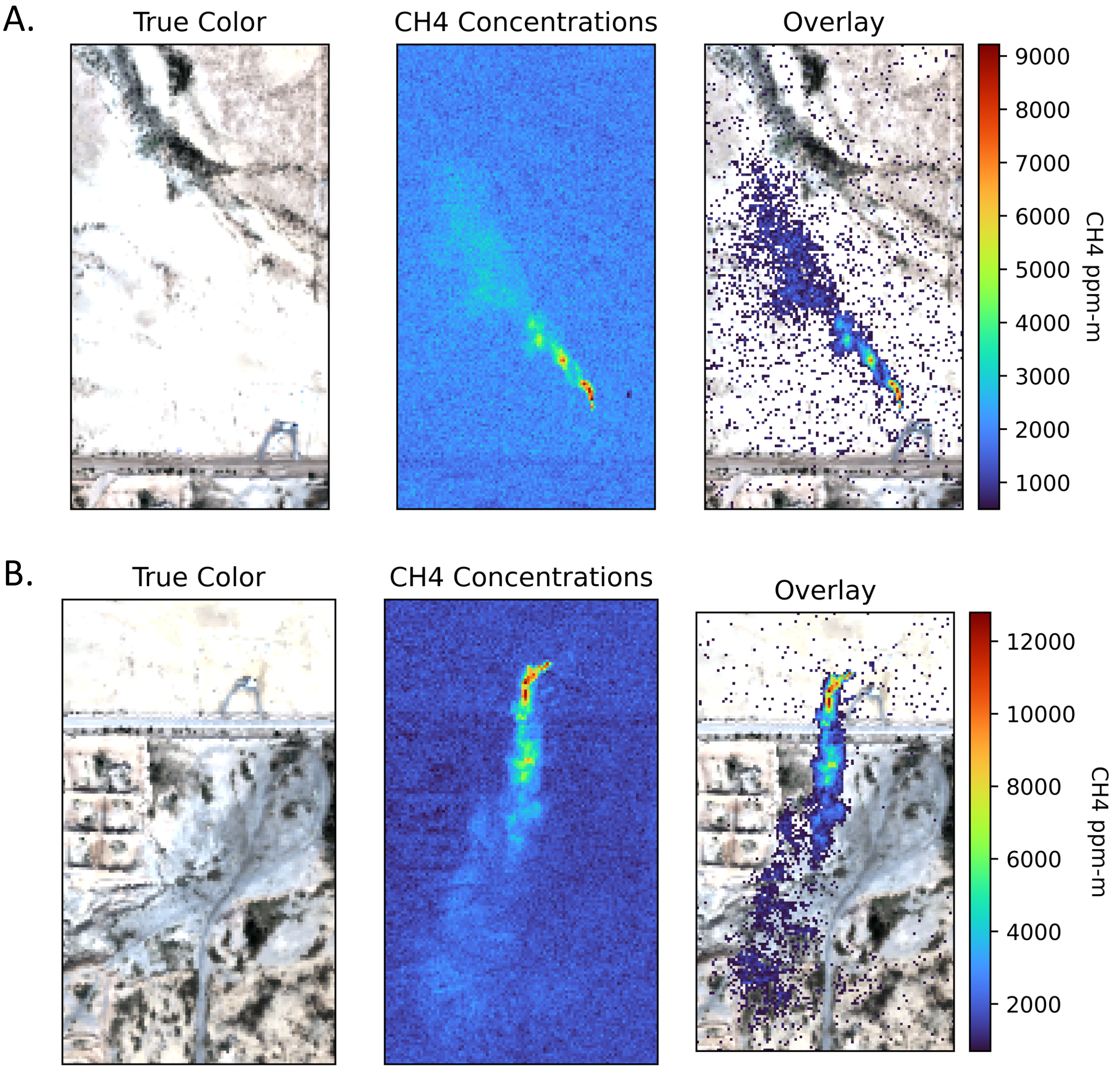

Figure S10 Sample Carbon Mapper plume images, including RGB image (left), methane concentration (middle) and an overlay of the methane on the true color image. Plumes are from A. October 21 18:15:30 UTC and B. October 31 at 19:53:53 UTC.

1.3.2.2 GHGSat-AV

In this study, GHGSat flew a C-GJMT aircraft equipped with the GHGSat-AV2 sensor. The sensor leverages similar technology to GHGSat's corresponding satellite sensor (GHGSat-Cx), a wide-angle Fabry-Perot sensor with pixel resolution of <1 m (Esparza et al., 2023). At a flight altitude of 3,000 meters, slightly above the 2,000 meter above ground level flown in this study, GHGSat report a swath width of 750 m (Esparza et al., 2023). For details of the flight operations for this study, see the Performer Info tab on GHGSat report submissions in the Github repository.

GHGSat Inc. is a private company that offers commercial methane detection services through both satellite and aircraft platforms (Esparza et al., 2023).

1.3.2.3 MethaneAIR

MethaneAIR is the aircraft-based precursor to the upcoming satellite mission MethaneSAT, developed by MethaneSAT, LLC (Chulakadabba et al., 2023). The MethaneAIR spectrometer measures methane enhancement in the 1,650 nm band and a 10m x 10m spatial resolution (Chulakadabba et al., 2023). Designed for wide spatial coverage, the instrument has a swath width of 4.5 km when flying at 12,960 m above ground level (Chulakadabba et al., 2023), the altitude used in this test. Additional details on the MethaneAIR measurement procedures and data processing are included in the MethaneAIR operator report in the GitHub repository. Figure S11 provides an example plume image output from this study, provided courtesy of MethaneAIR.

MethaneSAT, LLC is a wholly owned subsidiary of the nonprofit Environmental Defense Fund, with the mission of providing accurate and rapid quantification of methane emissions (MethaneSAT, 2023).

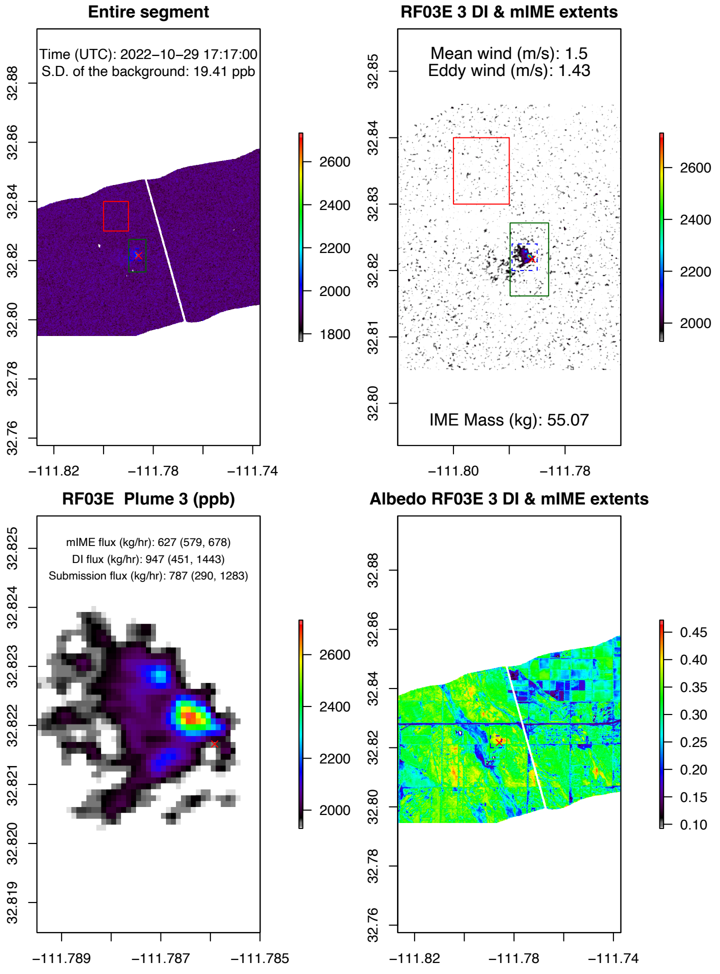

Figure S11 Sample MethaneAIR plume images provided by MethaneAIR

1.3.2.4 Insight M

Insight M (formerly Kairos Aerospace)measures methane using LeakSurveyor™, an infrared imaging spectrometer with 3 m resolution (Sherwin, Chen et al., 2021). In previous testing, an aircraft flies at an altitude of 900 m (3,000 feet) above ground level, nearly twice the flight altitude of the current study (Sherwin, Chen et al., 2021). Typical flight configuration involves a single wing-mounted unit for generating infrared and optical images during survey (Sherwin, Chen et al., 2021). In the current study, Insight M operated with two measurement units, one mounted on each wing of the aircraft. Additional details on the quantification algorithm are included in Sherwin, Chen et al., 2021 and further information on the Insight M measurement procedures and data processing for this study are included in the operator report in the GitHub repository.

Insight M is a for-profit company that conducts aircraft surveys to identify oil and gas methane emissions, and facilitate rapid action and repair (Insight M, 2024).

1.3.2.5 Scientific Aviation

Scientific Aviation uses an in situ measurement approached described in Conley et al., 2017. While conducting laps around an emission source, the aircraft collects ambient air and measures methane concentration using a Picarro cavity ring down spectrometer (Conley et al., 2017). In this study, methane measurements were conducting using a Picarro 2210-m, and analysis of other compounds is feasible as well (discussed in the main text). Real-time analysis informs the number of laps and altitude (Mackenzie Smith, personal communication).

Scientific Aviation is a ChampionX research company that provides commercial methane detection services using aircraft, ground-based and drone platforms (Scientific Aviation, 2023).

1.3.3 Data reporting and unblinding

Operators submitted results using a template provided by Stanford, subject to modifications by the operator as necessary. Timestamps for each individual measurement were documented by the Stanford field team and reported by operators. For Carbon Mapper, GHGSat-AV, and Methane Air, aircraft GPS coordinates and altitude were downloaded from FlightRadar24. Insight M used Spidertrack for flight monitoring during testing, and provided positional and altitude data after the fact. Because different operators use different methods for reporting measurement timestamps, we use flight tracking GPS coordinates for consistency. Thus, timestamps of measurements refer to the moment when the distance between aircraft GPS coordinates and the coordinates of the release stack are at a minimum. For Scientific Aviation, the Stanford team cannot independently ascertain when data collection occurring in the aircraft starts and stops. Thus, we used the measurement start and stop time as reported by Scientific Aviation in their data report.

Overpasses documented by Stanford and on FlightRadar24 or Spidertrack that were not included in the operator report are classified as “missing data” in figures of the main text. This occurred if

the aircraft flew above the release site, and thus was documented by Stanford ground team and GPS tracking as an overpass, but no measurement was conducted by the field team. The Stanford ground team documented 711 airplane overpasses, of which 704 were matched with operator reported measurements. The number of missing measurements for each operator are summarized in Table S13. All GHGSat-AV missing measurements were reported by the operations team to the Stanford team during testing. Carbon Mapper and MethaneAIR missing measurements were identified after results were submitted, during data analysis. Insight M and Scientific Aviation did not have any missing measurements. Because we do not have access to sensors or processing software used by the operators, we cannot determine reasons why any particular measurement would have failed.

Table S13 Total number of missing measurements by operator. A missing measurement occurs when an overpass is documented by GPS coordinates and Stanford field team observation, but no corresponding measurement is reported in operator results.

**Operator**	**Number of Missing Measurements**
Carbon Mapper	3
GHGSat-AV	2
Insight M	0
MethaneAIR	2
Scientific Aviation	0

Operators had the option to participate in a multi-stage unblinding process. For Stage 1, operators submitted fully-blinded results report. Next, the operator was provided with Stanford measured 10-meter wind data and allowed to submit revised quantification estimates. After submitting Stage 2 revised results, operators were provided a subset of metered gas flow data. In Stage 3, operators could use these data to make any additional revisions to their results for final submission (described below).

1.3.4 Data processing for aircraft testing

For each operator, we set the gas release rate based on our desired sampling strategy. However, we were unable to set rates with full precision due to the technical limitations of our system. Thus, we identified a target release range while in the field, and manually documented flow rates for each aircraft measurement. Ideally, we would have an automated log of all set points, but did not because of software issues. As a result, we developed an automated method to determine changes in the release rate, which we describe here. Figures S11 and S12 shows outputs of the plume definition algorithm for two days of testing (outputs for all days are included in the Appendix). One shows the plume definition criteria as applied to a test date when we used the automated feedback system to set the flow rate, and the other shows a date when we set the flow rate using the valve position.

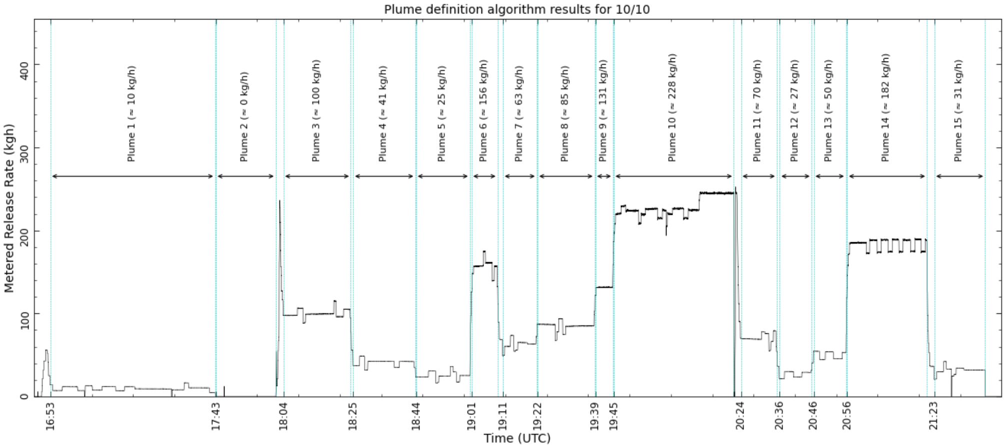

Figure S12: Results of plume identification algorithm when system flow rate was set using an automated feedback (PID) system.

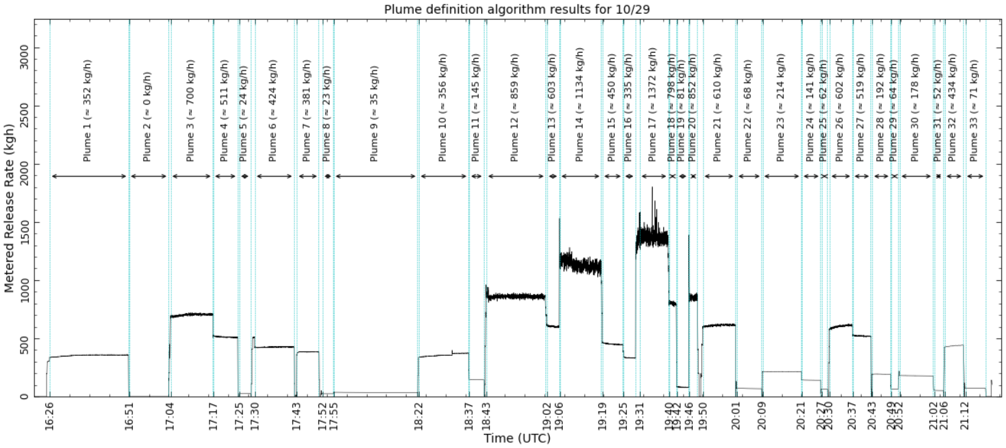

Figure S13: Results of plume detection algorithm when system flow rate is set using a fixed valve positions. Note change in y-axis compared to Figure S10.

During aircraft testing, a single release rate was often held for multiple aircraft overpasses. The release rate was then changed directly to a new release rate, without allowing for a pause or zero release in between two different release rates. Here, we use the term "release" to refer to one such constant release rate, during which one or multiple aircraft overpasses and corresponding measurements may have occurred. Defining individual releases was necessary for providing aircraft operators with a subset of their data in Stage 3 of unblinding, described further below.

In order to define an individual release, we identify periods of steady flow rate given allowable tolerance for noise in meter measurement and flow variability. There are two sources of noise in meter readings which occur when the desired flow rate is set:

1. Meter noise: noise inherent to the instrument reading of gas flow rate. We quantified typical meter noise across each meter's calibrated range by holding steady release rates for several minutes and calculating the associated deviation from the mean (see Table

S14)

2. Overcorrection-associated noise: from October 10th – 20th, over-correction by the automated feedback system (Proportional – Integral – Derivative or PID controller), contributed to increased fluctuations in flow rate (an example of this is shown in Figure S6 above).

We observed lower levels of meter-associated noise in the small (CMFS015H) and medium (CMF050M) meters compared to the large (CMFS150) meter. Meter noise across all three instruments was lower than the PID-associated noise, which displayed greater amplitude and variability. Defining releases required us to separate meter and PID-associated noise from the flow variability that occurs during the transition from one release rate to a new release rate. Thus, for each meter we define a maximum allowable deviation function (see Table S14).

Maximum deviation equations accounts for both meter and overcorrection-associated noise, and are a function of the date, flow rate, and meter used for the measurement. For the small (CMFS015H) and medium (CMF050M) meters, we selected values for maximum deviation that were three times the typical standard deviation observed over a 3-minute period. For the medium meter, we added a correction factor to account for the PID-associated variation, for periods when PID system was used. Variability in the large (CMFS150) meter increases at higher flow rates. The equation in Table S14 was determined using the standard deviation of flow rates ranging from 180 kg/hr to 1600 kg/hr over 1-3 minute periods.

Table S14 Maximum allowable deviation in meter reading for defining individual releases. Equations are based on typical standard deviations observed in meters across their calibrated range.

Meter	Empirically Determined Deviation Function
CMFS015H	$0.12 * flow\ rate$
CMF050M	*If date is before 2022-10-20:*<br>$5 + 0.2 * flow\ rate$<br>*If date is after 2022-10-20:*<br>$0.12 * flow\ rate$
CMFS150M	*If flow rate ≤ 700 kg/hr whole gas:*<br>65<br>*If flow rate >700 kg/hr whole gas:*<br>$-94 + 0.24 * flow\ rate$

To identify unique releases, we iterate through meter data. Logic for this function is summarized in Figure S13. First, we compare the flow rate at a given time point t with the mean flow rate since the end of the previous release. If the difference between the two rates is less than the maximum allowable deviation, the mean is updated to include time t, and we continue to the time $t+1$. When the function reaches a timepoint t where flow rate is greater than the allowable deviation, we may have reached the point that marks the end of one release rate and the beginning of another.

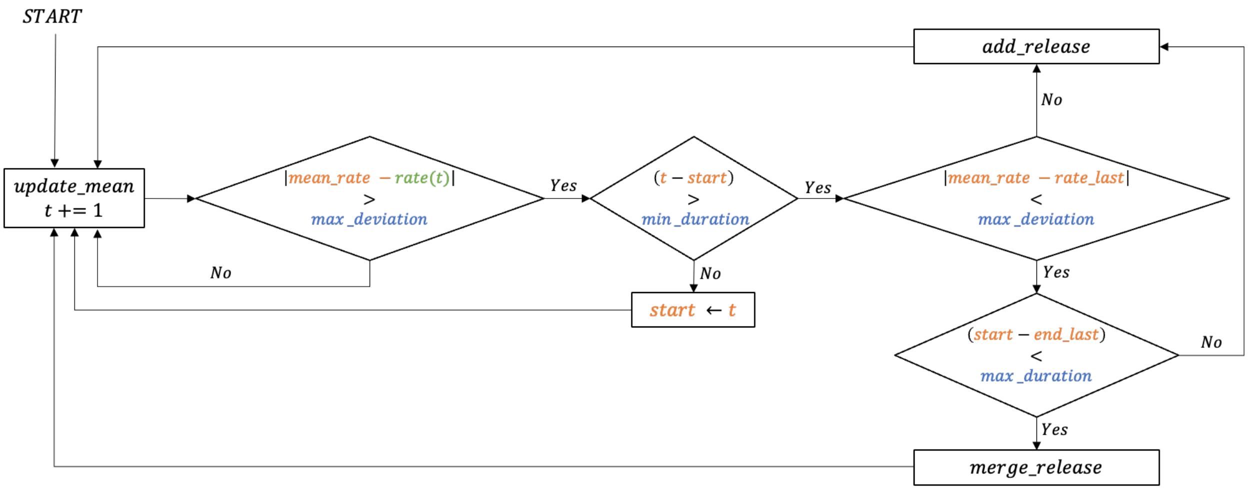

Figure S14 Flow chart for release categorization algorithm. The variable mean_rate is the average release rate for the current averaging period, rate(t) is the flow rate at time t, and rate_last is the mean flow rate of the last release. The input max_deviation refers to the calculated allowable deviation from mean flow rate, when considering noise from the meter and automated feedback system. Minimum duration (min_duration) is the minimum length of a release, set to 2 minutes. Max duration (max_duration) is the time difference between the end of the last release and the start of the current one, and is set to 10 minutes to allow for merging of releases during periods of high variability caused by the PID-associated noise.

To determine if a new release is beginning, first we must check if the current time over which we are averaging is a period of noise. We define individual releases to be at least 2-minutes in length. Thus, if the averaging period is less than this minimum duration, we consider it to be noise occurring in between true releases. The averaging start time is reset to *t*, and we begin iteration again.

However, if the length of the averaging period is greater than two minutes, we implement one final check to determine if the averaging period is a distinct release. We compare the mean flow rate of the current averaging period with the mean flow rate of the previous release. If the difference between the two is greater than the allowable deviation for the meter of the current release, the averaging period is considered a new release. If the mean flow rate of the current period is close to the mean flow rate of the previous release, as defined by the maximum deviation function, the two releases are instead merged. However, two releases will not be merged if the start of the second release occurs over 10 minutes after the end of the previous release, preventing the merging of plumes with similar release rates but with a time gap between them. When the current averaging period is determined to be a unique release, it is added to a list of releases and the function continues to iterate through meter data to identify subsequent plumes.

1.3.5 Stanford data quality control

1.3.5.1 Spectroscopy-based technology data filtering

Due to highly variable and frequently stagnant wind conditions observed during testing, we developed a quality control (QC) criterion based on wind speed. The goal of this QC step was to ensure gas from earlier releases will not contaminate measurements conducted by aircraft teams. Specifically, for a given aircraft overpass, we look at the gas released in the preceding ten

minutes and determine if the gas is from the current release or the previous release (releases defined using the criteria above). Next, we identify the trajectory of the gas using 3-D wind data collected onsite. Finally, we determine how much of the gas from any previous releases is within a certain distance threshold of release source. If the amount of gas from previous releases and within the distance threshold is greater than a certain percent of the gas from the current release, the measurement is considered contaminated.

This criterion requires determine two specific parameters:

1. Distance threshold: the distance in meters that gas from previous releases must travel in order to be considered *not* interfering with the current release.
2. Mass threshold: the maximum amount of gas from a previous release allowed within the distance threshold.

We made all quality control decisions, including setting the values of both mass and distance thresholds, prior to viewing any blinded data reported by aircraft operators. We determined technology specific distance thresholds for each aircraft operator based on our knowledge of their technology (see Table S15). We use a mass threshold value of 10%, meaning that 10% of the mass within the distance threshold may be from previous gas releases. Because we only use wind transport in modeling the gas plume trajectory, and do not consider dispersion or diffusion, this approach is inherently conservative. Thus, we use a high mass threshold value of 10% as default in our analysis. However, after receiving operator results, we evaluated the error profile each operator using both 10% and 1% mass threshold values. We compared overpasses that failed with the 1% criteria but passed with the 10% criteria with those that passed both criteria, summarized in Table S16. For all operators, we found no statistically significant difference between these two groups using a t-test, with a p-value of 0.05 (a threshold determined prior to viewing any blinded data reports).

Table S15 Distance threshold for each aircraft operator and information used in determining the value

**Operator**	**Distance Threshold [m]**	**Source**
Carbon Mapper	300	Based on maximum fetch distance of 150 m reported in Duren et al., 2019 supplemental information, doubled to add buffer
GHGSat	300	Based on plume images provided to Stanford Team by GHGSAT during 2021 Controlled Release testing, to be published in Rutherford et al, 2023
Insight M	500	Based on plume images from Sherwin, Chen et al., 2021
MethaneAIR	500	Based on plume images provided to Stanford Team by MethaneAIR during 2021 Controlled Release testing, included in Chulakadabba et al, 2023.

Table S16: Summary of quality control comparison imaging technologies, including number of measurements to pass both the 10% and 1% mass thresholds. The threshold comparison column includes the calculated p-value when comparing operator quantification estimates for two groups of measurements: those that passed the 1% mass threshold, and those that passed the 10% mass threshold but failed the 1% mass threshold. Because the threshold comparison value is greater than 0.05 for all operators, we use the 10% mass threshold.

**Operator**	**Pass 1% Mass Threshold (no. of measurements)**	**Pass 10% Mass Threshold (no. of measurements)**	**Threshold Comparison (p-value)**
Carbon Mapper	67	71	0.48
GHGSat-AV	104	121	0.96
Insight M	171	191	0.08
MethaneAIR	18	20	0.85

Figure S14 shows example overpasses that would pass or fail Stanford QC criteria. In both images, we depict the trajectory of gas participles emitted in the ten minutes leading up to the overpass. Points in green represent gas particles emitted from the current release, whereas gas in red represents gas from previous release(s). Within the set distance threshold, if the total mass of red particles is greater than 10% of the total mass of green particles, the overpass is considered contaminated and filtered. In Figure S14, the image on the left passes Stanford quality control filtering while the image on the right fails.

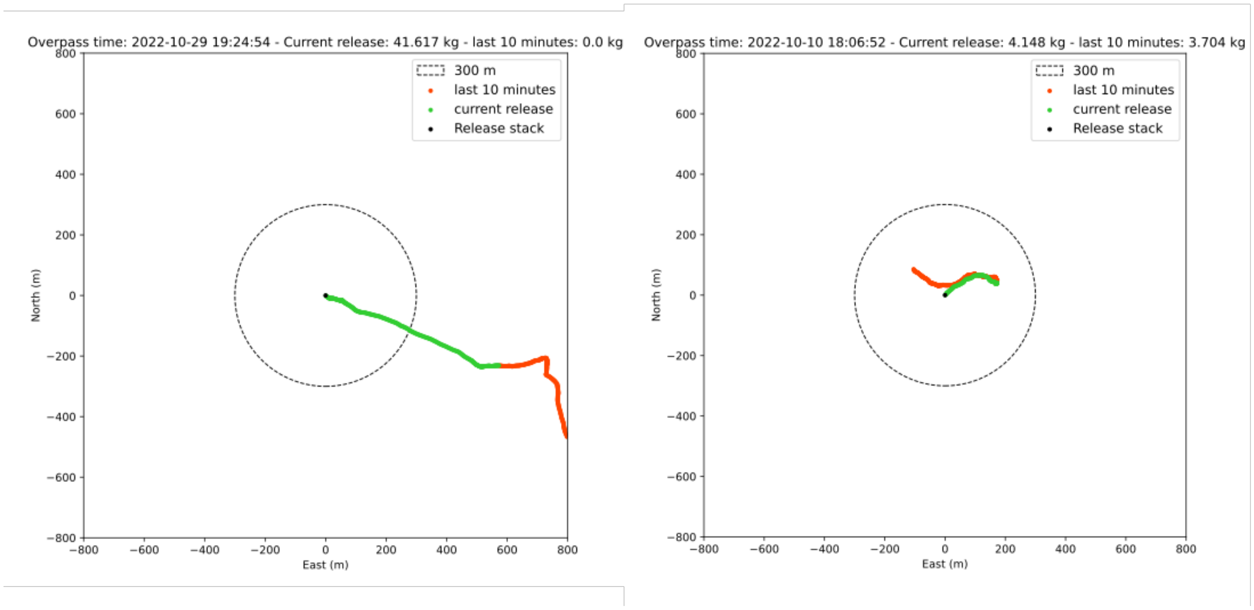

Figure S15 Sample releases that pass and fail Stanford's quality control criteria(left and right, respectively). Colored points represent gas released in the last 10 minutes. Particles colored green are from the current release, and gas colored red are not.

We did not use this wind exclusion criteria for Scientific Aviation, due to the different technological approach to measurement. Rather, we excluded any plumes where the standard deviation of the flow rate was greater than 10% of the mean flow rate. Only one release was removed in this manner.

1.3.5.2 In situ technologies data filtering

Due to the difference in measurement approach, for Scientific Aviation we excluded points based on variability in flow rate. We considered the entire measurement period, as reported by the operator, and excluded any measurements where the standard deviation over this period was greater than 10% of the mean flow rate for the same period.

Using this method, Stanford filtered one measurement from the Scientific Aviation dataset. The high variability in flow rate was the result of changing gas trailers mid-release. While conducting the release, the Stanford ground team observed a steady drop in flow rate, as indicated by the laptop dashboard. Upon communicating with the Rawhide personnel operating gas equipment, we learned this was caused by falling pressure in the CNG supply trailer. The Rawhide personnel switched to the other trailer, which was full and at a much higher pressure. As a result of the increased pressure on the system while maintaining a fixed position of the gate vale, pressure jumped when the source trailer was switched. We attempted to slowly return the flow rate to the previous level, resulting in the slope observed in the second half of the release in Figure S15.

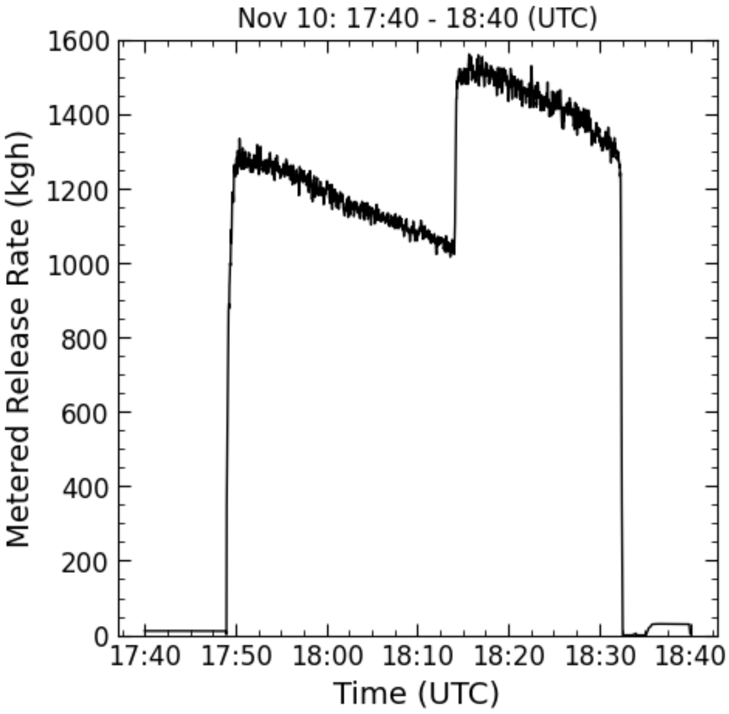

Figure S16: Release for Scientific Aviation filtered by Stanford due to high variability in flow rate (standard devation was greater than 10% of the mean flow rate)

1.3.6 Stage 3 data selection

In Stage 3, our goal was to provide operators with ground-truth data for roughly half of their overpasses, allowing them to revise and resubmit estimates for their remaining blinded measurements. We provided operators with a list of overpass timestamps, as documented by the

Stanford field team, with the associated 60-second time-averaged methane flow rate (in kg(CH_4)/hr). Due to delays in acquiring detailed information on interpreting the irregularities in the gas compositional vales discussed above, we used raw Eurofins data for Stage 3 methane mole fraction. Upstream measurement station data were only acquired after completion of Stage 3.

Stage 3 data were selected to provide roughly half of all overpasses that meet Stanford quality control criteria, while also ensuring the subset is representative of the flow rates for all overpasses. Additionally, we did not want to provide operators with any additional information about overpasses that remained blinded. Thus, when an overpass from one release was selected, we also provided data on all other overpasses that occurred during the same release. With this approach, we avoid a situation in which we provide an operator with overpasses at the start and end of a release, but not those in between – a scenario that could provide operators with information on the true release rate for the middle overpasses that remained blinded.

In order to select which releases to unblind for operators, we first generated a cumulative probability function of the flow rates for all releases conducted for each operator. We then uniformly selected a number of points on this distribution function equal to half of the total number of releases (if we conducted N releases for a given operator, we selected N/2 points on the cumulative probability function). For each probability selected, we then determined the closest maximum gas flow rate with a corresponding cumulative probability lower than the selected probability. This results in a selection of roughly 50% of the releases with a very similar to that of the total distribution.

Finally, for each release, we then identified all corresponding operator overpasses. Because the number of overpasses varies per release, operators were provided with time-averaged flow rates for close to but not exactly half of their all overpasses. Figure S16 depicts a sample release distribution for all spectroscopy-based aircrafts (including MethaneAIR, who did not participate in Stage 3 analysis).

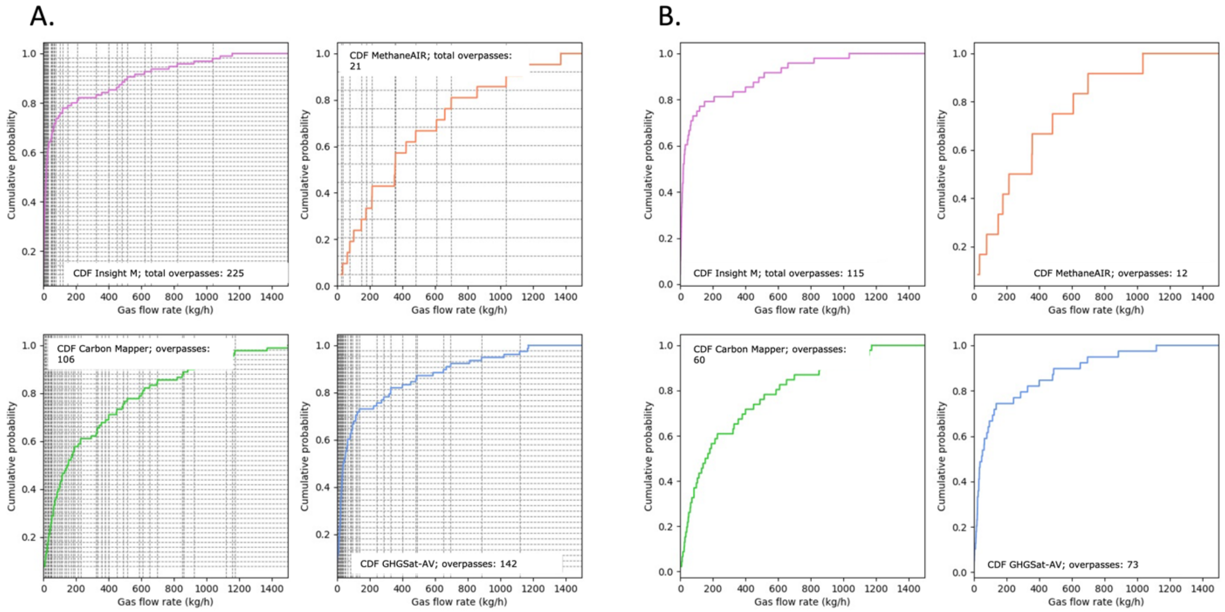

Figure S17 Cumulative probability distribution each operator (including MethaneAIR, who did not participate in Stage 3 of unblinding). We selected a number of points on the distribution function equal to half the total number of releases conducted for the operator. We then determined the corresponding release rate, and provide operators with releases representative of their total distribution. Panel A shows the distribution of all releases. Panel B shows the distribution of releases selected for unblinding in Stage 3.

2 Supplementary Results

2.1 Daily average windspeed

Average windspeed (meters / second) for each day of aircraft testing. Windspeeds are lower than those reported in Rutherford et al., 2023. Lower windspeeds would make testing conditions more difficult for imaging based technologies, due to potential pooling of methane onsite. This is consistent with reports from teams tested.

**Date**	**Operators Tested**	**Average Windspeed During Testing Period**
2022-Oct-10	Carbon Mapper	1.56 m/s
2022-Oct-11	Carbon Mapper	2.15 m/s
2022-Oct-12	Carbon Mapper	1.35 m/s
2022-Oct-24	Insight M	3.48 m/s
2022-Oct-25	Insight M, MethaneAIR	1.20 m/s
2022-Oct-26	Insight M	1.63 m/s
2022-Oct-27	Insight M	4.05 m/s
2022-Oct-28	Carbon Mapper, Insight M	2.67 m/s
2022-Oct-29	Carbon Mapper, MethaneAIR	1.58 m/s
2022-Oct-31	Carbon Mapper, GHGSat-AV	1.79 m/s
2022-Nov-02	GHGSat-AV	3.46 m/s

2022-Nov-04	GHGSat-AV	1.53 m/s
2022-Nov-07	GHGSat-AV	3.66 m/s
2022-Nov-08	Scientific Aviation	5.71 m/s
2022-Nov-10	Scientific Aviation	3.37 m/s
2022-Nov-11	Scientific Aviation	3.95 m/s

Table S17: Daily average windspeeds as measured onsite by the Stanford team during periods of aircraft testing

2.2 Aircraft data reporting

All aircraft were requested to use typical data collection and analysis procedures to best represent real world operations, including for any quality control filtering. Several operators raised concerns regarding stagnant wind conditions onsite during testing. Table S17 summarizes the timeline for reporting results for all three stages.

Table S18: Operator data reporting timeline

**Operator**	**Testing Complete**	**Stage 1 Submitted**	**Start Stage 2**	**Stage 2 Submitted**	**Start Stage 3**	**Stage 3 Submitted**
Carbon Mapper	10/31/22	01/03/23	01/11/23	2023-02-13	02/15/23	02/28/23
GHGSat	11/07/22	11/21/22	12/22/22	12/23/22	02/15/23	02/17/23
Insight M	10/28/22	11/17/22	12/19/22	12/20/22	02/15/23	02/23/23
Methane Air	10/29/22	03/22/23	NA	NA	NA	NA
Scientific Aviation	11/11/22	02/21/23	NA	NA	NA	NA

2.2.1 Carbon Mapper

Carbon Mapper apply filtering criteria to determine if a measurement was high enough quality for quantification. Prior to submitting any results, Carbon Mapper requested the ability to change their filtering with each stage of data submission, based on new data provided during unblinding. For full transparency, Carbon Mapper included quantification estimates for all measurements in each stage, including those that failed their filtering criteria. These are available in the original operator data reports. In Stage 2, unblinded wind data did not change the quality control filtering of any measurements. However, in Stage 3, two measurements filtered from both Stages 1 and 2 passed the filtering criteria and were included in quantification estimates.

All Stanford analysis only includes measurements that pass the Carbon Mapper filtering. As a result, the results classification histogram and probability of detection plots in the main text detection capabilities for measurements that Carbon Mapper has confidence in *quantifying*. We use this approach for consistency when comparing with other operators, who did not distinguish if their filtering applied different to detection vs. quantification.

2.2.2 Insight M

To determine variability in their measurement instruments, Insight M flew with two measurement units during testing, one on each wing. They submitted two separate data reports, one for each measurement unit. In the main text, we consider the detection and quantification performance of the entire deployed system, rather than reporting results for each unit individually. If either unit detected an emission, we consider that a detection. We report quantification estimates using the average of two reported units. If one unit reported values and the other did not, either due to a non-detect or QC filtering, we use the value for the reporting unit. Individual analysis for each measurement unit is included in Supplementary Results.

2.2.3 MethaneAIR

MethaneAIR uses two methods for analysis, described previously in Chulakadabba et al. (2023): divergence integral (DI) and modified integrated mass enhancement (mIME). In their main unblinded results submission, they report the average value of the two quantification methods. Results for each independent method are included in Supplemental Results.

2.2.4 Scientific Aviation

Four of these measurements were all conducted on November 8th, when wind conditions were not conducive towards measurement. The Scientific Aviation flight team informed Stanford on Nov 8th after testing was completed that the measurements were affected by wind conditions that blew the plume towards extremely tall power lines which prevented them from using the ideal flight path. Two additional points were due to too few laps conducted in the field (on November 10th and 11th), and an additional measurement was discarded on November 11th because not enough of the plume near the ground surface was captured.

2.3 Additional analysis of operator-reported results

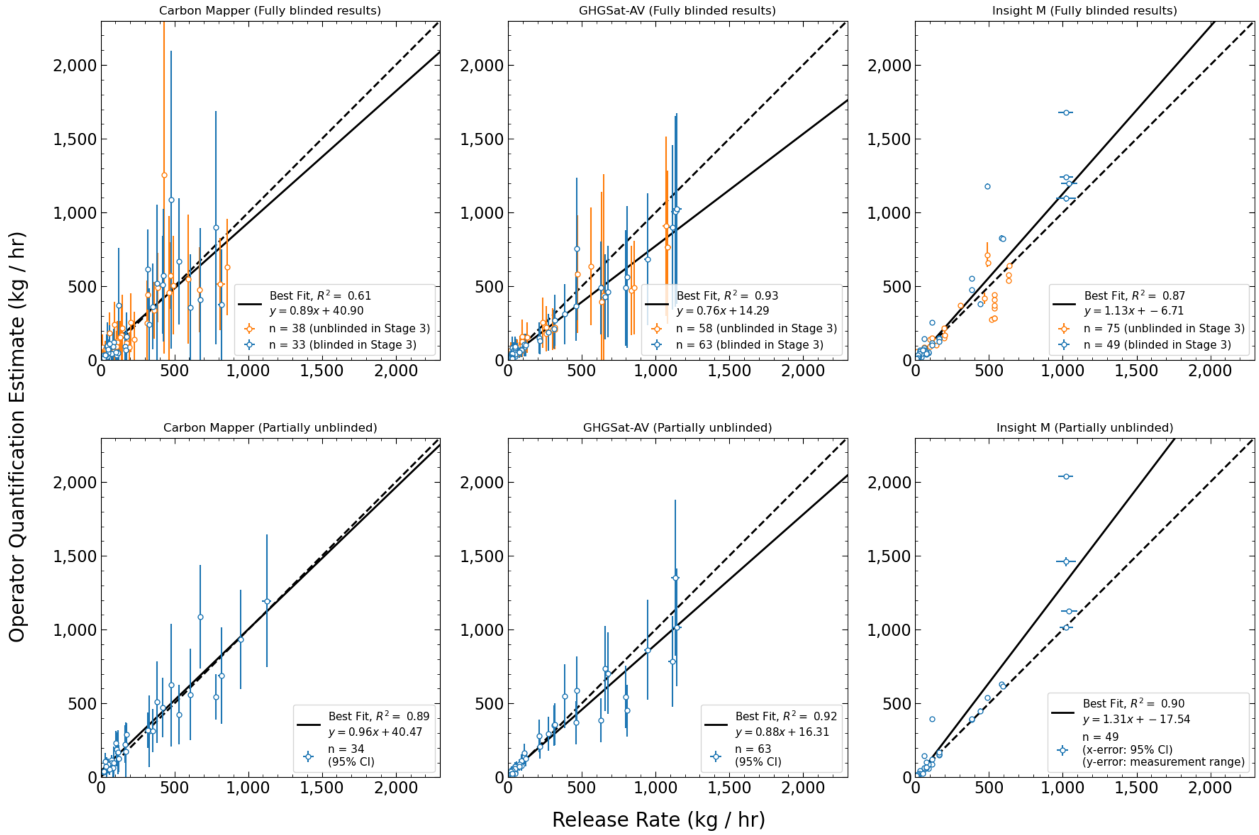

Figure S18: Quantification estimates for Stage 1 and Stage 3 results for Stage 3 participants. The top row shows quantification estimates that were submitted fully blinded, with color indicating which measurements were unblinded in Stage 3 (orange) and which remained blinded (blue). Note that Carbon Mapper re-added a measurement in Stage 3 that was previously removed during their internal quality control.

2.3.1 Quantification Accuracy: error profile and best-fit residuals

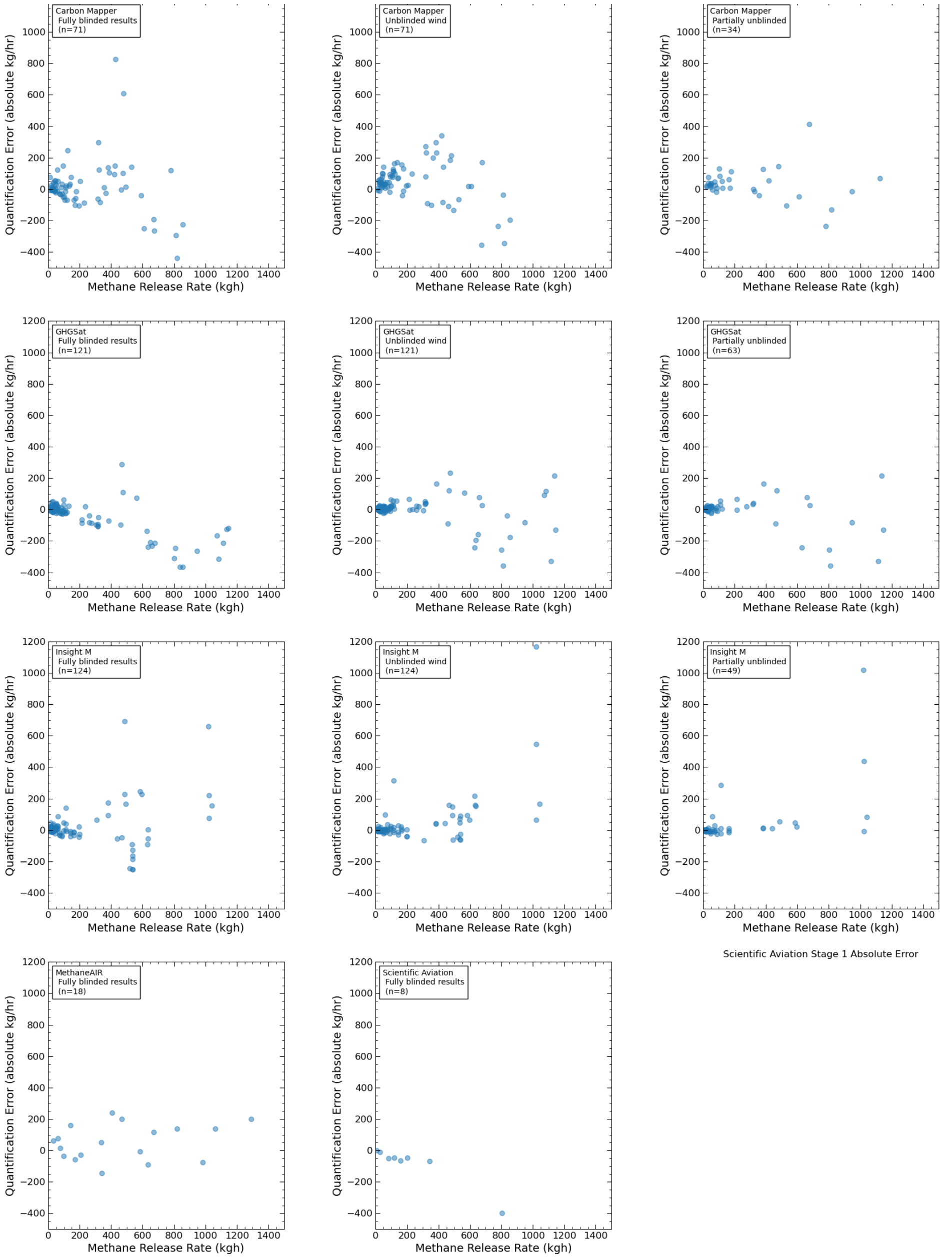

Figure S19: Absolute quantification error ($kg(CH_4)/hr$) for all points included in parity plots in the main text

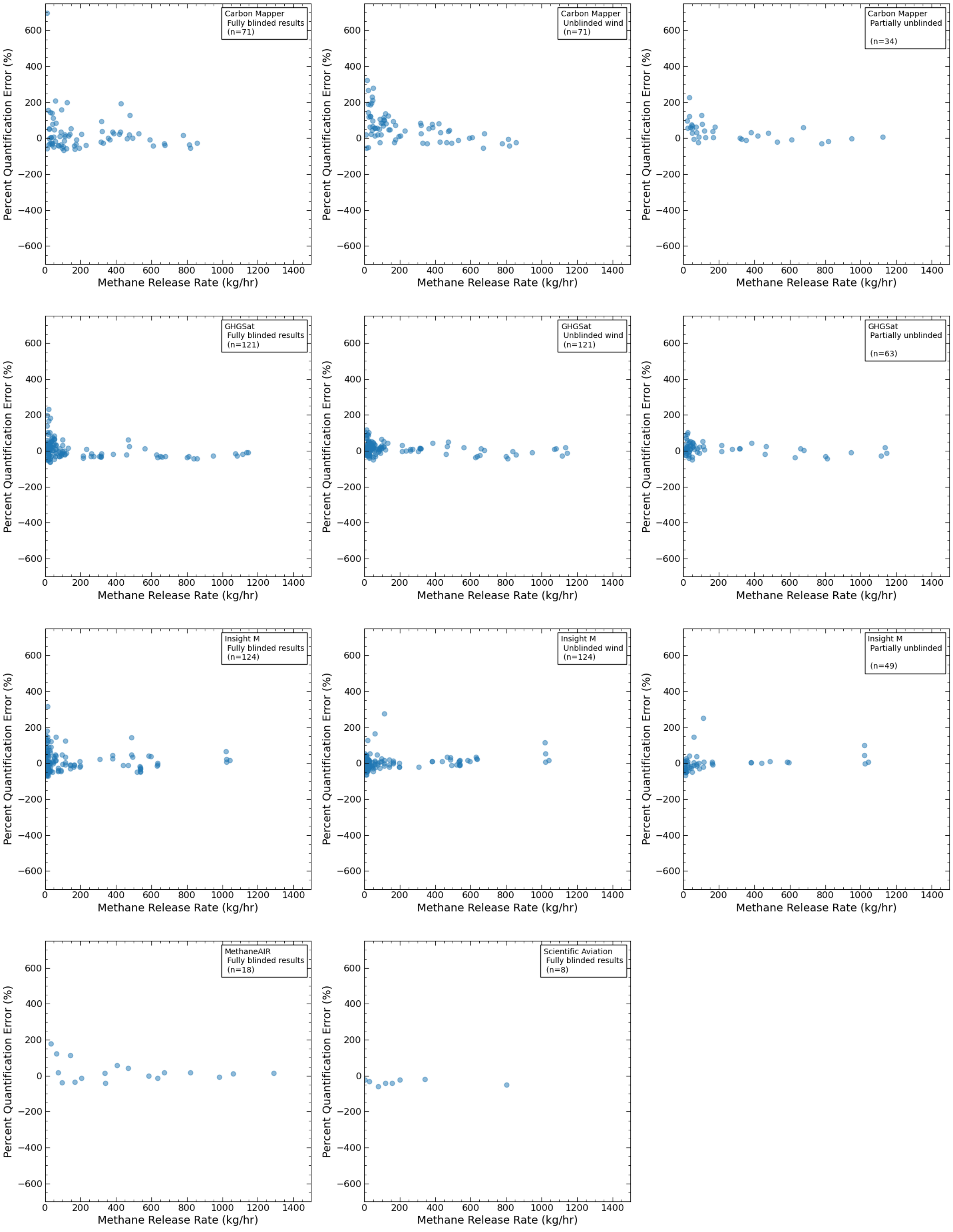

Figure S20: Percent quantification error for all points included in parity plots in the main text

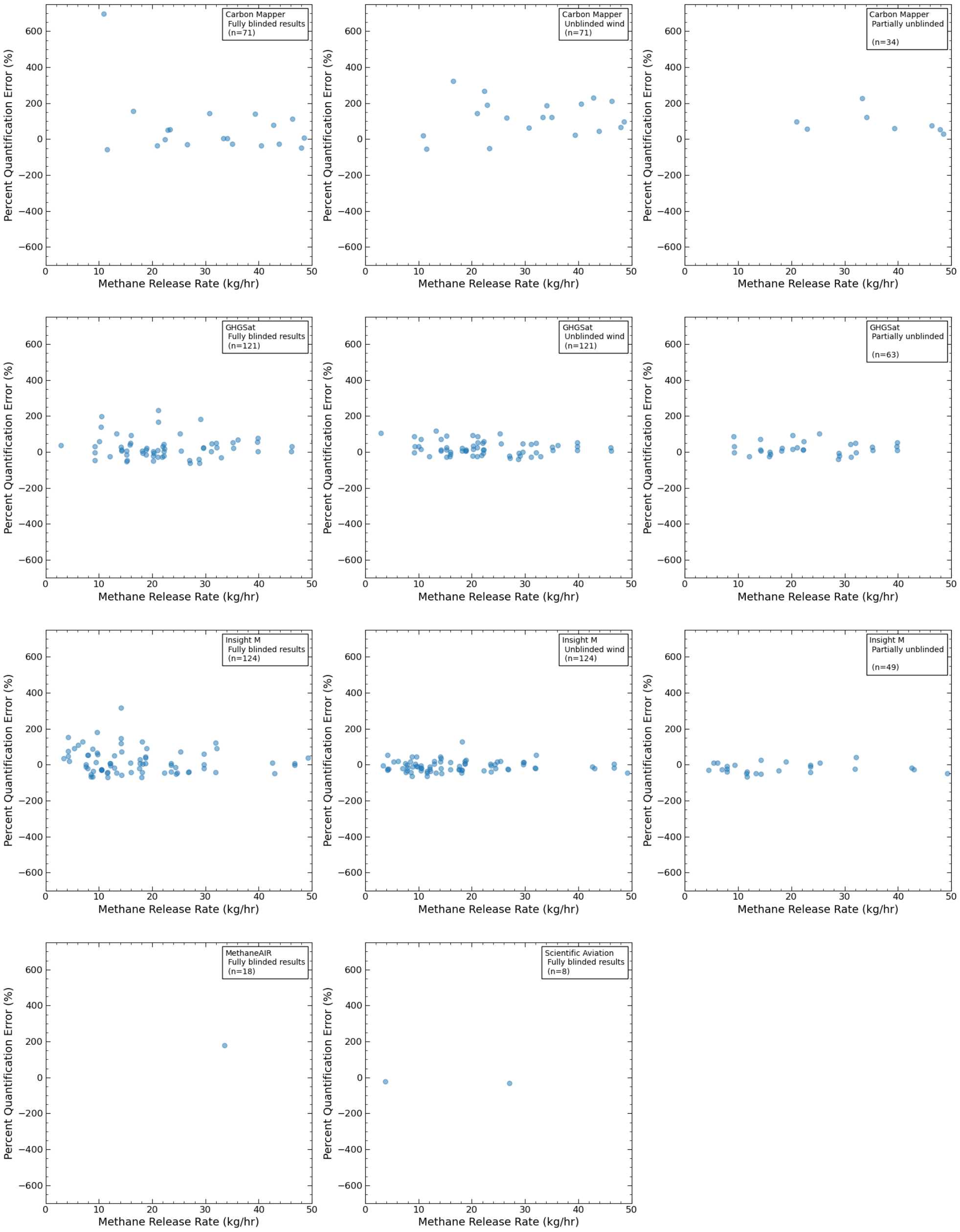

Figure S21 Percent quantification error for releases under 50 kg / hr . Axes are adjusted to provide greater clarity in visualization, value of n represents all data points including those beyond the range of the x-axis in this plot.

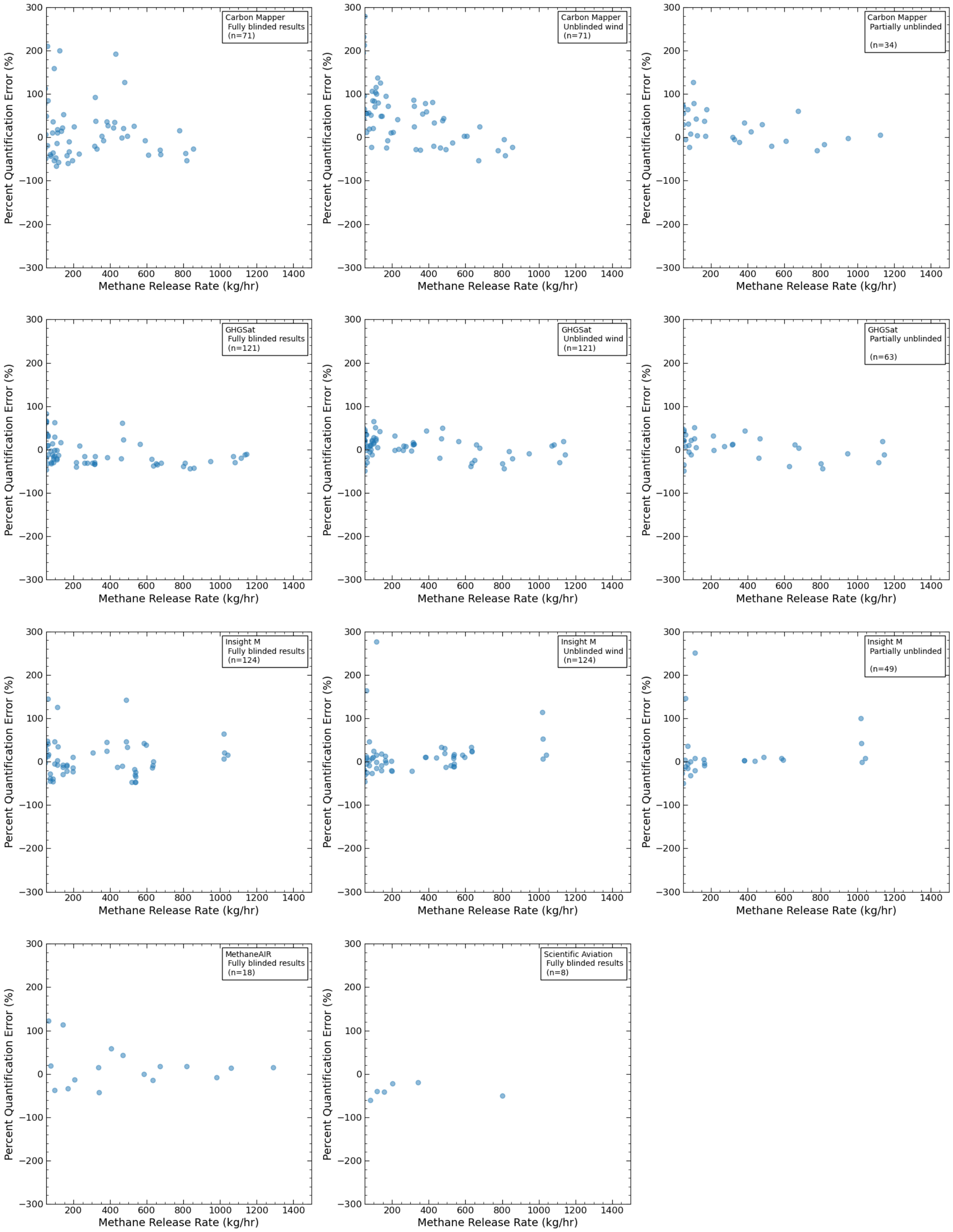

Figure S22 Percent quantification error for measurements greater than 50 kg / hr. Note y-axis is adjusted compared to previous percent quantification error for improved clarity. Value of n represents all data points including those beyond the range of the x-axis in this plot.

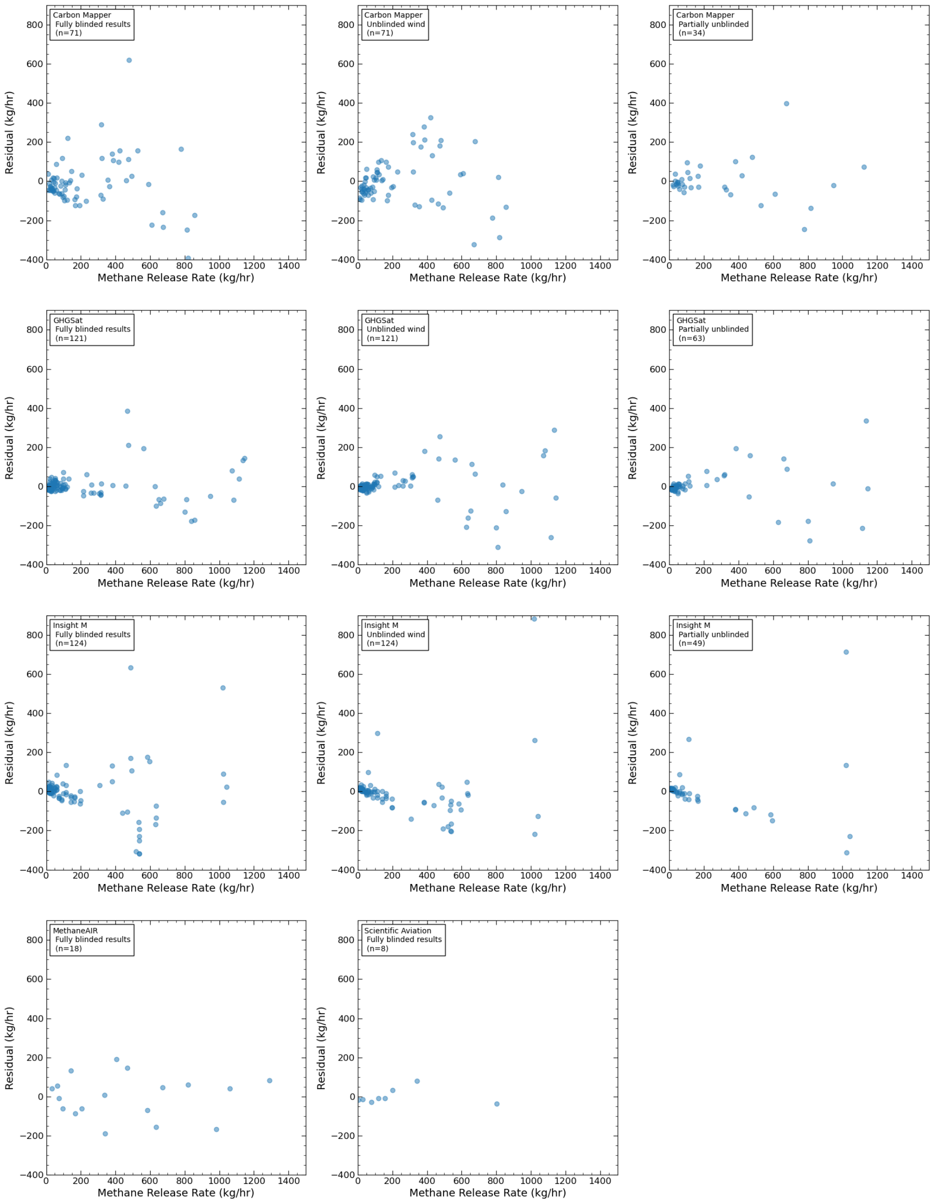

Figure S23: Residuals for best fit linear regression for all points included in parity plots in the main text

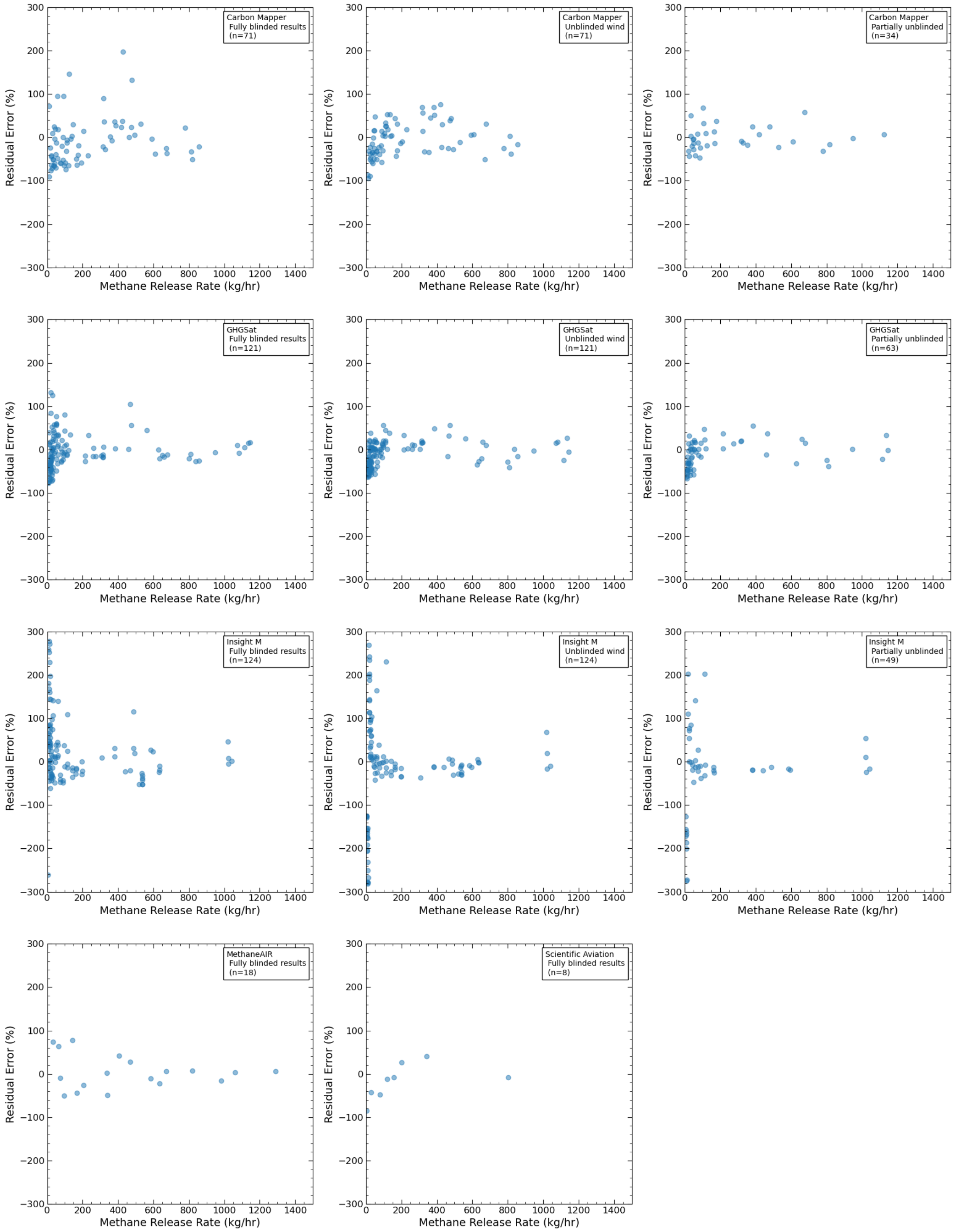

Figure S24 Percent error of residuals for linear best fit on all aircraft quantification estimates. Calculated as the difference between operator quantification estimate and linear best fit value, as a fraction of the linear best fit value.

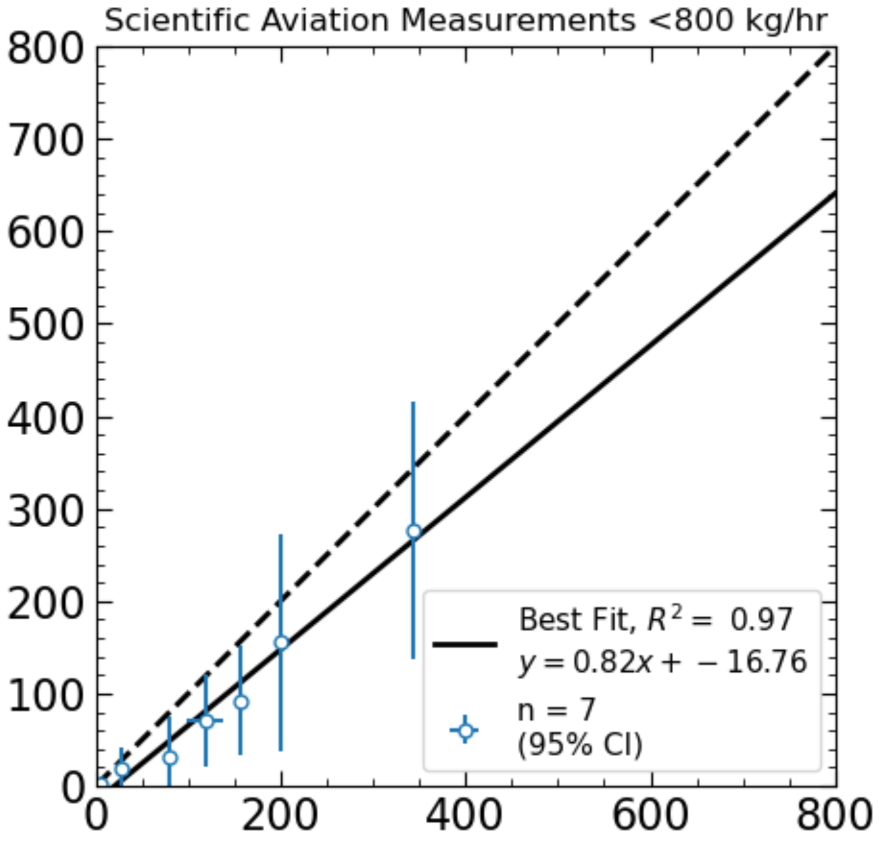

Figure S25 Scientific Aviation quantification plot excluding points release volumes greater than 800 kg CH_4 / hr. Note that slope increases to 0.82, compared to 0.52 with the excluded measurement above 800 kg CH_4 / hr, underscore the outsized effect of this point on slope and the importance of additional measurements to further characterize performance.

2.3.2 Insight M individual pod analysis

Insight M flew with two measurement pods, one attached to each wing and reported results for each pod individually. In the main manuscript, we averaged both values. Where one pod reported a non-zero release and the other did not (either reporting a zero, or filtering through quality control), we use the single reported value. Here, we use the individual reported pod values to generate the same figures reported in the main manuscript for quantification accuracy and lower detection limit.

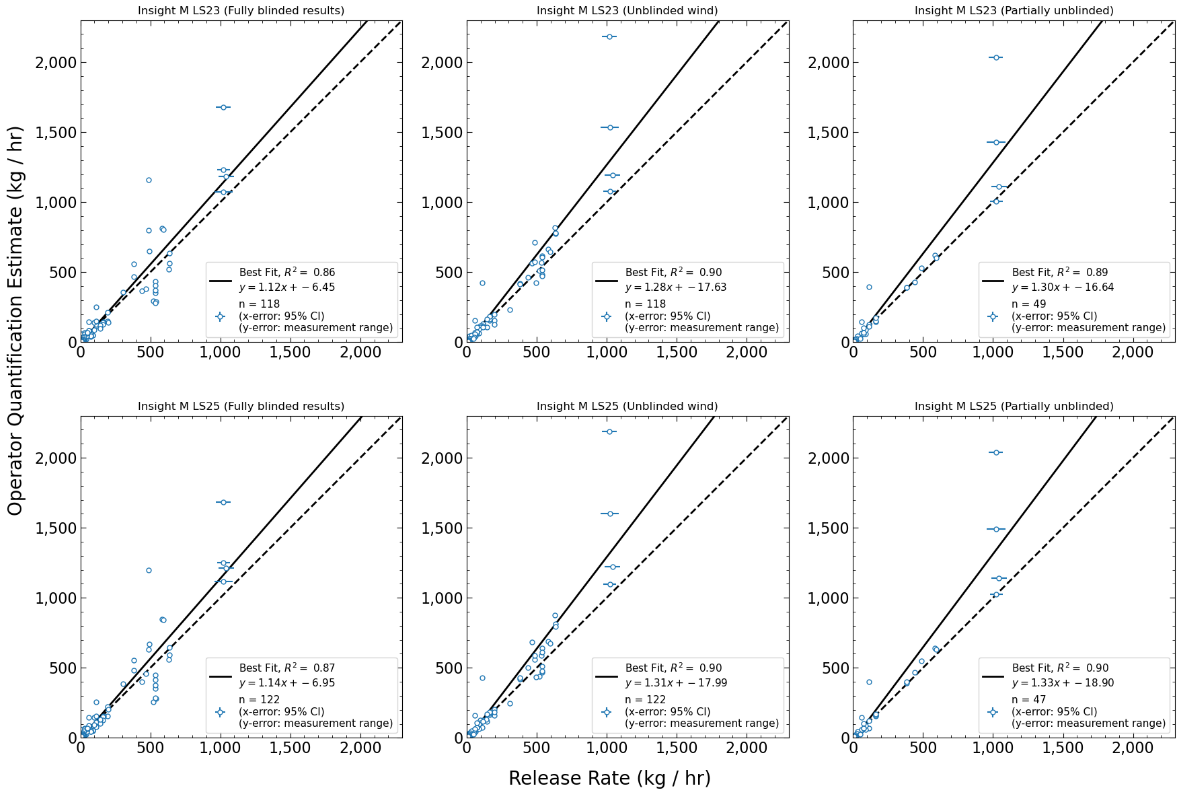

Figure S26 Quantification accuracy for Insight M individual pods LS23 and LS25

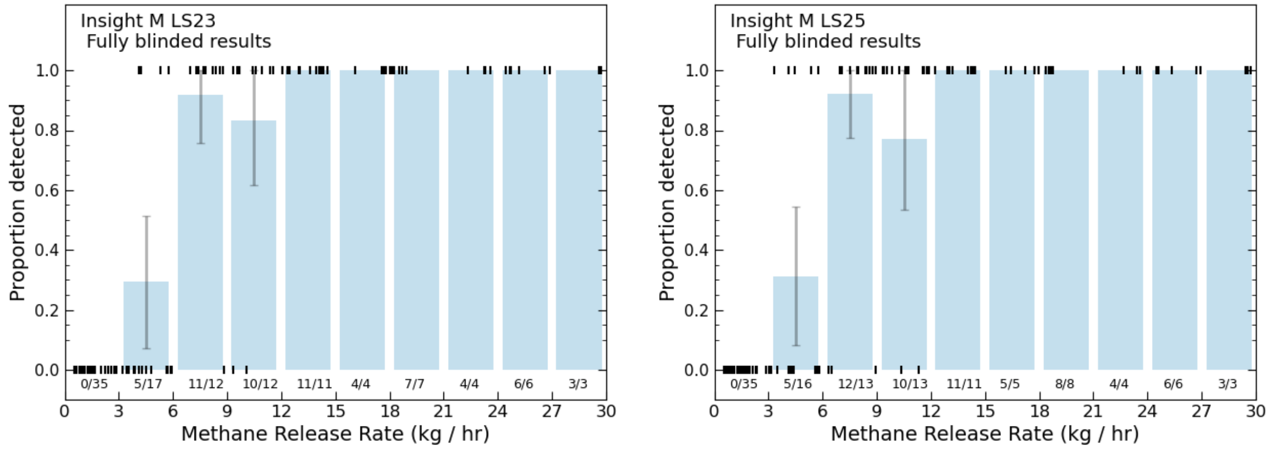

Figure S27 Insight M individual pods LS23 and LS25 probability of detection

2.3.3 Insight M wind normalized probability of detection

Insight M typically reports quantification estimates using wind-normalized flow rate, as in Sherwin, Chen et al., 2021. Here, we produce probability of detection plots using units of kg methane per hour per meter per second to allow for comparison with previous testing. In the Insight M operator results report, they include a column with the Dark Sky wind speed used in determining the quantification estimate. We divide their reported flow rate by the reported wind

speed to obtain the wind normalized flow rates reported in Figure S28. The left plot uses the same bins reported in Sherwin, Chen et al., 2021. In the flight configuration tested previously, Insight M did not detect the one release smaller than 5 kgh / mps, and only detected 1 of 14 releases between 5 – 10 kgh / mps, and 8 of 12 releases between 10 and 15 kgh / mps. In the current study, Insight M detected all releases above 1 kgh / mps.

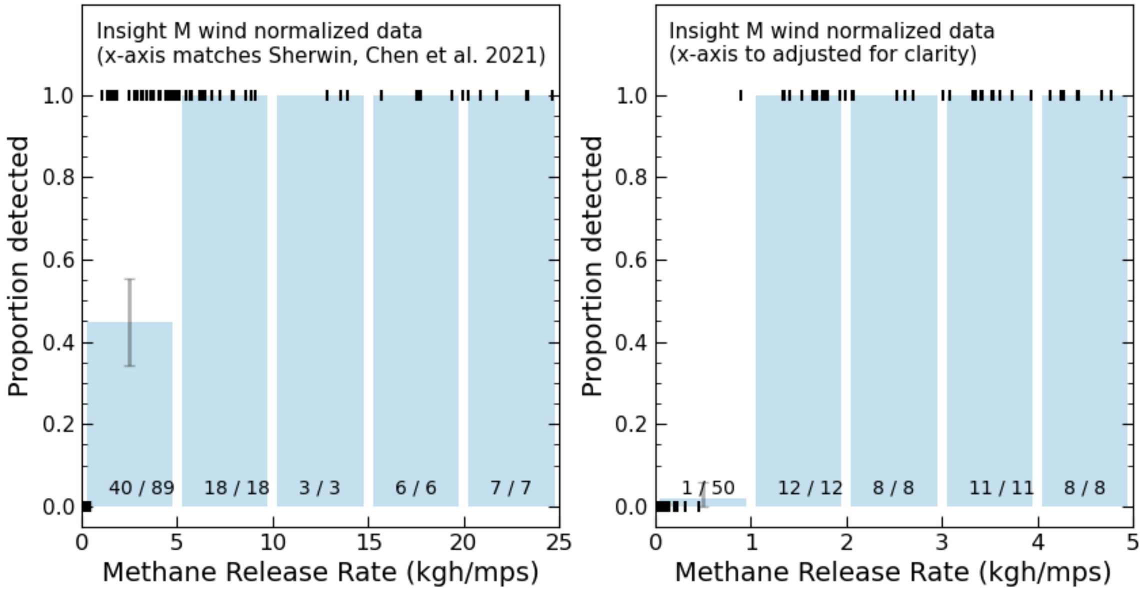

Figure S28 Insight M probability of detection using wind normalized methane flow rate, for comparison with Sherwin, Chen et al., 2021. Insight M reported wind speed from Dark Sky used in each quantification estimate in their Stage 1 report, and we use this as the input wind value for normalization. Figure on the left has x-axis that match Sherwin, Chen et al., 2021 for ready comparison between the two tests. Figure on the right shows all releases below 5 kgh/mps.

2.3.4 MethaneAIR Results for DI and mIME Methods

As described in the main text, MethaneAIR reported results that are the average of two different methodological approaches: divergence integral (DI) and modified methane mass enhancement (mIME). Here we provide parity plots for the individual methods. For each method, MethaneAIR reported 24 measurements. However, the mIME method detected two non-zero releases that were not detected using the DI method. Hence, the total number of points in Figure S29 differ by this amount.

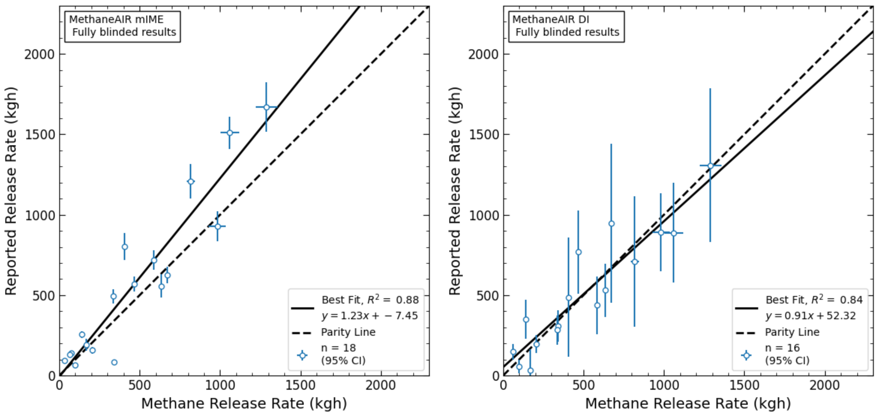

Figure S29: MethaneAIR quantification results for mIME and DI methods

3 References for Supplemental Material

Carbon Mapper. Carbon Mapper: accelerating local climate action, globally. https://carbonmapper.org (2023).

Chulakadabba, A.; Sargent, M.; Lauvaux, T.; Benmergui, J. S.; Franklin, J. E.; Chan Miller, C.; Wilzewski, J. S.; Roche, S.; Conway, E.; Souri, A. H.; Sun, K.; Luo, B.; Hawthrone, J.; Samra, J.; Daube, B. C.; Liu, X.; Chance, K.; Li, Y.; Gautam, R.; Omara, M.; Rutherford, J. S.; Sherwin, E. D.; Brandt, A.; Wofsy, S. C. Methane Point Source Quantification Using MethaneAIR: A New Airborne Imaging Spectrometer. *Atmospheric Meas. Tech.* **2023**, *16* (23), 5771–5785. https://doi.org/10.5194/amt-16-5771-2023

Conley, S. *et al.* Application of Gauss's theorem to quantify localized surface emissions from airborne measurements of wind and trace gases. *Atmos. Meas. Tech.* **10**, 3345–3358 (2017).

Duren, R. M. *et al.* California's methane super-emitters. *Nature* **575**, 180–184 (2019).

Esparza, Á. E. *et al.* Analysis of a tiered top-down approach using satellite and aircraft platforms to monitor oil and gas facilities in the Permian basin. *Renewable and Sustainable Energy Reviews* **178**, 113265 (2023).

Insight M. *Insight M*. https://www.insightm.com/ (2024).

Kuva Systems. Continuous Methane Monitoring with Actionable Images. https://www.kuvasystems.com/ (2023).

MethaneSAT. MethaneSAT: a better and faster way to track methane. https://www.methanesat.org/ (2023).

National Oceanic and Atmospheric Administration. Magnetic Declination Estimated Value. https://www.ngdc.noaa.gov/geomag/calculators/magcalc.shtml (2023).

Ravikumar, A. P. *et al.* Single-blind inter-comparison of methane detection technologies – results from the Stanford/EDF Mobile Monitoring Challenge. *Elementa: Science of the Anthropocene* **7**, 37 (2019).

Rutherford, J., Sherwin, E., Chen, Y., Aminfard, S. & Brandt, A. R. Evaluating methane emission quantification performance and uncertainty of aerial technologies via high-volume single-blind controlled releases. *Earth ArXiv* (2023) doi:https://doi.org/10.31223/X5KQ0X.

Scientific Aviation. Scientific Aviation. https://www.scientificaviation.com/ (2023).

Sherwin, E. D., Chen, Y., Ravikumar, A. P. & Brandt, A. R. Single-blind test of airplane-based hyperspectral methane detection via controlled releases. *Elementa: Science of the Anthropocene* **9**, (2021).

Sierra Instruments. Quadratherm 640i/780i Series Insertion and In-Line Mass Flow Meters Instruction Manual. https://www.sierrainstruments.com/userfiles/file/manuals/640i_780i_Instruction_Manual_v3.pdf (2014).

4 Appendix

4.1 Daily plume release definition plots

Here we present the plume definitions as determined using the algorithm described in SI Section S1.3.3. Plume definitions were used to determine which measurements to unblind in Stage 3.

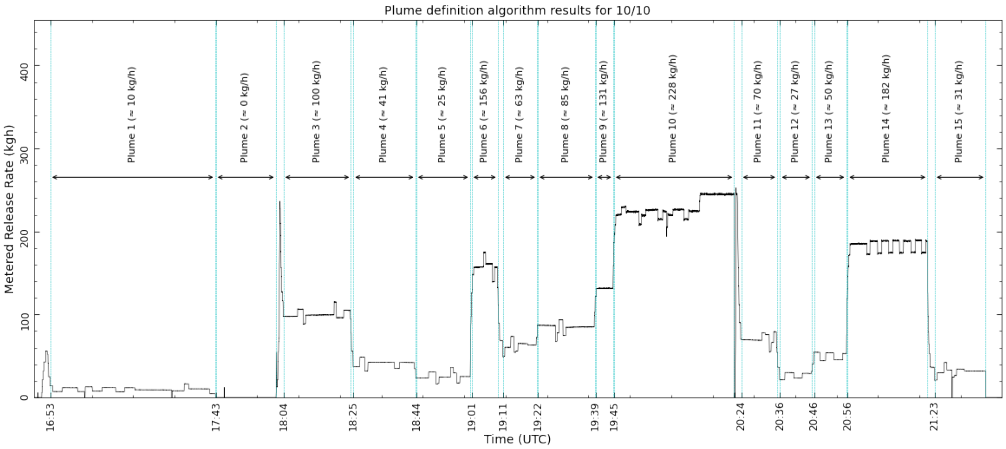

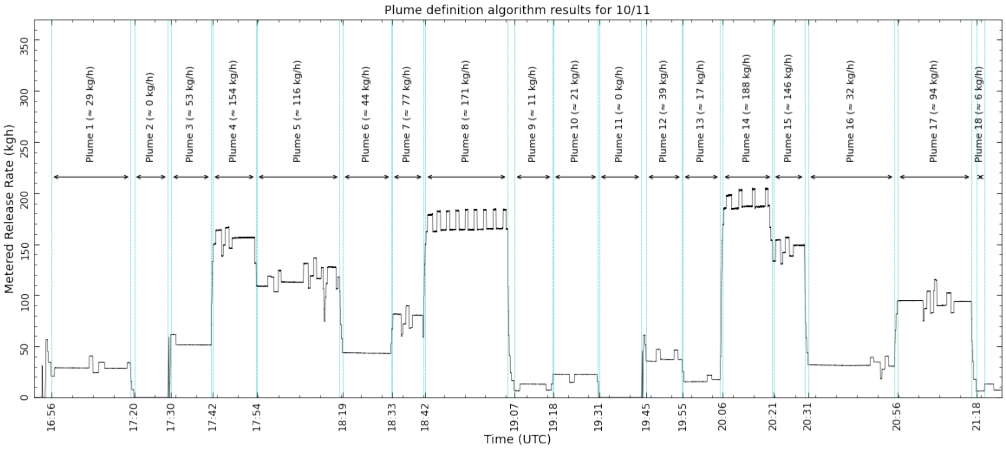
Plume definition algorithm results for 10/11
Metered Release Rate (kgh)
Time (UTC)
Plume 1 (≈ 29 kg/h)
Plume 2 (≈ 0 kg/h)
Plume 3 (≈ 53 kg/h)
Plume 4 (≈ 154 kg/h)
Plume 5 (≈ 116 kg/h)
Plume 6 (≈ 44 kg/h)
Plume 7 (≈ 77 kg/h)
Plume 8 (≈ 171 kg/h)
Plume 9 (≈ 11 kg/h)
Plume 10 (≈ 21 kg/h)
Plume 11 (≈ 0 kg/h)
Plume 12 (≈ 39 kg/h)
Plume 13 (≈ 17 kg/h)
Plume 14 (≈ 188 kg/h)
Plume 15 (≈ 146 kg/h)
Plume 16 (≈ 32 kg/h)
Plume 17 (≈ 94 kg/h)
Plume 18 (≈ 6 kg/h)
16:56 17:20 17:30 17:42 17:54 18:19 18:33 18:42 19:07 19:18 19:31 19:45 19:55 20:06 20:21 20:31 20:56 21:18

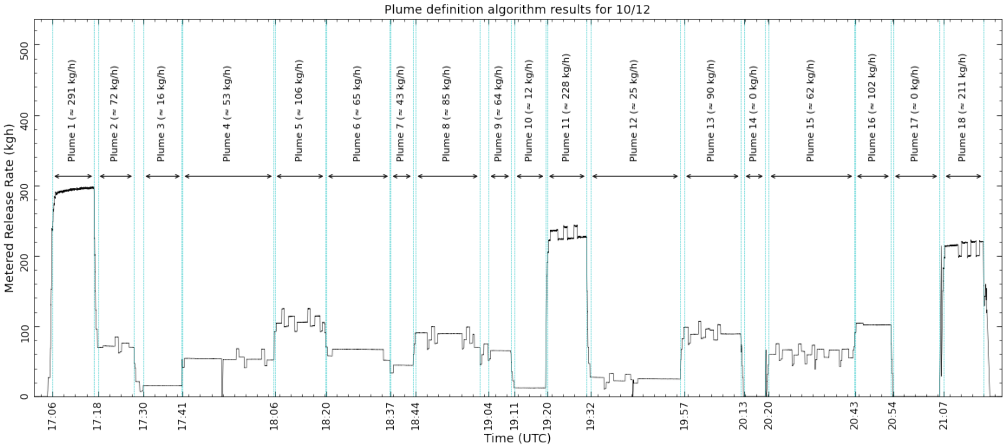
Plume definition algorithm results for 10/12
Metered Release Rate (kgh)
Time (UTC)
Plume 1 (≈ 291 kg/h)
Plume 2 (≈ 72 kg/h)
Plume 3 (≈ 16 kg/h)
Plume 4 (≈ 53 kg/h)
Plume 5 (≈ 106 kg/h)
Plume 6 (≈ 65 kg/h)
Plume 7 (≈ 43 kg/h)
Plume 8 (≈ 85 kg/h)
Plume 9 (≈ 64 kg/h)
Plume 10 (≈ 12 kg/h)
Plume 11 (≈ 228 kg/h)
Plume 12 (≈ 25 kg/h)
Plume 13 (≈ 90 kg/h)
Plume 14 (≈ 0 kg/h)
Plume 15 (≈ 62 kg/h)
Plume 16 (≈ 102 kg/h)
Plume 17 (≈ 0 kg/h)
Plume 18 (≈ 211 kg/h)
17:06 17:18 17:30 17:41 18:06 18:20 18:37 18:44 19:04 19:11 19:20 19:32 19:57 20:13 20:20 20:43 20:54 21:07

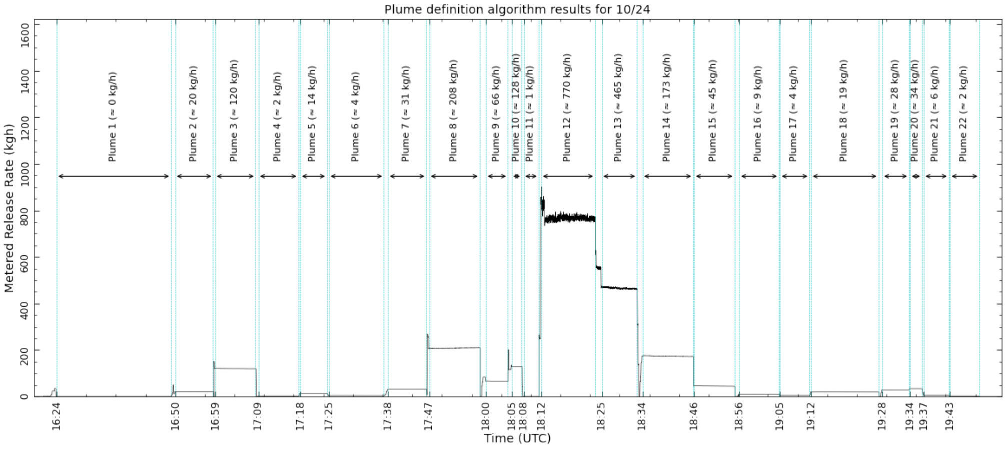
Plume definition algorithm results for 10/24
Metered Release Rate (kgh)
Time (UTC)
Plume 1 (≈ 0 kg/h)
Plume 2 (≈ 20 kg/h)
Plume 3 (≈ 120 kg/h)
Plume 4 (≈ 2 kg/h)
Plume 5 (≈ 14 kg/h)
Plume 6 (≈ 4 kg/h)
Plume 7 (≈ 31 kg/h)
Plume 8 (≈ 208 kg/h)
Plume 9 (≈ 66 kg/h)
Plume 10 (≈ 128 kg/h)
Plume 11 (≈ 1 kg/h)
Plume 12 (≈ 770 kg/h)
Plume 13 (≈ 465 kg/h)
Plume 14 (≈ 173 kg/h)
Plume 15 (≈ 45 kg/h)
Plume 16 (≈ 9 kg/h)
Plume 17 (≈ 4 kg/h)
Plume 18 (≈ 19 kg/h)
Plume 19 (≈ 28 kg/h)
Plume 20 (≈ 34 kg/h)
Plume 21 (≈ 6 kg/h)
Plume 22 (≈ 2 kg/h)
16:24 16:50 16:59 17:09 17:18 17:25 17:38 17:47 18:00 18:05 18:08 18:12 18:25 18:34 18:46 18:56 19:05 19:12 19:28 19:34 19:37 19:43

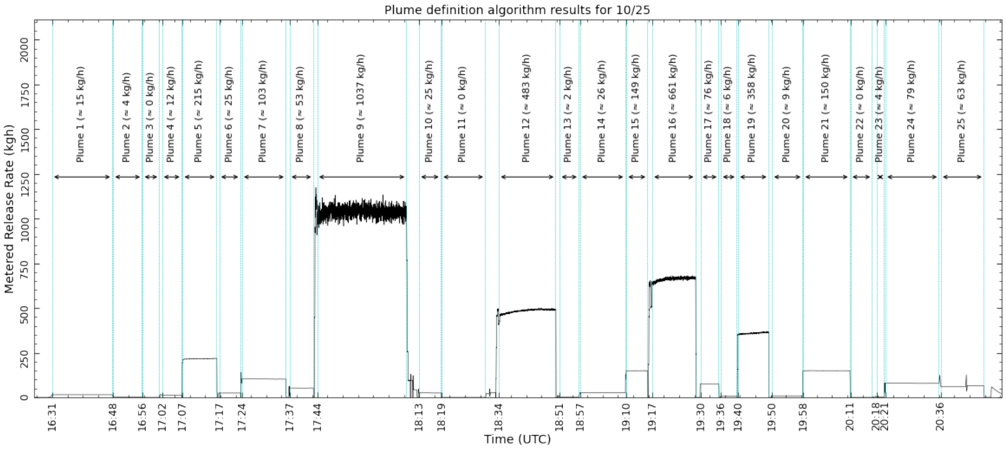
Plume definition algorithm results for 10/25
Metered Release Rate (kgh)
Time (UTC)

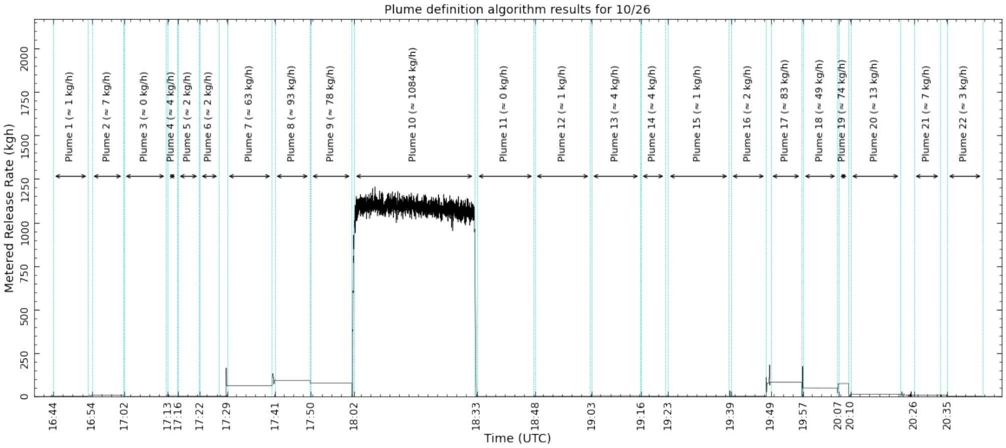
Plume definition algorithm results for 10/26
Metered Release Rate (kgh)
Time (UTC)

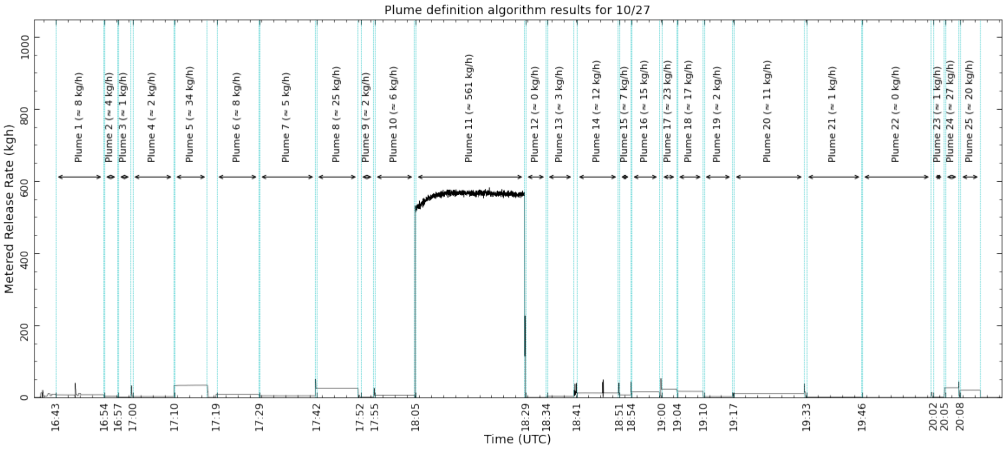
Plume definition algorithm results for 10/27
Metered Release Rate (kgh)
Time (UTC)

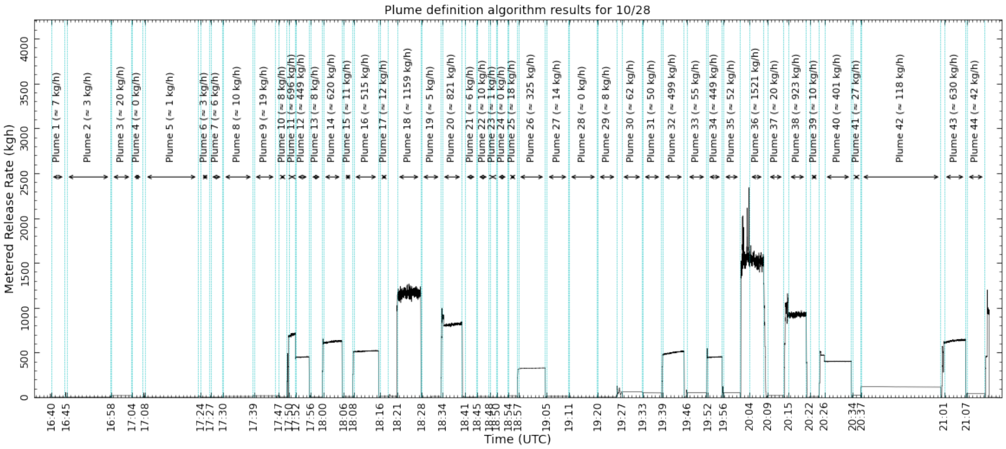
Plume definition algorithm results for 10/28
Metered Release Rate (kgh)
Time (UTC)

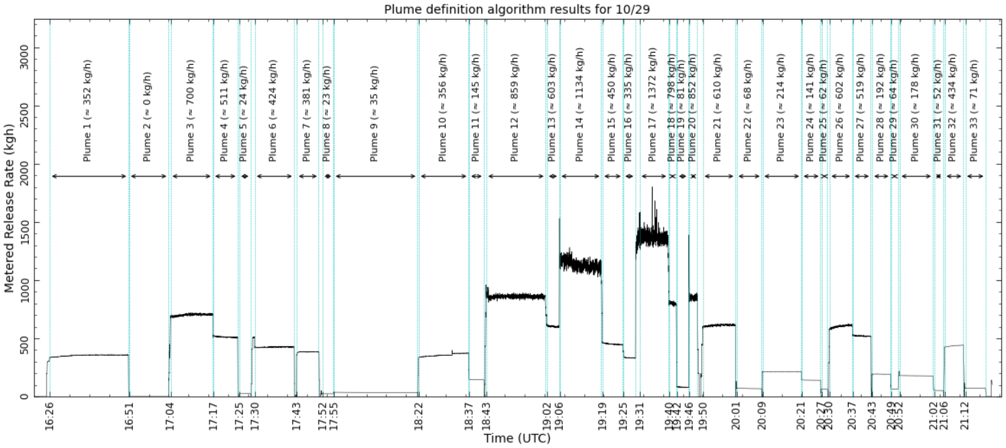
Plume definition algorithm results for 10/29
Metered Release Rate (kgh)
Time (UTC)

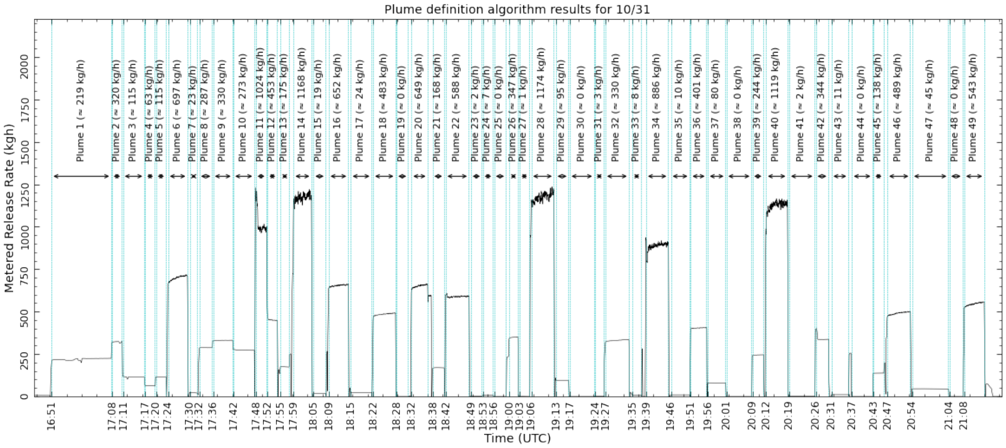
Plume definition algorithm results for 10/31
Metered Release Rate (kgh)
Time (UTC)

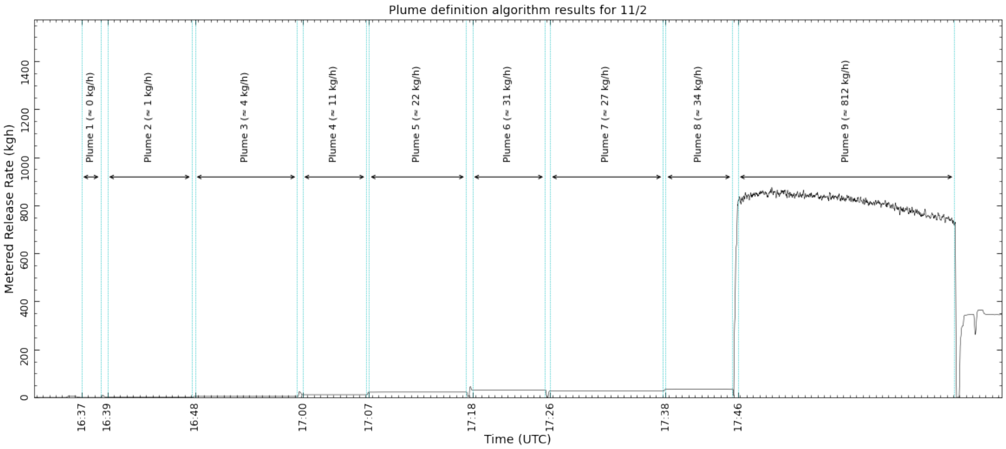
Plume definition algorithm results for 11/2
Metered Release Rate (kgh)
Time (UTC)
Plume 1 (≈ 0 kg/h)
Plume 2 (≈ 1 kg/h)
Plume 3 (≈ 4 kg/h)
Plume 4 (≈ 11 kg/h)
Plume 5 (≈ 22 kg/h)
Plume 6 (≈ 31 kg/h)
Plume 7 (≈ 27 kg/h)
Plume 8 (≈ 34 kg/h)
Plume 9 (≈ 812 kg/h)
16:37 16:39 16:48 17:00 17:07 17:18 17:26 17:38 17:46

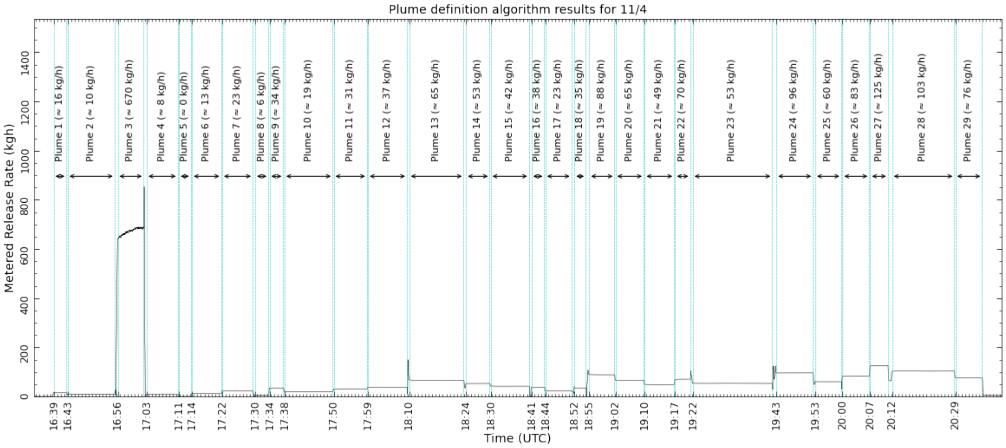
Plume definition algorithm results for 11/4
Metered Release Rate (kgh)
Time (UTC)
Plume 1 (≈ 16 kg/h)
Plume 2 (≈ 10 kg/h)
Plume 3 (≈ 670 kg/h)
Plume 4 (≈ 8 kg/h)
Plume 5 (≈ 0 kg/h)
Plume 6 (≈ 13 kg/h)
Plume 7 (≈ 23 kg/h)
Plume 8 (≈ 6 kg/h)
Plume 9 (≈ 34 kg/h)
Plume 10 (≈ 19 kg/h)
Plume 11 (≈ 31 kg/h)
Plume 12 (≈ 37 kg/h)
Plume 13 (≈ 65 kg/h)
Plume 14 (≈ 53 kg/h)
Plume 15 (≈ 42 kg/h)
Plume 16 (≈ 38 kg/h)
Plume 17 (≈ 23 kg/h)
Plume 18 (≈ 35 kg/h)
Plume 19 (≈ 88 kg/h)
Plume 20 (≈ 65 kg/h)
Plume 21 (≈ 49 kg/h)
Plume 22 (≈ 70 kg/h)
Plume 23 (≈ 53 kg/h)
Plume 24 (≈ 96 kg/h)
Plume 25 (≈ 60 kg/h)
Plume 26 (≈ 83 kg/h)
Plume 27 (≈ 125 kg/h)
Plume 28 (≈ 103 kg/h)
Plume 29 (≈ 76 kg/h)
16:39 16:43 16:56 17:03 17:11 17:14 17:22 17:30 17:34 17:38 17:50 17:59 18:10 18:24 18:30 18:41 18:44 18:52 18:55 19:02 19:10 19:17 19:22 19:43 19:53 20:00 20:07 20:12 20:29

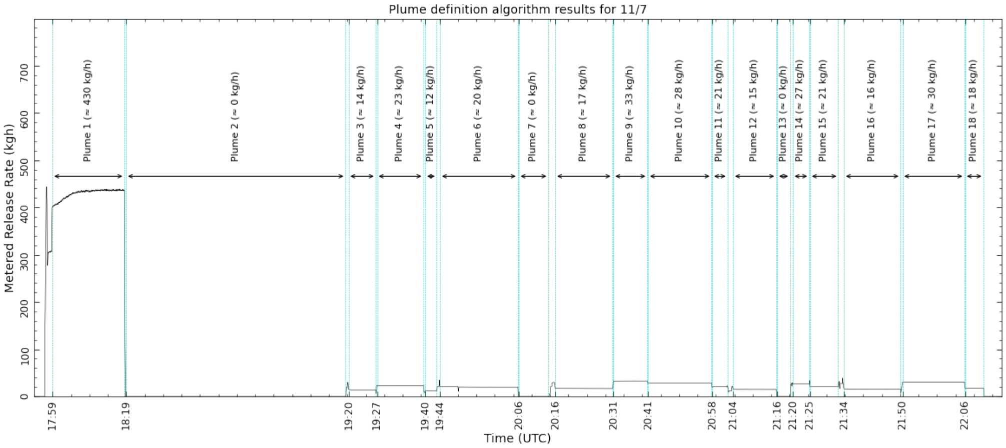
Plume definition algorithm results for 11/7
Metered Release Rate (kgh)
Time (UTC)
Plume 1 (≈ 430 kg/h)
Plume 2 (≈ 0 kg/h)
Plume 3 (≈ 14 kg/h)
Plume 4 (≈ 23 kg/h)
Plume 5 (≈ 12 kg/h)
Plume 6 (≈ 20 kg/h)
Plume 7 (≈ 0 kg/h)
Plume 8 (≈ 17 kg/h)
Plume 9 (≈ 33 kg/h)
Plume 10 (≈ 28 kg/h)
Plume 11 (≈ 21 kg/h)
Plume 12 (≈ 15 kg/h)
Plume 13 (≈ 0 kg/h)
Plume 14 (≈ 27 kg/h)
Plume 15 (≈ 21 kg/h)
Plume 16 (≈ 16 kg/h)
Plume 17 (≈ 30 kg/h)
Plume 18 (≈ 18 kg/h)
17:59 18:19 19:20 19:27 19:40 19:44 20:06 20:16 20:31 20:41 20:58 21:04 21:16 21:20 21:25 21:34 21:50 22:06

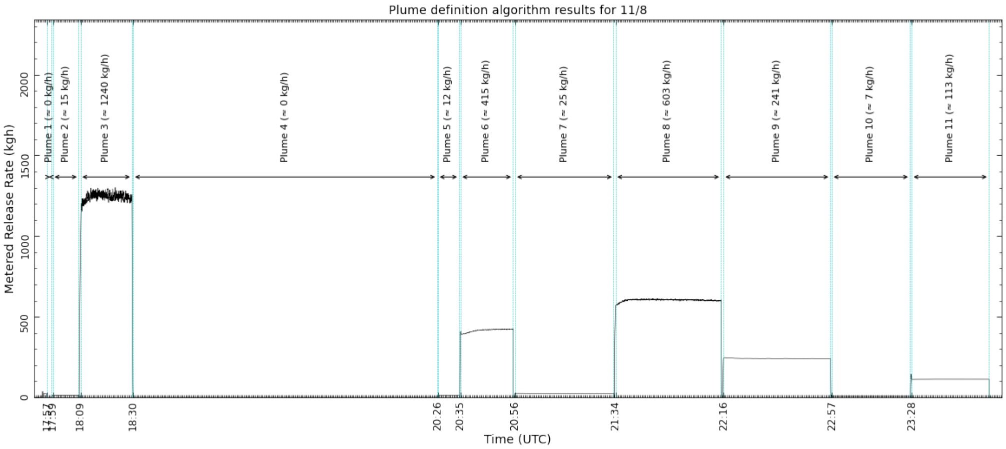

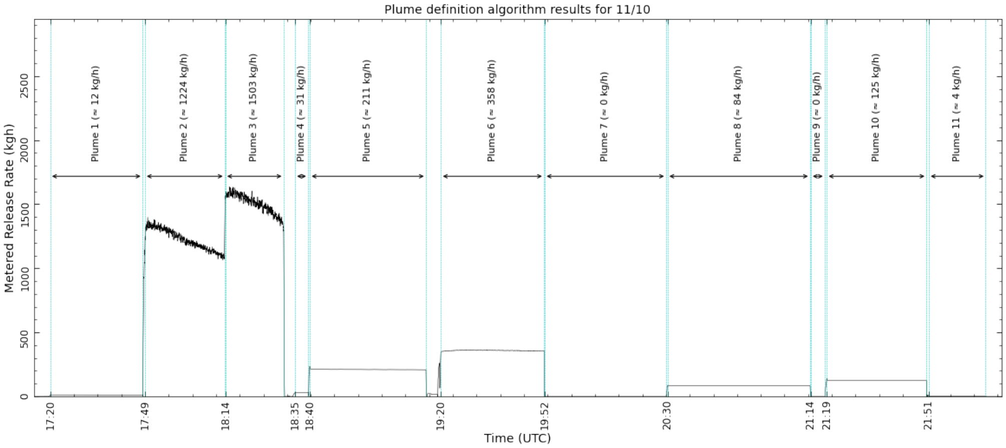

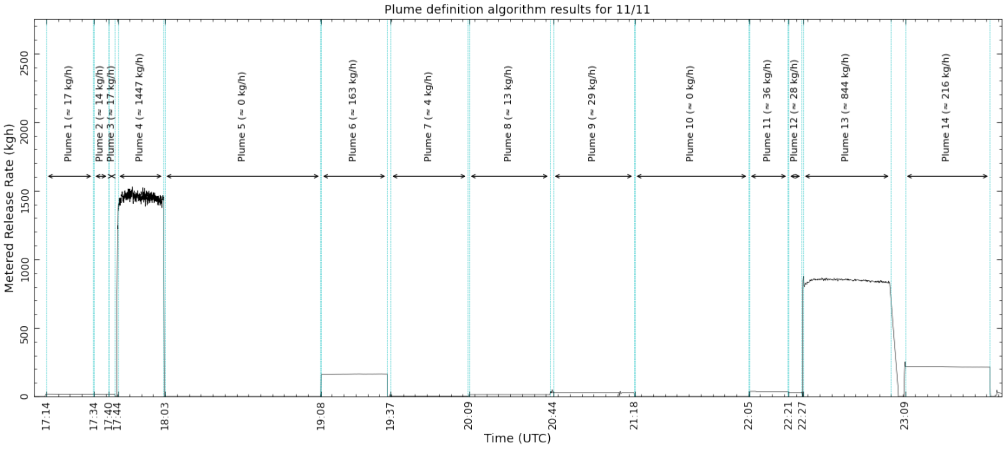

4.2 Daily Release Rates

Plots of daily release rates for each day of testing for each aircraft. Vertical lines represent a measurement, color indicates quality control filtering. For spectroscopy-based technologies, height of vertical lines represent average release rate for the 1-minute period prior to aircraft overpass.

4.2.1 Carbon Mapper

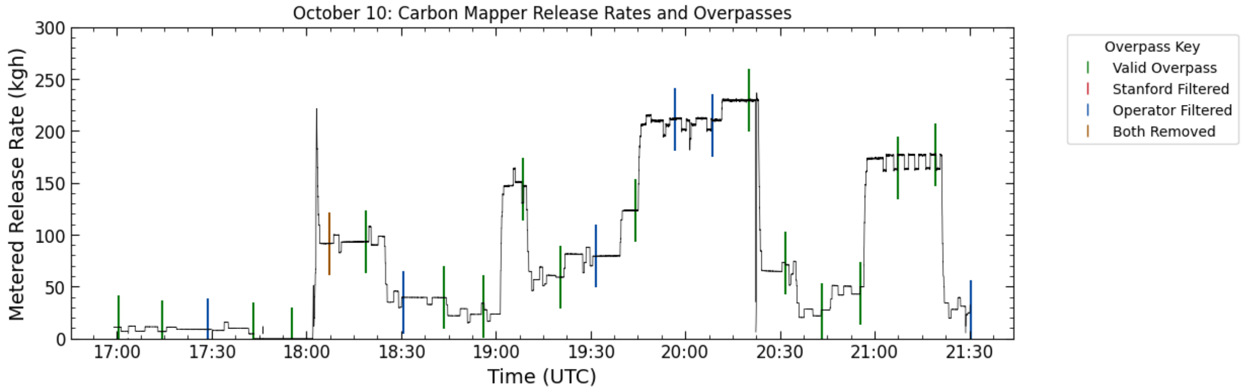

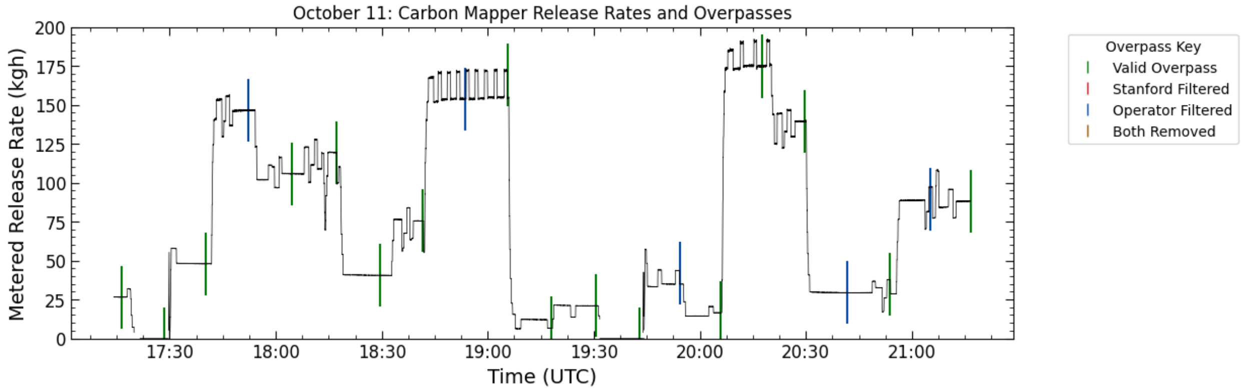

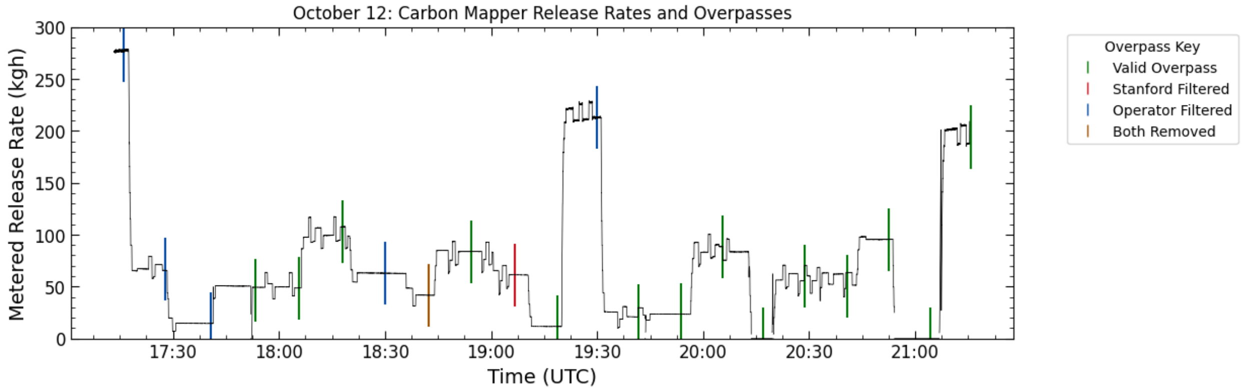

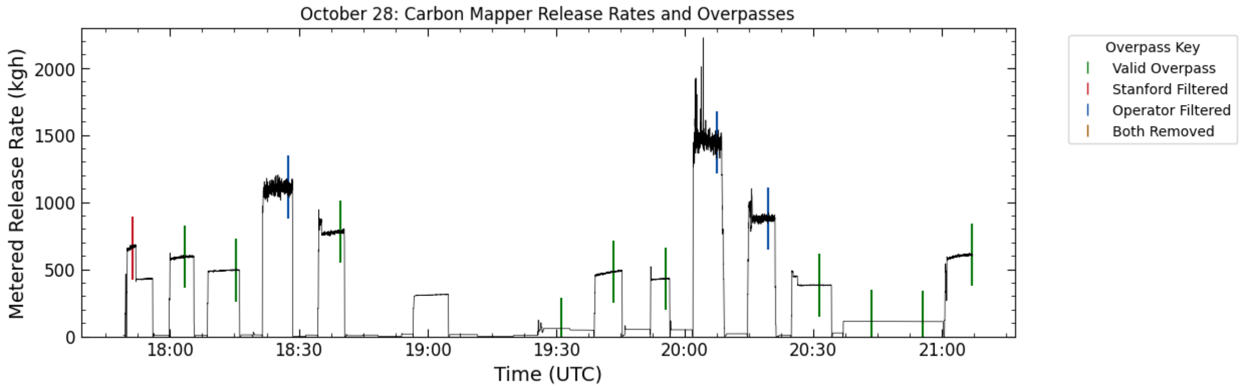

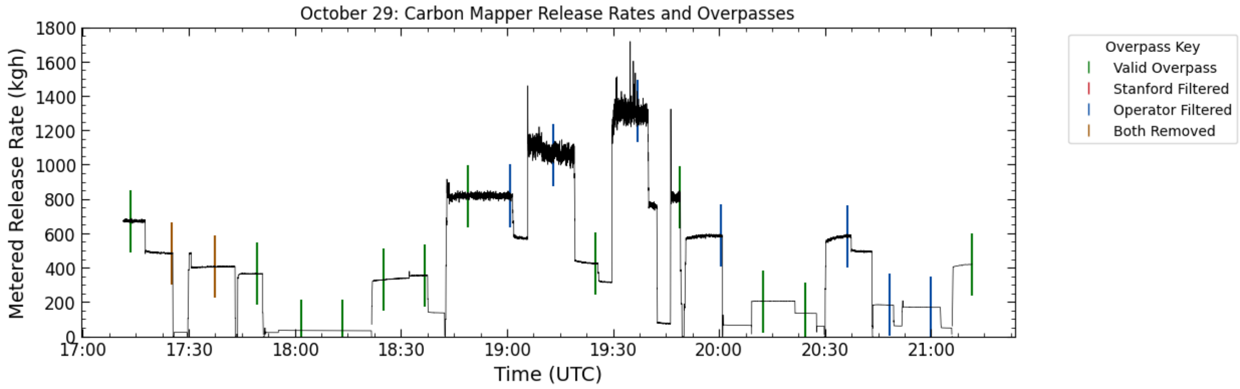

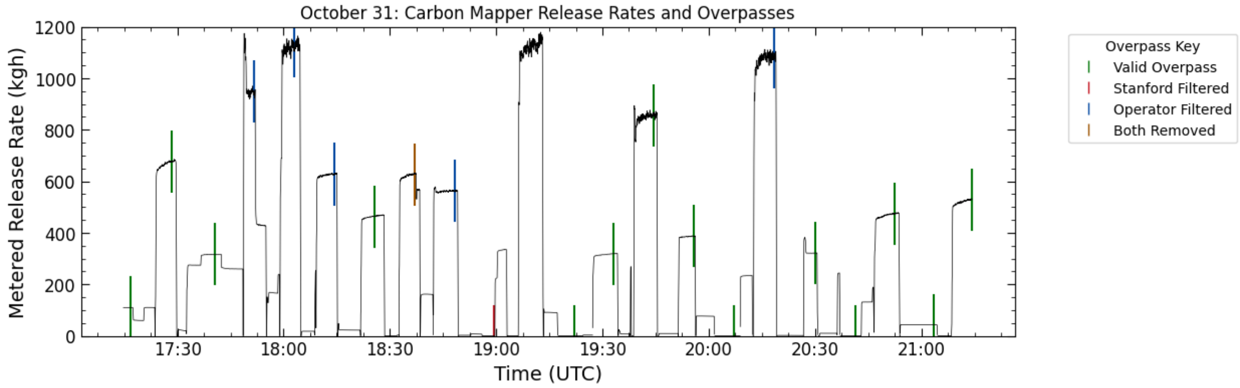

4.2.2 GHGSat-AV

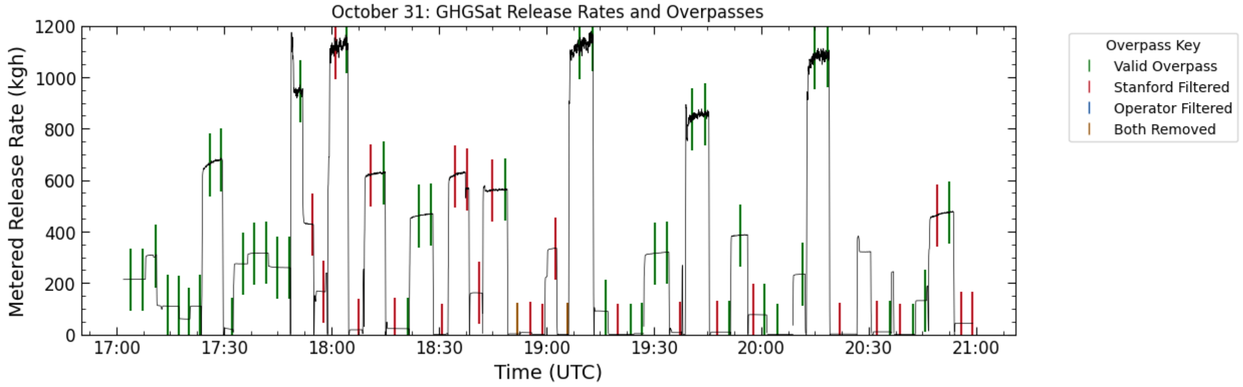
October 31: GHGSat Release Rates and Overpasses
Metered Release Rate (kgh)
Time (UTC)
Overpass Key
Valid Overpass
Stanford Filtered
Operator Filtered
Both Removed

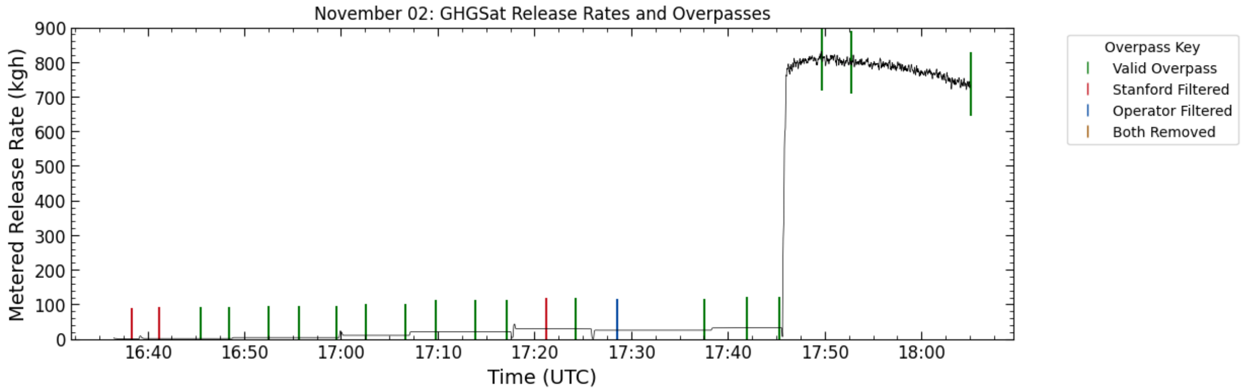
November 02: GHGSat Release Rates and Overpasses
Metered Release Rate (kgh)
Time (UTC)
Overpass Key
Valid Overpass
Stanford Filtered
Operator Filtered
Both Removed

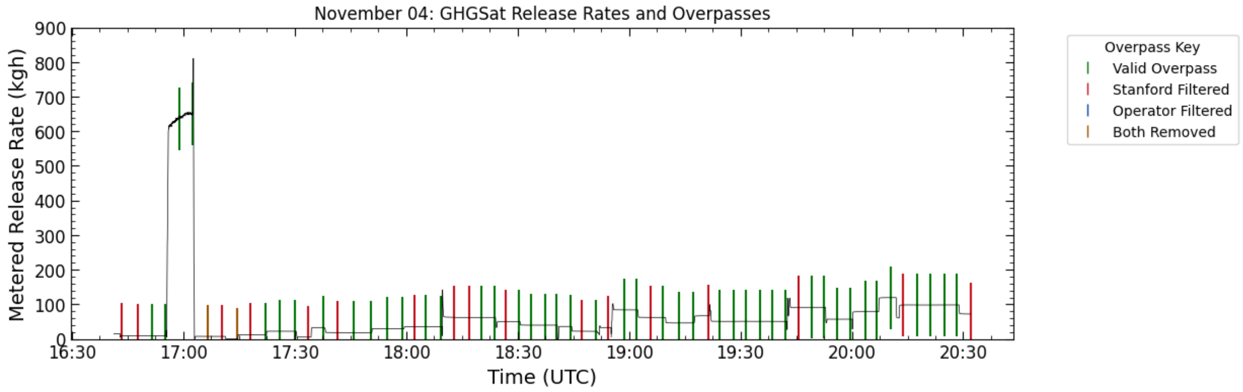
November 04: GHGSat Release Rates and Overpasses
Metered Release Rate (kgh)
Time (UTC)
Overpass Key
Valid Overpass
Stanford Filtered
Operator Filtered
Both Removed

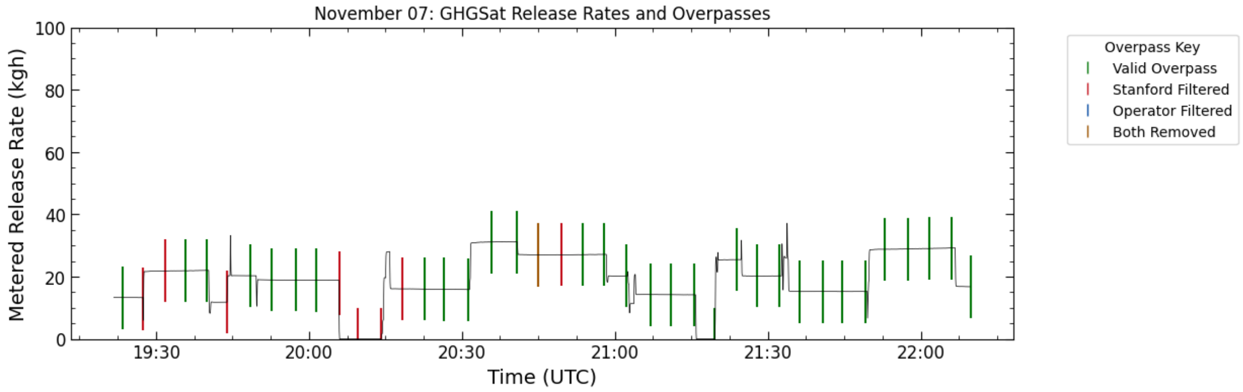
November 07: GHGSat Release Rates and Overpasses
Metered Release Rate (kgh)
Time (UTC)
Overpass Key
Valid Overpass
Stanford Filtered
Operator Filtered
Both Removed

4.2.3 Insight M

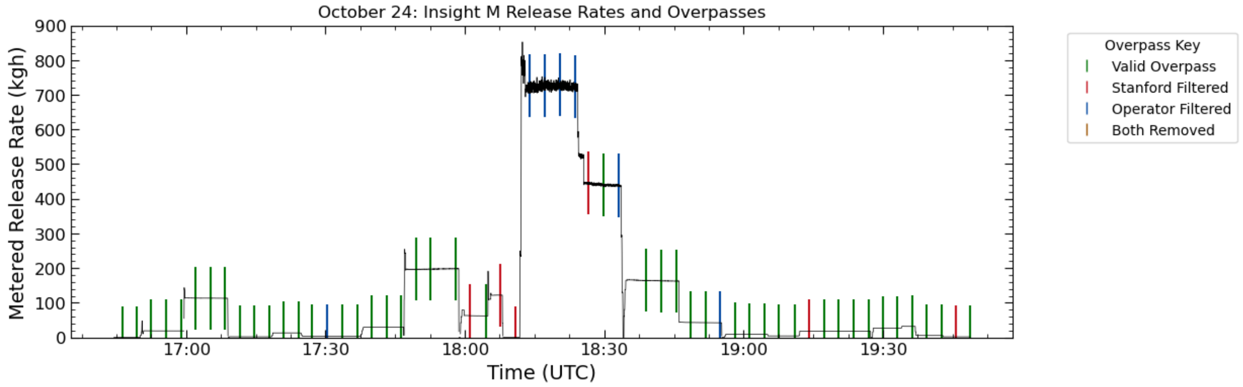

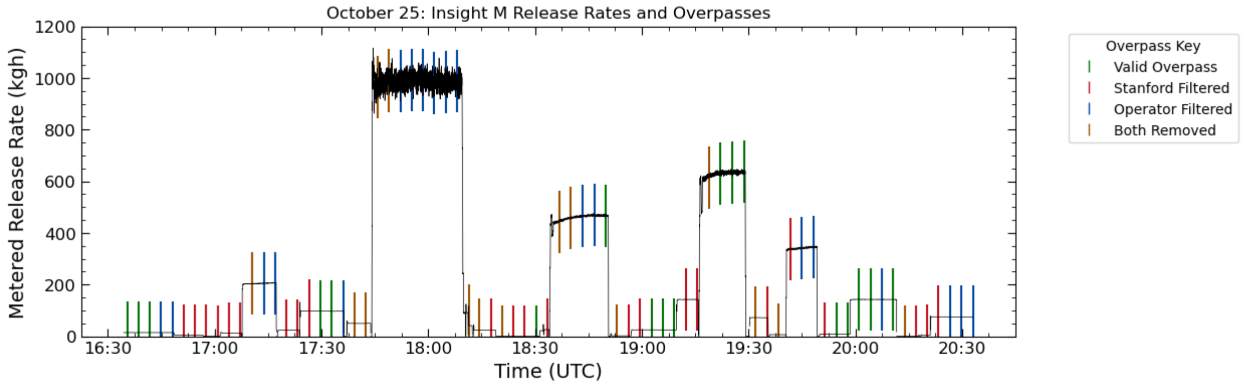

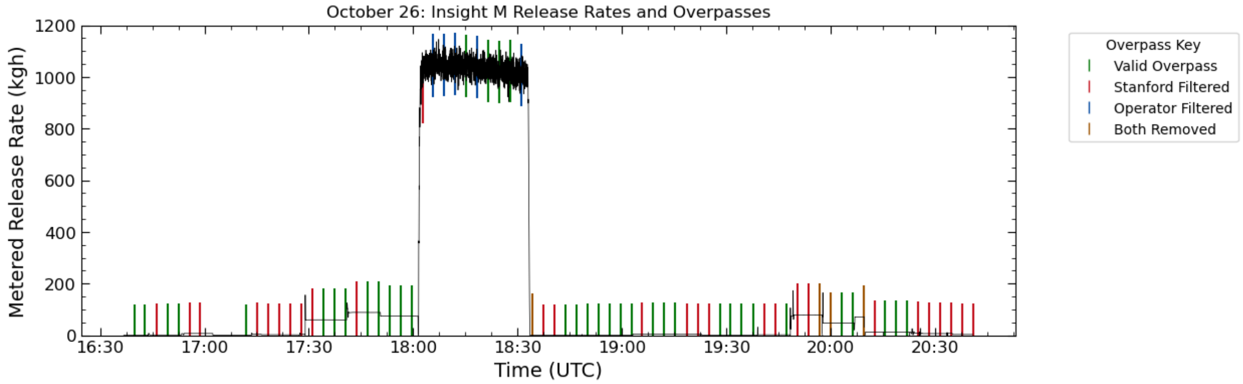

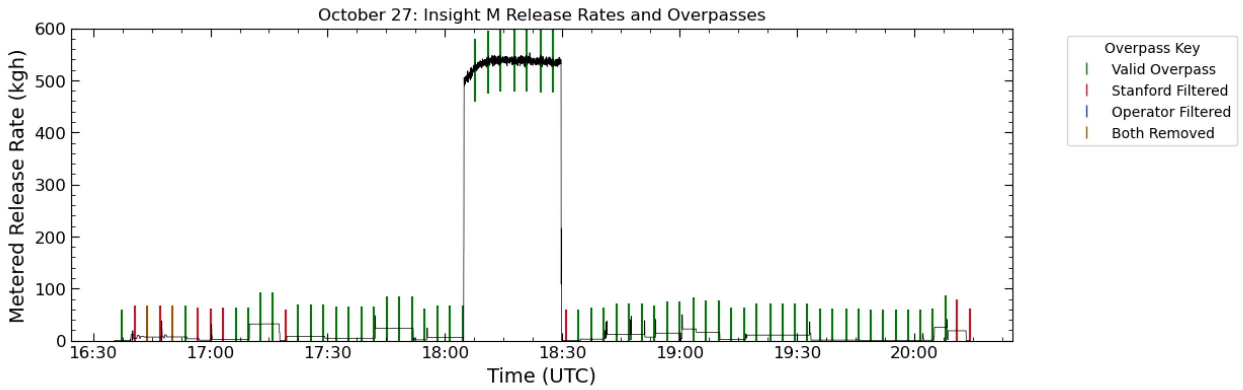

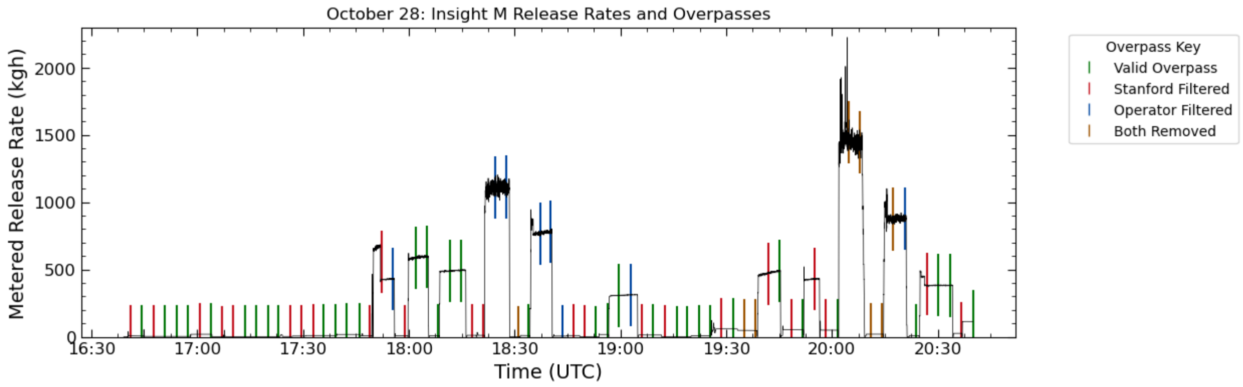

4.2.4 MethaneAIR

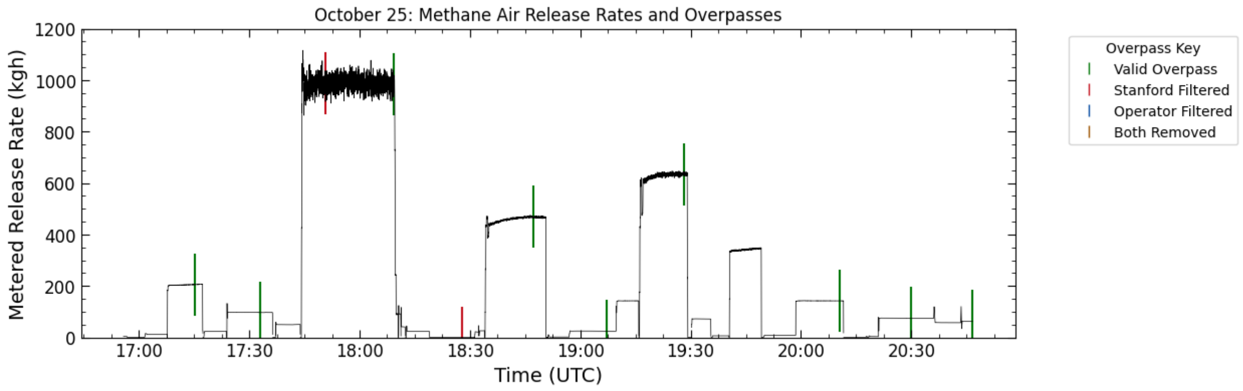

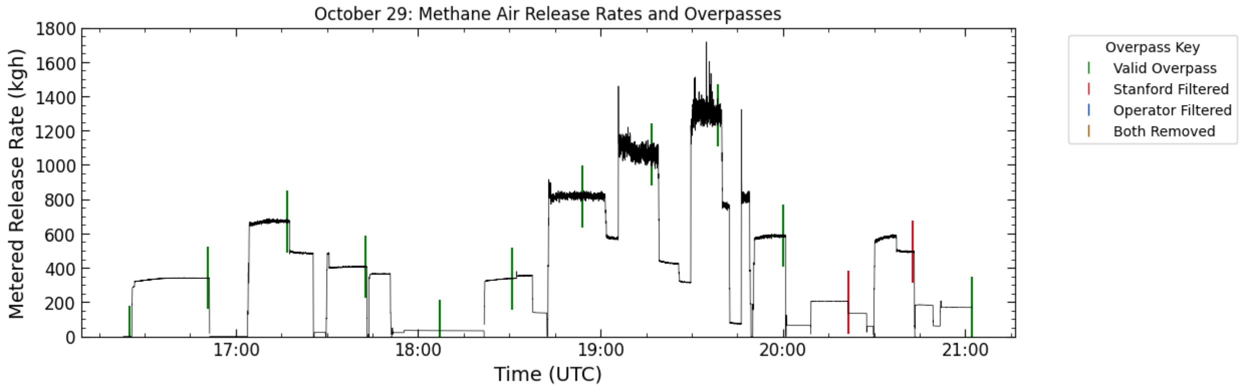

4.2.5 Scientific Aviation

Height of each bar represents the average release rate over the entire measurement period, using start and end points reported by Scientific Aviation. The location of the bar indicates the Scientific Aviation measurement start period.

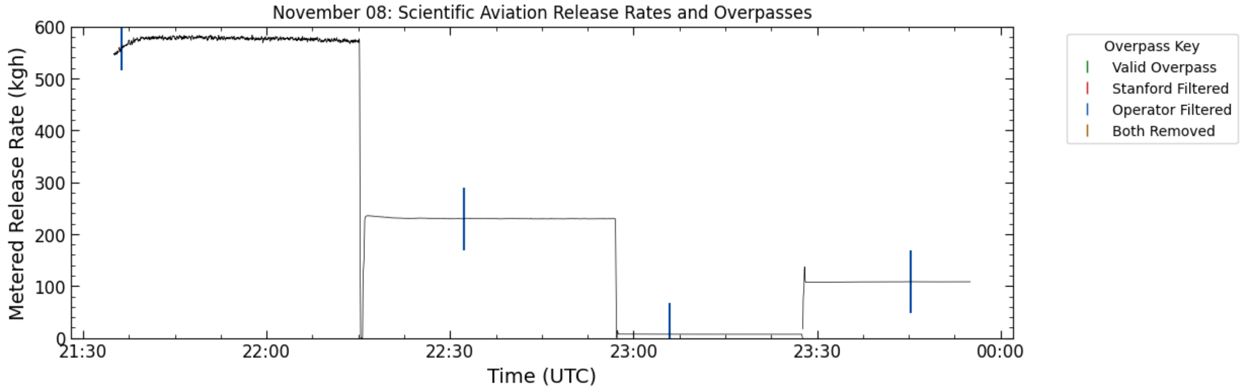

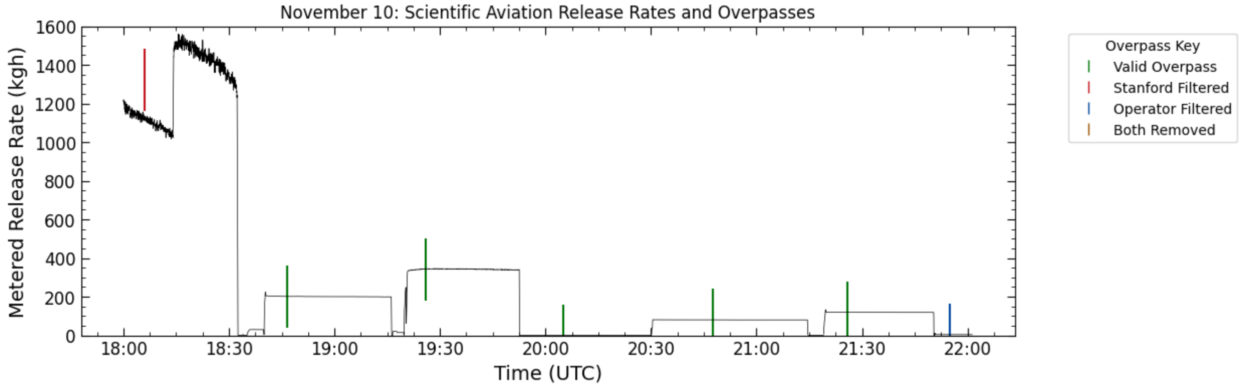

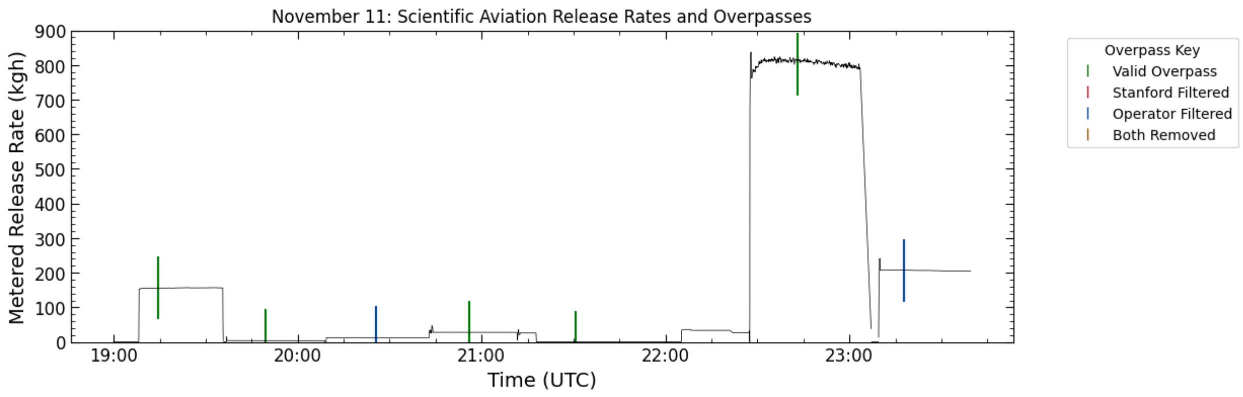
November 11: Scientific Aviation Release Rates and Overpasses
Metered Release Rate (kgh)
0
100
200
300
400
500
600
700
800
900
19:00
20:00
21:00
22:00
23:00
Time (UTC)
Overpass Key
Valid Overpass
Stanford Filtered
Operator Filtered
Both Removed
